# Halogen‐Bonding Strapped Porphyrin BODIPY Rotaxanes for Dual Optical and Electrochemical Anion Sensing

**DOI:** 10.1002/chem.202102493

**Published:** 2021-09-06

**Authors:** Yuen Cheong Tse, Robert Hein, Edward J. Mitchell, Zongyao Zhang, Paul D. Beer

**Affiliations:** ^1^ Chemistry Research Laboratory Department of Chemistry University of Oxford 12 Mansfield Road Oxford OX1 3TA UK

**Keywords:** anion sensing, BODIPY, electrochemical sensing, optical sensing, porphyrins, rotaxanes

## Abstract

Anion receptors employing two distinct sensory mechanisms are rare. Herein, we report the first examples of halogen‐bonding porphyrin BODIPY [2]rotaxanes capable of both fluorescent and redox electrochemical sensing of anions. ^1^H NMR, UV/visible and electrochemical studies revealed rotaxane axle triazole group coordination to the zinc(II) metalloporphyrin‐containing macrocycle component, serves to preorganise the rotaxane binding cavity and dramatically enhances anion binding affinities. Mechanically bonded, integrated‐axle BODIPY and macrocycle strapped metalloporphyrin motifs enable the anion recognition event to be sensed by the significant quenching of the BODIPY fluorophore and cathodic perturbations of the metalloporphyrin P/P^+.^ redox couple.

## Graphical abstract

**The first examples** of porphyrin‐BODIPY halogen bonding [2]rotaxanes have been prepared according to an active metal template strategy. Capitalising on the augmented anion affinities by virtue of axle triazole ligation to macrocyclic zinc(II) metalloporphyrin, the incorporation of fluorogenic BODIPY and redox‐active porphyrin transducing groups allows dual‐channel sensing of anions by quenching BODIPY emission and through cathodic perturbations of porphyrin oxidation redox couples.

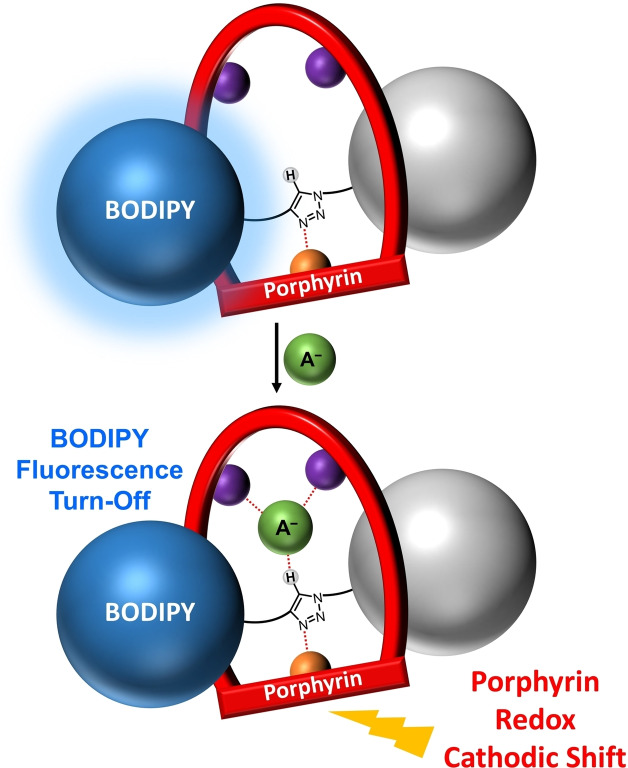

## Introduction

Exploitation of the mechanical bond in supramolecular host–guest chemistry has stimulated the construction of a variety of rotaxane and catenane host systems for charged guest species.^[1]^ Their topological, interlocked three dimensional, biomimetic microenvironments create unique shielded cavities that, notably for target anions of biological and environmental importance, have been demonstrated to exhibit superior affinity and selectivity when compared to non‐interlocked host analogues.^[2]^ The integration of photo‐ or redox‐active reporter groups proximal to the interlocked anion recognition site enables the binding event to be transduced by optical fluorescent,^[3]^ colorimetric^[4]^ and electrochemically responsive methods.^[5]^

The photophysical and redox properties of porphyrins are highly sensitive to their local environments. As a consequence they are prime candidates to function as signal transducers in supramolecular host systems. In particular, taking advantage of the Lewis acidic metal centre of a metalloporphyrin, through judicious design, porphyrin moieties have been incorporated into various anion receptor structural frameworks, such as picket fences,^[6]^ cages,^[7]^ and mechanically interlocked molecules (MIMs).[^4a^, ^5d^, ^8^]

By virtue of its highly desirable photophysical properties including high luminescence quantum yield and tuneable emission wavelength,^[9]^ the BODIPY fluorophore has found numerous applications in areas such as sensing, bioimaging and photovoltaic devices.^[10]^ Notwithstanding this, BODIPY containing MIMs are relatively rare.[^3g^, ^11^]

**Figure 1:**
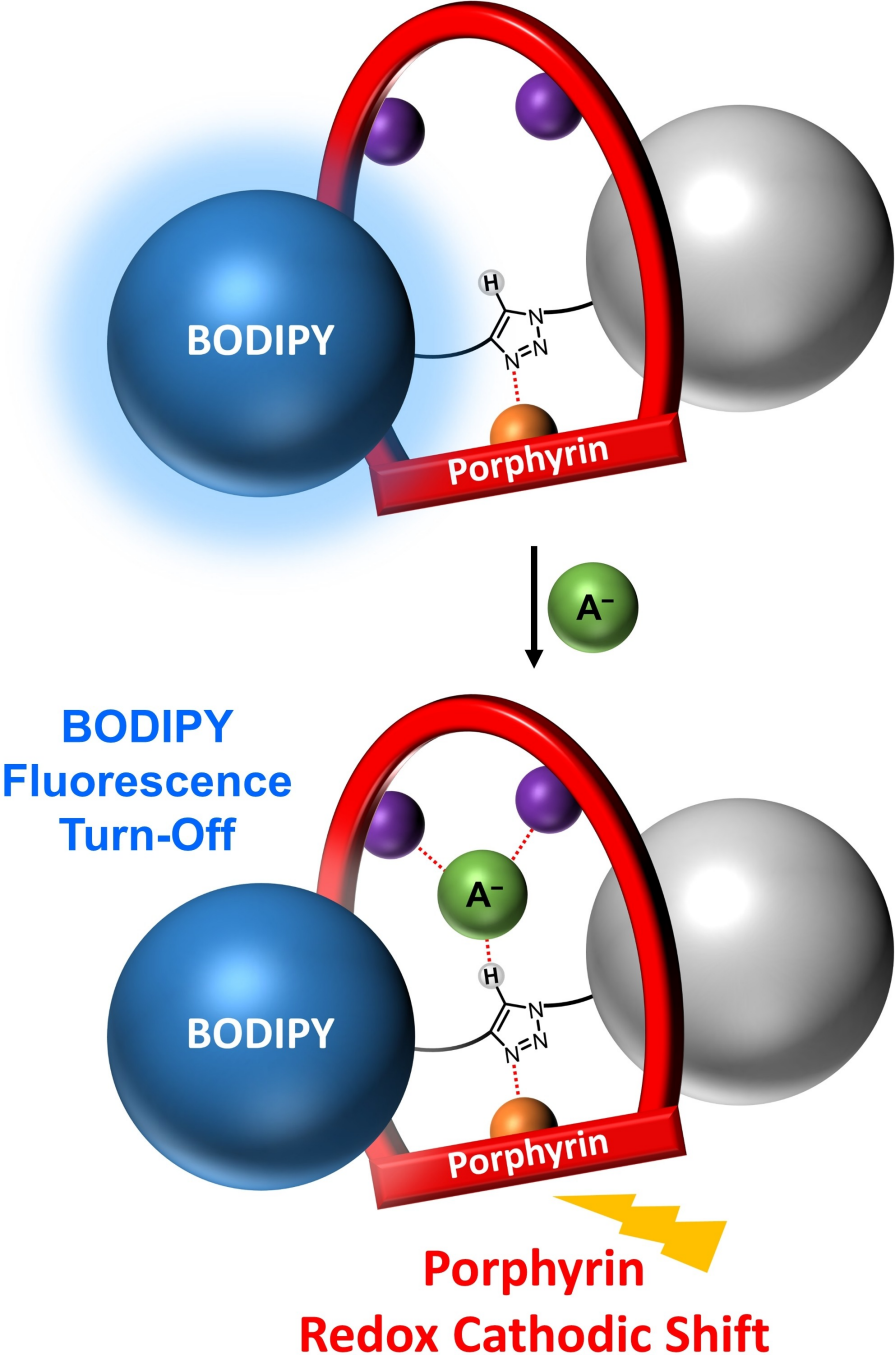
Schematic of the target dual‐signalling metalloporphyrin‐BODIPY [2]rotaxane capable of fluorescent and electrochemical sensing of anions. A^−^=anion; purple spheres represent halogen‐bond‐donor motifs; the orange sphere represents zinc(II).

Herein we report the first examples of dual‐signalling halogen bonding (XB) metalloporphyrin‐BODIPY rotaxanes (Figure 1). These interlocked receptors are shown to be capable of fluorescent and redox electrochemical voltammetric sensing of anions in organic and aqueous mixtures through quenching of BODIPY fluorescence emission and significant cathodic shifts of the porphyrin P/P^+.^ half‐wave potential.

## Results and Discussion

The multistep synthetic strategy undertaken for the preparation of the target porphyrin‐BODIPY rotaxanes entailed the initial preparation of novel XB strapped porphyrins (**1 a**⋅Zn and **1 b**⋅Zn). Their anion recognition and sensing properties were probed by both ^1^H NMR and UV/visible titration studies. The strapped XB porphyrin‐based [2]rotaxane (**2**⋅Zn) and metalloporphyrin‐BODIPY rotaxanes (**3**⋅Zn and **4**⋅Zn). were then synthesised according to an active‐metal template (AMT) methodology by using appropriate terphenyl/BODIPY‐functionalised axle precursors.

### Synthesis of XB strapped porphyrin

**Scheme 1:**
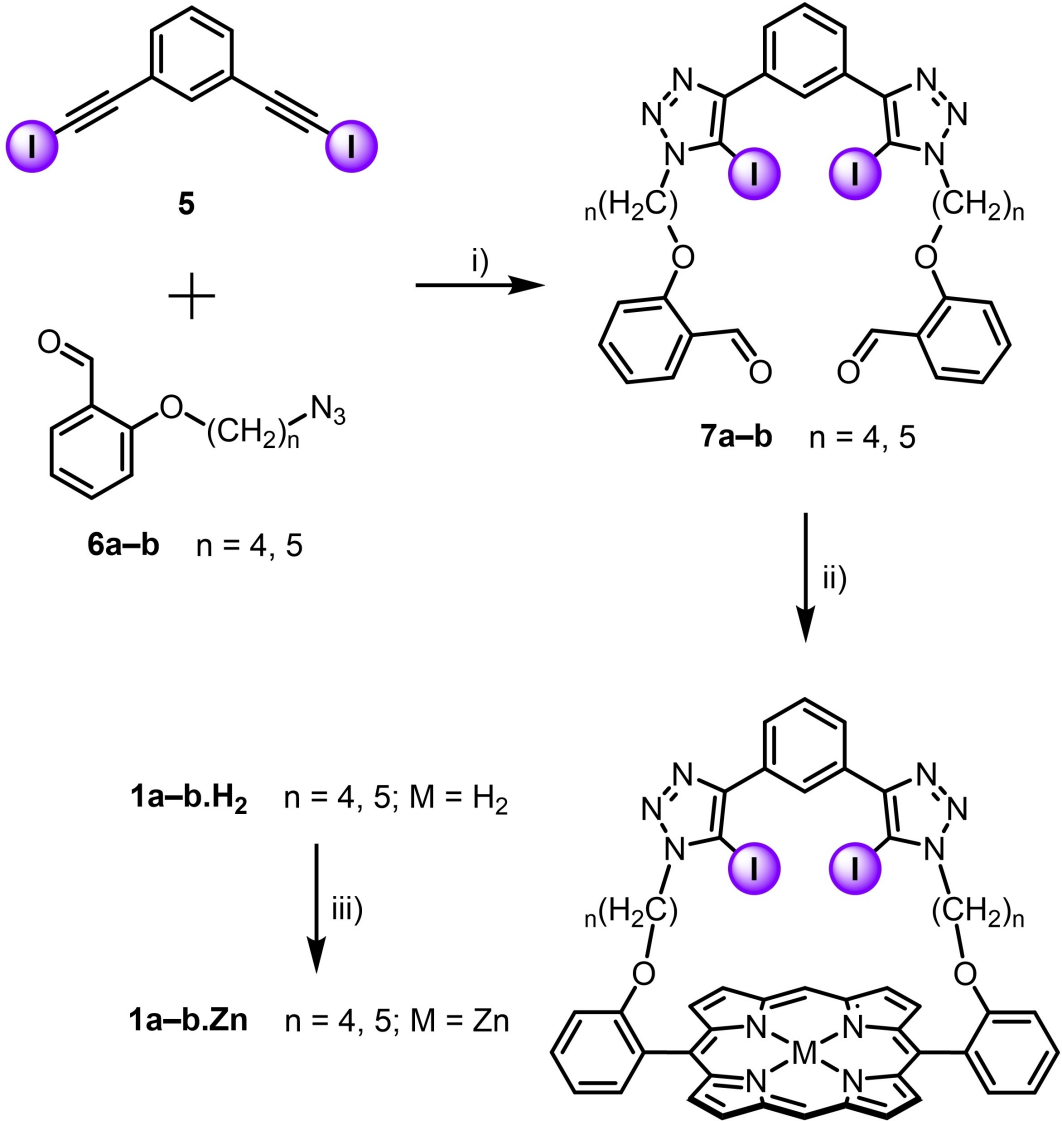
Synthesis of zinc(II) strapped porphyrins **1 a**–**b**⋅Zn. i) [Cu(CH_3_CN)_4_]PF_6_, TBTA, CH_2_Cl_2_, RT, 16 h, 76–93 %; ii) a: 2,2’‐dipyrromethane, TFA, CH_2_Cl_2_, RT, 16 h; b: DDQ, CH_2_Cl_2_, RT, 16 h, 21–43 %; iii) Zn(CH_3_COO)_2_⋅2H_2_O, CH_2_Cl_2_/CH_3_OH, RT, 16 h, quantitative.

Scheme 1 outlines the synthesis of XB strapped porphyrins **1 a**⋅Zn and **1 b**⋅Zn with respective butyl and pentyl groups as covalent linkers between the XB donor sites and the zinc(II) porphyrin core. Cu(I)‐catalysed azide‐alkyne cycloaddition (CuAAC) reaction between 1,3‐bis(iodoethynyl)benzene **5** ^[12]^ and two equivalents of azides **6 a**–**b** ^[13]^ afforded the dialdehydes **7 a**–**b** in good yields. Subsequently, TFA‐catalysed condensation of dialdehydes **7 a**–**b** with 2,2’‐dipyrromethane,^[14]^ followed by in situ oxidation with 2,3‐dichloro‐5,6‐dicyano‐1,4‐benzoquinone (DDQ) gave the cyclised free‐base XB strapped porphyrins **1 a**⋅H_2_ and **1 b**⋅H_2_ in 43 % and 21 % respective yields after chromatographic purification. Quantitative zinc complexation was achieved by reacting the free‐base porphyrins with excess zinc acetate to give strapped XB zinc(II)porphyrins **1 a**–**b**⋅Zn.

### X‐ray diffraction studies of XB strapped porphyrin

**Figure 2:**
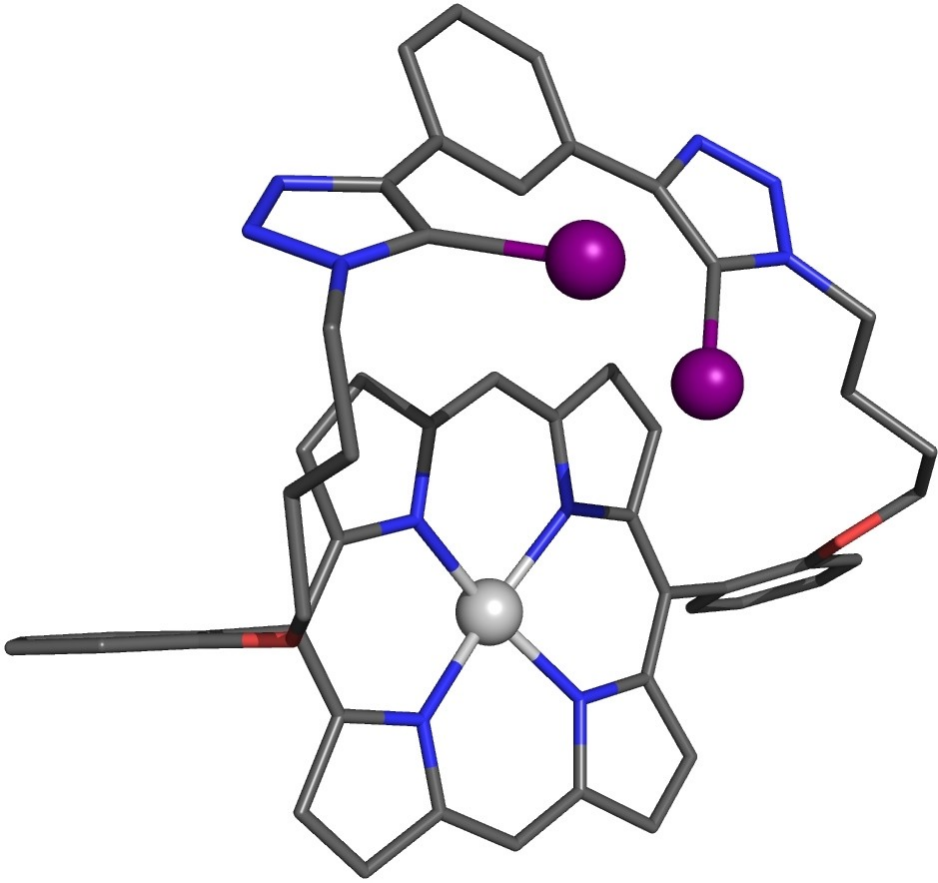
Crystal structure of **1 a**⋅Zn. Hydrogen atoms are omitted for clarity. Grey=carbon, blue=nitrogen, red=oxygen, purple=iodine, silver=zinc.

Single crystals suitable for X‐ray diffraction studies of **1 a**⋅Zn were obtained by slow evaporation of CH_2_Cl_2_/*n*‐hexane solution of the receptor (Figure 2). In the solid‐state, the two iodotriazole groups are twisted out of the plane of the central benzene unit, likely due to the bulky size of the iodine. The strap is also found to bend towards the porphyrin core.

### Anion binding studies of XB strapped porphyrins

**Figure 3:**
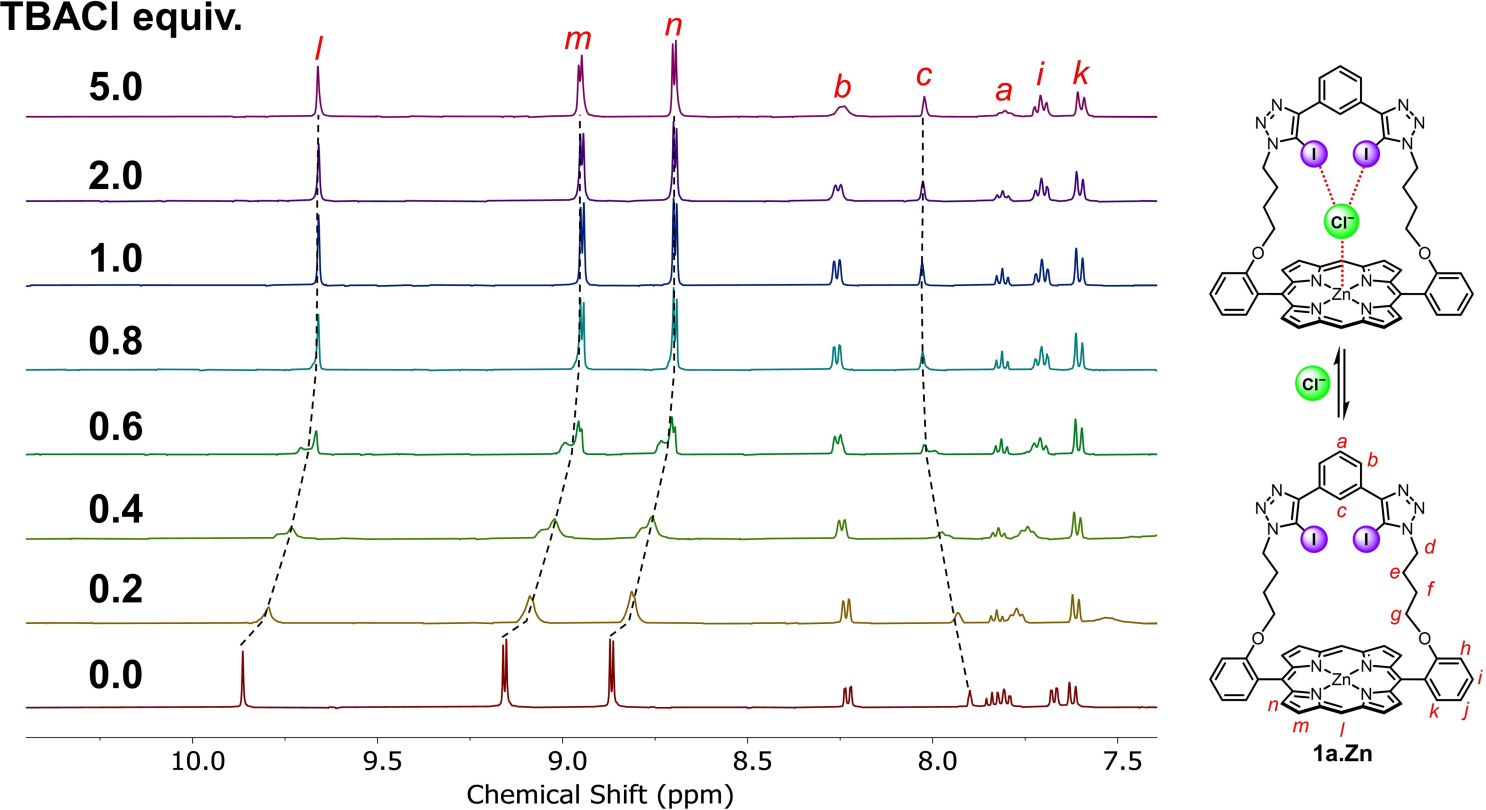
Truncated ^1^H NMR spectra of zinc(II) strapped porphyrin **1 a**⋅Zn upon addition of 0–5 equivalents of TBACl (500 MHz, 298 K, [D_6_]acetone, [**1 a**⋅Zn]=1 mM).

^*1*^ *H NMR experiments*: To probe the anion binding properties of the XB strapped porphyrin receptors, a preliminary ^1^H NMR titration of **1 a**⋅Zn with TBACl in [D_6_]acetone was carried out (Figure 3). A general upfield perturbation of the porphyrin's *meso*‐ (*l*) and β‐ (*m* and *n*) proton signals was observed upon the addition of the halide anion, likely caused by the donation of electron density from the axially zinc metalloporphyrin coordinated Cl^−^.^[15]^ The internal benzene proton *c* was also found to shift downfield, suggesting Cl^−^ was bound by the bis‐iodotriazole XB donors inside the cavity of the strapped porphyrin. Interestingly, proton signals *l*–*n* and *c* also broadened, split, and merged again during the addition of up to one equivalent of Cl^−^. While the origin for the splitting is unclear, it is thought to be related to the presence of both the electrophilic XB donors and Lewis acidic zinc(II) centre of the strapped cavity competing for the halide guest through different modes of binding. No further chemical shifts were observed after addition of one equivalent of Cl^−^, thus suggesting strong binding. By monitoring the chemical shift of internal benzene proton *c*, Bindfit^[16]^ analysis using a 1 : 1 host–guest binding model determined the Cl^−^ association constant of XB strapped porphyrin **1 a**⋅Zn as >10^5^ M^−1^ (Figure S5‐8 in the Supporting Information). Attempts to carry out an analogous chloride anion titration with the longer‐strap **1 b**⋅Zn, however, were thwarted, due to the macrocycle's unexpected insolubility in acetone, despite its structural similarity to the shorter‐strap **1 a**⋅Zn. Subsequent electrochemical investigations provided evidence for the relatively longer pentyl side‐arms of **1 b**⋅Zn enabling intramolecular coordination of the triazole groups to the zinc(II) porphyrin centre (see below).[^6b^, ^17^]

**Figure 4:**
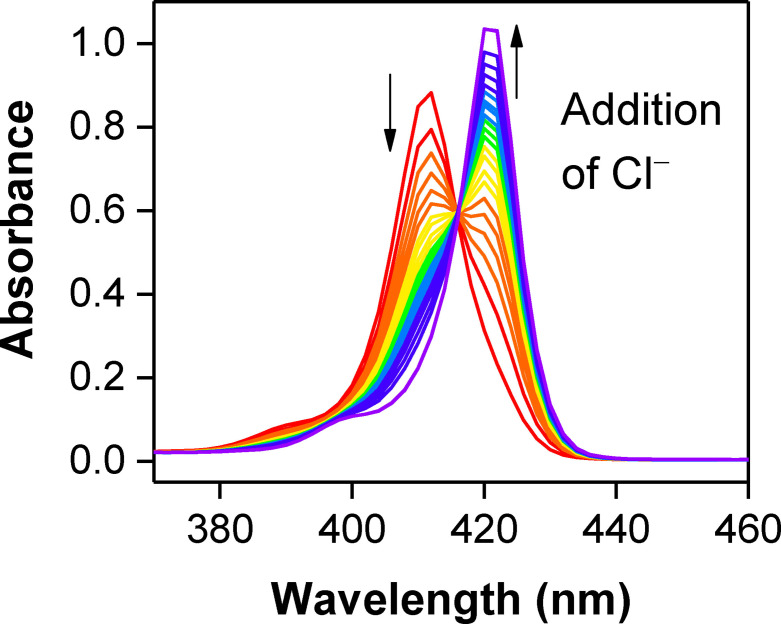
Changes in the Soret band of **1 a**⋅Zn upon addition of 10 equiv. of TBACl (298 K, acetone, [**1 a**⋅Zn]=3 μM).

*UV/visible absorption experiments*: To assess the anion sensing capability of **1 a**⋅Zn, UV/visible titrations of **1 a**⋅Zn with TBA halide salts were undertaken in acetone. The strapped porphyrin **1 a**⋅Zn exhibits an intense Soret band absorption at 412 nm and Q bands around 500–600 nm. The addition of TBA halides to **1 a**⋅Zn caused significant red shifts to both the Soret and Q bands. In the titration with TBACl, the porphyrin's Soret band at 412 nm displayed a bathochromic shift to 423 nm with a clear isosbestic point at 416 nm (Figure 4). This red‐shift is consistent with axial ligation of Cl^−^ to the Lewis acidic zinc(II) metalloporphyrin.^[15]^ Analogous titration spectra were observed upon addition of Br^−^ and I^−^, implying similar anion coordination behaviour (Figures S6‐1 and −2). Bindfit^[16]^ analysis determined 1 : 1 stoichiometric host–guest halide association constants for **1 a**⋅Znby monitoring the change in Soret band absorbance at 412 nm upon addition of halides (Table 1). For comparison purposes, binding constants for the strap‐free zinc(II) 5,10,15,20‐tetraphenylporphyrin (ZnTPP) are also tabulated.^[7a]^

**Table 1:** Anion association constants (*K*_a_ [M^**−**1^]) for **1 a**⋅Zn and ZnTPP with TBA halide salts in acetone as determined by UV/visible titrations.^[a]^

Anion	**1a**⋅Zn	ZnTPP^[7a]^	Binding enhancement factor *K*_a_(**1a**⋅Zn)/*K*_a_(ZnTPP)
Cl^−^	4.1×10^5^	8.1×10^3^	5.1×10^1^
Br^−^	1.5×10^6^	1.5×10^2^	1.0×10^4^
I^−^	9.4×10^4^	5.0×10^1^	1.9×10^3^

[a] *K*_a_ values calculated from global fitting of UV/visible titration data using Bindfit software with a 1 : 1 host–guest binding model.^[16]^ Errors (±) are all <10 %. All anions added as their TBA salts. [**1 a**⋅Zn]=3 μM. Solvent=acetone. *T*=298 K.

As expected, the halide anion association constants for strapped porphyrin **1 a**⋅Zn are all notably much higher than the strap‐free ZnTPP, highlighting the importance of the XB strap in augmenting the anion binding strength. The greatest binding enhancement was seen for Br^−^, where the *K*_a_ was found to increase 10 000‐fold in the XB strapped receptor. In addition, **1 a**⋅Zn displayed selectivity for Br^−^ over Cl^−^ and I^−^ while ZnTPP showed a preference for the more charge‐dense Cl^−^. The substantial enhancement in binding strength and selectivity of **1 a**⋅Zn for Br^−^ can be rationalised by both size and shape complementarity between the receptor and anion, as well as the interplay between the attractive interaction of Br^−^ with the two iodotriazole XB donor groups and its endotopic axial ligation to the Lewis acidic zinc(II) porphyrin.^[15]^ In conclusion, this data suggests that the halide affinities of zinc(II) metalloporphyrin can be substantially enhanced by the presence of the XB strap.

### Synthesis of [2]rotaxanes

Active‐metal template synthesis (AMT) has presented itself as a powerful strategy to construct a plethora of mechanically interlocked structures since its inception in 2006.^[18]^ The *meta*‐substituted bis(iodotriazole)benzene motif, when incorporated into a macrocycle, has been reported to coordinate to Cu(I) endotopically through the basic triazole nitrogen.[^5f^, ^13^, ^19^] This allows the subsequent CuAAC click reaction between the axle precursors through the cavity of the macrocycle. Moreover, a phenanthroline‐strapped zinc(II) metalloporphyrin has also been reported to assist in preorganisation of the azido precursor through metal ligation in CuAAC‐AMT reactions.^[20]^ Strapped porphyrin receptor **1 a**⋅Zn was therefore expected to be capable of synthesising [2]rotaxanes by CuAAC‐AMT.

**Scheme 2:**
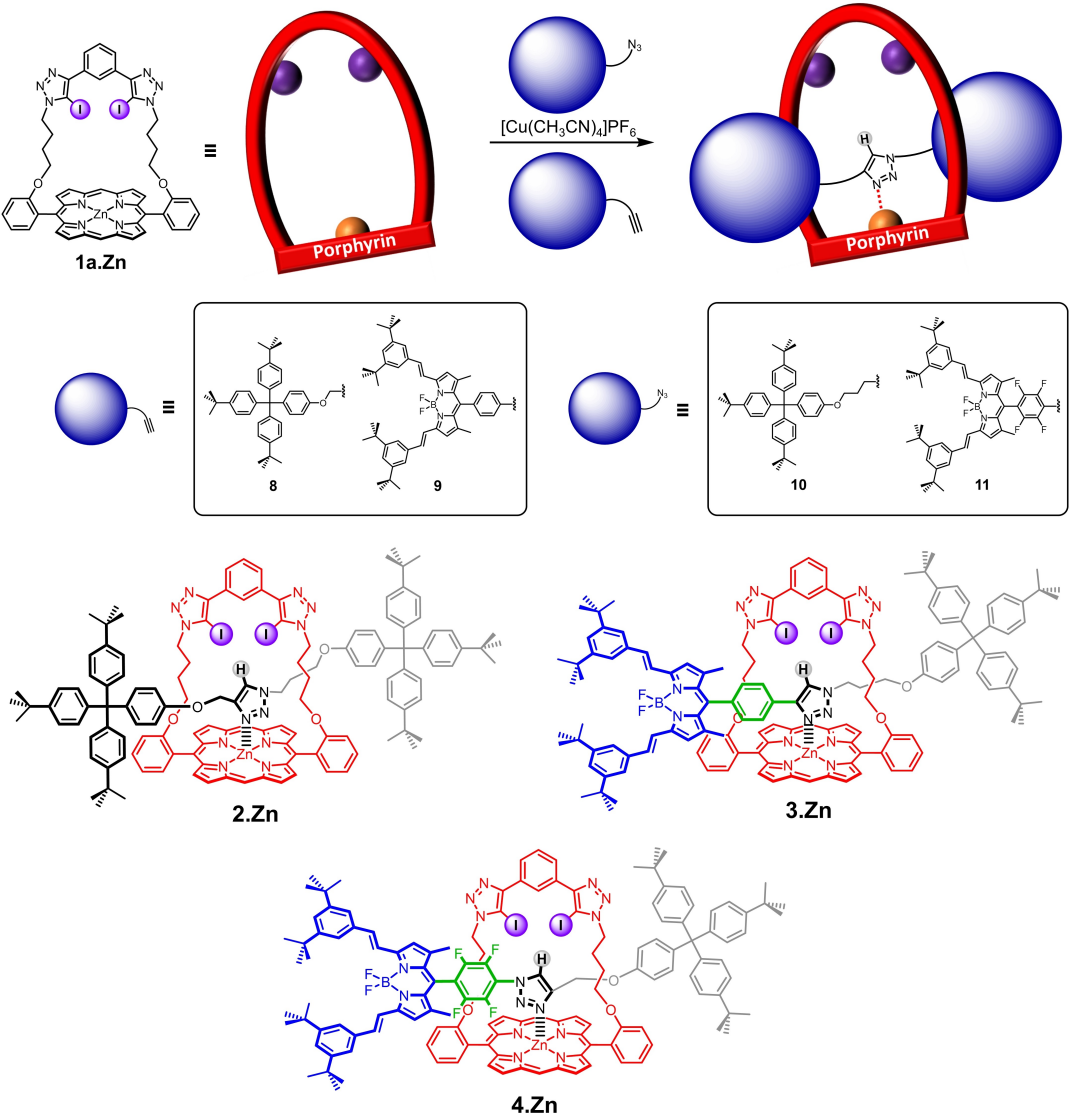
Synthesis of **2**–**4**⋅Zn following the general CuAAC‐AMT procedure.

In an initial attempt, **1 a**⋅Zn was treated with terphenyl‐stopper alkyne **8** and stopper azide **10** under CuAAC‐AMT conditions (Scheme 2). Pleasingly, after heating at 40 °C for two days in CH_2_Cl_2_, and chromatographic purification, [2]rotaxane **2**⋅Zn was isolated in an excellent yield of 63 %. This high yield for rotaxanation is postulated to originate from the synergistic effect of the endotopic coordination of Cu(I) by iodotriazole and, importantly, azide positioning mediated by zinc(II) metalloporphyrin.^[20]^ BODIPY‐stoppered rotaxanes **3**⋅Zn and **4**⋅Zn were also constructed using BODIPY‐stopper alkyne **9** and BODIPY‐stopper azide **11** in 35–37 % yields following the general procedures for CuAAC‐AMT (Scheme S1‐2) for synthesis of **9** and **11**). This further demonstrates the versatility of **1 a**⋅Zn to synthesise sophisticated interlocked systems. All three novel [2]rotaxanes **2**–**4**⋅Zn were fully characterised by ^1^H, ^13^C, ^19^F NMR (where relevant) and high resolution ESI mass spectroscopy (Figures S2‐31 to −40). Longer‐strap porphyrin **1 b**⋅Zn was also subjected to the standard CuAAC‐AMT conditions. Complete consumption of axle precursors **8** and **10** was observed after heating at 40 °C overnight but no formation of interlocked structure was observed. This could be explained by the intramolecular coordination of triazole nitrogen to the zinc(II) porphyrin, which prevented the triazole N‐mediated endotopic binding of Cu(I) by the macrocycle.

**Figure 5:**
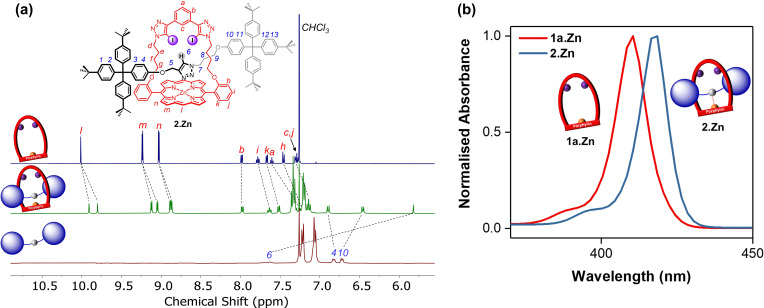
a) Comparative truncated ^1^H NMR spectra showing the aromatic region of strapped porphyrin **1 a**⋅Zn (top), [2]rotaxane **2**⋅Zn (middle) and the corresponding axle (bottom; 500 MHz, 298 K, [D_6_]acetone). b) Normalised electronic absorption spectra showing the Soret bands of strapped porphyrin **1 a**⋅Zn and [2]rotaxane **2**⋅Zn ([**1 a**⋅Zn]=[**2**⋅Zn]=3 μM, 298 K, acetone).

A comparison of ^1^H NMR spectra of terphenyl‐stoppered [2]rotaxane **2**⋅Zn and strapped porphyrin **1 a**⋅Zn in [D_6_]acetone is shown in Figure 5a. Firstly, a splitting of porphyrin aromatic signals *l*, *m* and *n* was observed, which is suggestive of a restricted motion of the strapped porphyrin macrocycle component along the asymmetric axle at ambient temperature. Secondly, the triazole proton *6* appears at 6.2 ppm, which is unusually shifted upfield compared to triazole signals of the non‐interlocked axle and analogous porphyrin‐free rotaxanes (typically>7 ppm).[^13^, ^18a^, ^18b^, ^21^] This was attributed to a coordination of the axle triazole heterocycle to the zinc(II) porphyrin, resulting in the triazole proton *6* being located directly in the shielding region of the porphyrin's ring current.^[6b]^ This is further attested by comparing the UV/visible absorption spectra of [2]rotaxane **2**⋅Zn and strapped porphyrin **1 a**⋅Zn (Figure 5b), where the coordination of the axle induced a red shift (ca. 6 nm) of porphyrin absorption bands in the rotaxane system. Furthermore, the addition of 1 % of pyridine to the solution of **1 a**⋅Zn induced a bathochromic shift to the Soret band, arising from pyridine ligation to zinc(II) porphyrin. However, negligible shift was observed in the absorption spectrum of **2**⋅Zn upon addition of pyridine, indicating the axle triazole is coordinating to the macrocyclic zinc(II) porphyrin in the rotaxane framework, and is not displaced by the presence of pyridine (Figure S4‐3). For the BODIPY‐stoppered [2]rotaxanes **3**–**4**⋅Zn, similar ^1^H NMR spectra and UV/visible absorption bands were observed (Figures S4‐1 to −3), suggesting they adopt similar conformations as **2**⋅Zn with the axle triazole coordinating to zinc(II) porphyrin in the mechanically interlocked structures. This coordinate interaction between rotaxane macrocyclic and axle components is envisaged to both preorganise the rotaxane binding sites and polarise the axle triazole C−H bond donor, thereby potentially enhance anion binding.

### Anion binding studies of [2]rotaxanes

**Figure 6:**
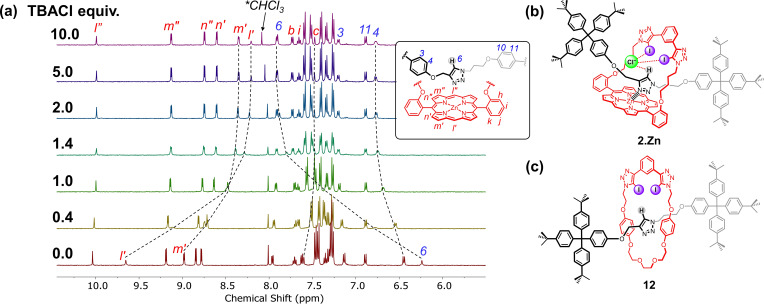
a) Truncated ^1^H NMR titration spectra of [2]rotaxane **2**⋅Zn upon addition of TBACl (500 MHz, 298 K, [D_6_]acetone, [**2**⋅Zn]=1 mM). b) Postulated Cl^−^ binding mode of **2**⋅Zn with an anion perching on one side of the rotaxane cavity. c) Reference [2]rotaxane **12** with tri(ethylene glycol) instead of porphyrin as the macrocycle linker.^[13]^

^*1*^ *H NMR experiments*: Quantitative anion binding studies of the terphenyl‐stoppered [2]rotaxane **2**⋅Zn were conducted by ^1^H NMR titrations in [D_6_]acetone. Upon addition of aliquots of TBACl, aromatic protons on only one side of the porphyrin *m*’ and *l*’ exhibited significant upfield shifts whilst only slight perturbations were observed for their counterparts *m”* and *l”* (Figure 6a). A downfield shift of peripheral stopper proton *4* was also observed. These observations suggest the bound Cl^−^ was likely perching between the plane of strapped porphyrin and one of the bulky stopper groups (Figure 6b). Evidence of HB between the axle triazole proton and Cl^−^ is provided by the significant downfield shift (Δ*δ*=1.7 ppm) of proton *6*. Similar proton shifts were observed in the presence of Br^−^ and I^−^, suggesting the rotaxane adopts an analogous conformation upon halide binding (Figures S5‐2 to −7). Anion association constants of [2]rotaxane **2**⋅Zn in [D_6_]acetone, and the more competitive 2 % D_2_O/[D_6_]acetone, were determined by fitting of titration data using Bindfit^[16]^ employing a 1 : 1 stoichiometric host–guest binding model and are summarised in Table 2. For comparison, the association constants for the structurally similar porphyrin‐free [2]rotaxane **12** are also tabulated (Figure 6c).^[13]^

**Table 2:** Anion association constants (*K*_a_ [M^−1^]) for **2**⋅Zn and **12** with TBA halide salts in [D_6_]acetone and 2 % D_2_O/[D_6_]acetone determined by ^1^H NMR titrations.^[a]^

	[D_6_]Acetone		2 % D_2_O/[D_6_]Acetone		
Anion	**2**⋅Zn	**12**	**2**⋅Zn	**12**	*K*_a_(**2**⋅Zn)/*K*_a_(**12**)
Cl^−^	>10^4^	4 380	1 290	110	12
Br^−^	>10^4^	2 140	3 550	370	10
I^−^	>10^4^	900	3 230	530	6

[a] *K*_a_ values calculated using Bindfit with a 1 : 1 host–guest binding model.^[16]^ Errors (±) are all <10 %. All anions added as their TBA salts. [**2**⋅Zn]=1.0 mM. *T*=298 K.

In [D_6_]acetone, the association constants of **2**⋅Zn towards all halides (Cl^−^, Br^−^ and I^−^) were >10^4^ M^−1^, significantly higher than that of the porphyrin‐free analogue **12**. The presence of 2 % D_2_O (*v*/*v*) in [D_6_]acetone attenuated the anion affinity for both receptors, however, the porphyrinoid rotaxane host **2**⋅Zn still displayed 6‐ to 10‐fold enhancements in halide binding compared to rotaxane **12**, highlighting the significant favourable contribution of the axle triazole‐metalloporphyrin coordinate interaction to augment the receptor's halide affinity. Interestingly, whilst in the aqueous organic solvent the reference rotaxane receptor **12** displayed Hofmeister bias selectivity for I^−^, rotaxane **2**⋅Zn exhibited a preference for Br^−^. This may be explained by the presence of the more rigid porphyrin (**2**⋅Zn) instead of the flexible tri(ethylene glycol) (**12**) in the macrocyclic component, restricting the size of the binding cleft and weakening the affinity towards the larger I^−^.

*UV/visible absorption experiments*: UV/visible anion titrations were carried out with **2**–**4**⋅Zn in acetone. Surprisingly, no significant perturbations in porphyrin absorption bands of the respective rotaxane were observed after the addition of excess (>1000 equiv) halides for all three receptors. This suggests, that although axle triazole coordination to zinc(II) site preorganises the anion binding site and improves the halide affinity of the host, the concomitant saturation of the zinc(II) porphyrin's coordination environment appears to negate its ability to function as a chromophoric reporting group.

**Figure 7:**
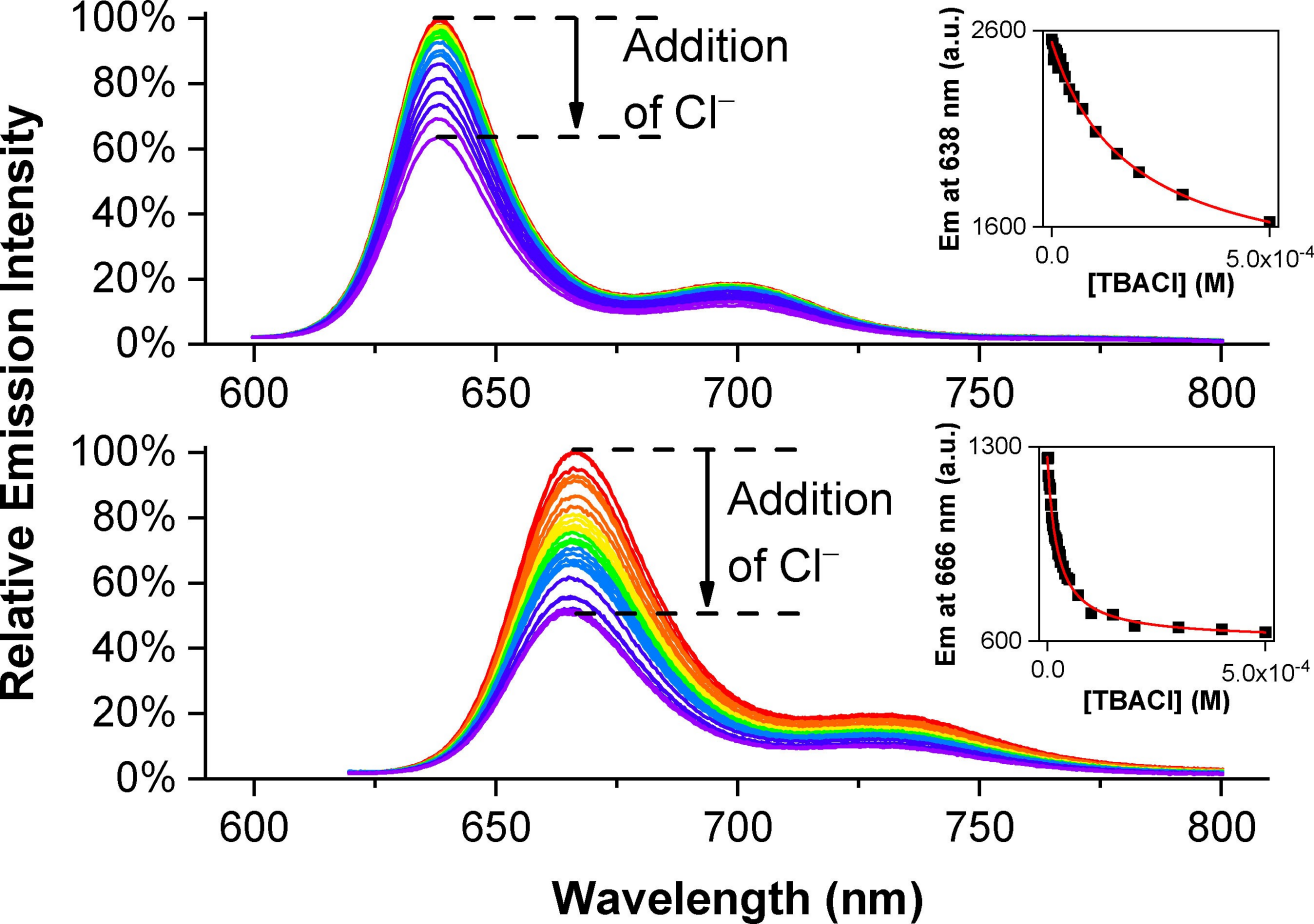
Change in BODIPY emission of **3**⋅Zn (top) and **4**⋅Zn (bottom) upon addition of 500 equiv. of TBACl ([**3**⋅Zn]=[**4**⋅Zn]=1 μM, *λ*_ex_=575 (**3**⋅Zn) and 595 nm (**4**⋅Zn), 298 K, acetone). Insets: change in emission as a function of anion concentration.

*Fluorescence experiments*: The incapability of the respective zinc(II) porphyrin rotaxane component to act as an anion binding chromophore reporting group prompted us to incorporate the fluorescent BODIPY transducer into the axle component. In acetone, receptors **3**⋅Zn and **4**⋅Zn were found to be highly fluorescent and exhibit typical BODIPY emission maxima at 638 and 666 nm, respectively. The emission peaks of **4**⋅Zn appear at longer wavelength (28 nm) compared to that of **3**⋅Zn, presumably due to the presence of electron‐withdrawing perfluoroaryl group appended to the BODIPY core. Preliminary qualitative fluorescence titration experiments of both receptors with TBACl were conducted in acetone, whereby addition of up to 500 equivalents of Cl^−^ induced emission quenching by approximately 37% and 50 % for **3**⋅Zn and **4**⋅Zn, respectively (Figure 7). It is also noteworthy that the introduction of the axle perfluoroaryl group greatly enhanced the magnitude of quenching. By monitoring the change in BODIPY emission as a function of anion concentrations, association constants of **3**–**4**⋅Zn for a range of anions (Cl^−^, Br^−^, I^−^, OAc^−^ and SO_4_^2−^) were determined by global fitting fluorescence titration data (Figures S7‐1 to −6) and are summarised in Table 3.

**Table 3:** Anion association constants (*K*_a_ [M^−1^]) for **3**⋅Zn and **4**⋅Zn with TBA halide salts in acetone and 2 % H_2_O/acetone determined by fluorescence titrations.^[a]^

	Acetone		2 % H_2_O/Acetone	
Anion	**3**⋅Zn	**4**⋅Zn	**3**⋅Zn	**4**⋅Zn
Cl^−^	5 440	44 910	n.b.	1090
Br^−^	2 280	35 240	n.b.	650
I^−^	–^[b]^	4 820	n.b.	n.b.
OAc^−^	29 940	58 310	n.b.	n.b.
SO_4_^2−^	13 820	39 220	n.b.	n.b.

[a] *K*_a_ values calculated using global fitting of fluorescence titration spectra using Bindfit with a 1 : 1 host–guest binding model.^[16]^ Errors (±) are all <10 %. All anions added as their TBA salts. *T*=298 K. [b] No reliable association constants could be obtained. n.b.: No binding.

For spherical halides, both receptors display a strong preference for Cl^−^ over Br^−^ in acetone, with *K*_a_ (Cl^−^)=5440 M^−1^ and 44910 M^−1^ for **3**⋅Zn and **4**⋅Zn, respectively. This may be rationalised on the basis of anion basicity, with Cl^−^ bearing higher charge density. Notably, the presence of the axle perfluoroaryl group directly linked to the triazole in **4**⋅Zn led to an 8‐ to 15‐fold increase in the binding of Cl^−^ and Br^−^ respectively when compared to **3**⋅Zn, attributable to its inductive electron‐withdrawing effect making the triazole C−H proton more acidic and a stronger HB donor. Interestingly, both rotaxanes also display high affinities for oxoanions OAc^−^ and SO_4_^2−^, with association constant magnitudes comparable to or larger than for Cl^−^. This suggests, in spite of structural rigidification induced by axle triazole coordination to the zinc metalloporphyrin, the rotaxanes still possess a certain degree of flexibility to allow binding of the bulkier oxoanions. Analogous fluorescence titrations were also conducted in the more competitive 2 % H_2_O/acetone. While no perturbations to emission were observed for **3**⋅Zn upon addition of anions, the perfluoroaryl‐axle containing rotaxane **4**⋅Zn still displayed a significant quenching response towards Cl^−^ and Br^−^, maintaining a preference for the lighter halide anion (*K*_a_(Cl^−^)/*K*_a_(Br^−^)=1.7).

**Figure 8:**
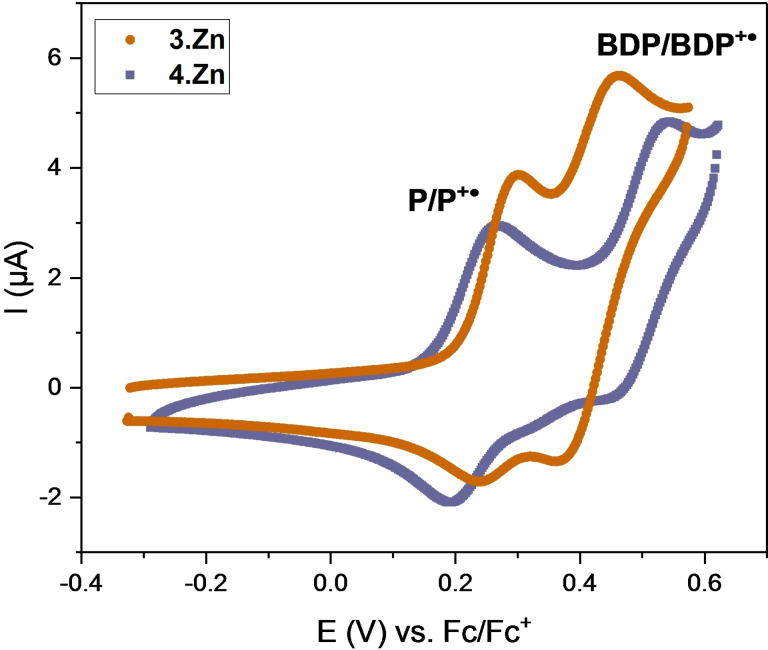
CVs of **3**⋅Zn (tan) and **4**⋅Zn (purple) in CH_2_Cl_2_/TBAPF_6_ at a scan rate of 100 mV/s.

*Electrochemical investigations*: The electrochemical properties of strapped porphyrins **1 a**–**b**⋅Zn and rotaxanes **2**–**4**⋅Zn were studied by cyclic voltammetry in CH_2_Cl_2_. All these receptors display reversible/quasi‐reversible one‐electron oxidative porphyrin P/P^+.^ redox couples (Figures S8‐1 to −7). At more anodic potential, an additional redox couple, arising from further one‐electron oxidation of porphyrin (P^+.^/P^2+^) was also observed (Table 4). However, for all receptors this couple displayed poor reversibility and was not studied in more detail. For BODIPY‐stoppered rotaxanes, **3**⋅Zn and **4**⋅Zn, in addition to the porphyrin‐centred oxidation, another quasi‐reversible redox couple was observed at slightly more anodic potentials (Figure 8, Table 4). By comparison with BODIPY‐stoppers **9** and **11**, this redox process was assigned to be the oxidation of the BODIPY motif, yielding the monocationic radical (BDP/BDP^+.^; Figures S8‐4 to −8, Table S8‐1).

**Table 4:** Half‐wave potentials *E*_1/2_ (V vs. Fc/Fc^+^) for all receptors in CH_2_Cl_2_, 100 mM TBAPF_6_ in the absence and presence of pyridine (1 % *v*/*v*).

*E*_1/2_ in CH_2_Cl_2_/TBAPF_6_	P/P^+.^	P^+.^/P^2+[a]^	BDP/BDP^+.^
**1a**⋅Zn	0.282 V	0.624 V	/^[b]^
**1b**⋅Zn	0.179 V	0.617 V	/^[b]^
**2**⋅Zn	0.193 V	0.652 V	/^[b]^
**3**⋅Zn	0.270 V	0.688 V	0.425 V
**4**⋅Zn	0.230 V	0.675 V	0.501 V
**1a**⋅Zn+1 % (*v/v*) pyridine	0.267 V	0.567 V	/^[b]^
**1b**⋅Zn+1 % (*v/v*) pyridine	0.270 V	0.552 V	/^[b]^
**2**⋅Zn+1 % (*v/v*) pyridine	0.204 V	0.550 V	/^[b]^
**3**⋅Zn+1 % (*v/v*) pyridine	0.292 V	0.609 V	n.o.
**4**⋅Zn+1 % (*v/v*) pyridine	0.248 V	0.579 V	≈0.47^[c]^

[a] Poor reversibility. [b] Not applicable. [c] Weak and broad. n.o.:Not observed.

**Figure 9:**
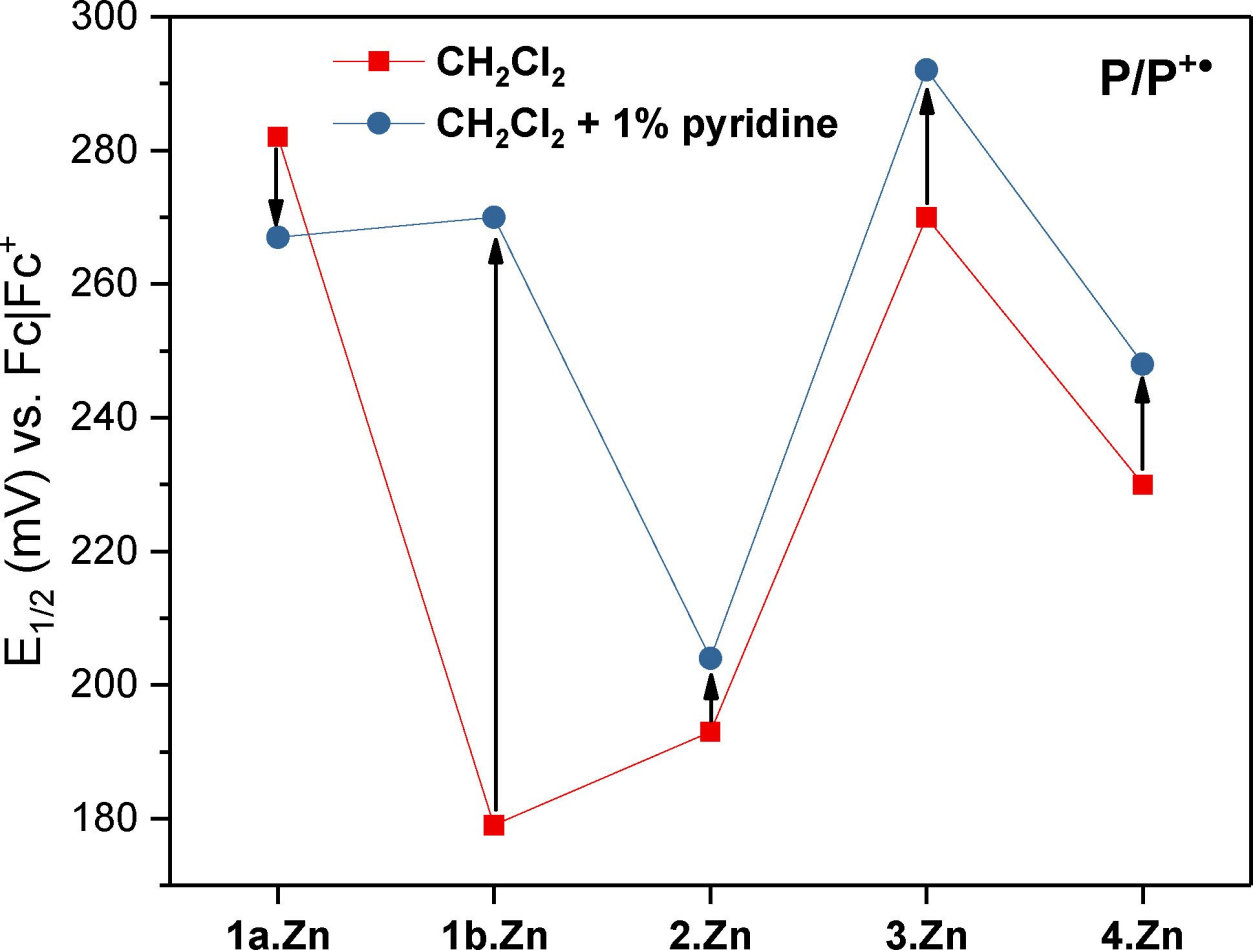
Half‐wave potentials of the P/P^+.^ couple of all receptors in CH_2_Cl_2_/TBAPF_6_ in the absence (▪squares) and presence (•circles) of 1 % (*v*/*v*) pyridine. Black arrows indicate the change in potential upon addition of pyridine. Connecting lines are to guide the eye only.

The half‐wave potential *E*_1/2_ for all receptors in CH_2_Cl_2_ in the absence and presence of pyridine (1 % *v*/*v*) are tabulated in Table 4 and the *E*_1/2_ of P/P^+.^ are plotted in Figure 8. Interestingly, the half‐wave potential *E*_1/2_ of the P/P^+.^ couple of the receptors is significantly different, even for structurally closely related compounds (Figure S8‐9). For example, the short‐strap porphyrin macrocycle **1 a**⋅Zn exhibits an *E*_1/2_ of 0.282 V, while the analogous, long‐strap **1 b**⋅Zn containing only one additional methylene linker is oxidised at a significantly more cathodic potential of 0.179 V. This large stabilisation of the P/P^+.^ couple cannot arise from through‐bond electronic stabilisation (as the substituents are electronically identical), but has to arise from other effects. Specifically, we propose that intramolecular triazole coordination to the Zn centre might account for these observations. This is further confirmed by addition of pyridine (1 % *v*/*v*); in this case both compounds display identical half‐wave potentials (≈0.270 V) for the P/P^+.^ couple (Figure 9). In the case of **1 a**⋅Zn, this corresponds to a small stabilisation (cathodic shift by 15 mV), arising from pyridine coordination at Zn. For **1 b**⋅Zn, this corresponds to a significant destabilisation (≈90 mV), indicative of ligand displacement of the triazole by pyridine.

The *E*_1/2_ of the rotaxane **2**⋅Zn (*E*_1/2_=0.193 V) is significantly lower than that of the free macrocycle **1 a**⋅Zn (*E*_1/2_=0.282 V), and similar to that of the strapped porphyrin macrocycle with the longer strap **1 b**⋅Zn (*E*_1/2_=0.179 V). Considering that coordination of the macrocycle's triazole in the presence of an axle component is highly unlikely, this is strongly suggestive of P/P^+.^ stabilisation by axle triazole coordination. This was again confirmed by analysis of the *E*_1/2_ in the presence of pyridine, which was largely unaltered (0.204 V). This behaviour is in marked contrast to that of the free macrocycle alone, suggesting that in the rotaxane, pyridine cannot displace the triazole ligand. This confirms that the stabilisation of the P/P^+.^ couple in **2**⋅Zn does not arise from the macrocycle's triazole (which can be displaced by pyridine as shown for **1 b**⋅Zn) but instead from coordination of the axle triazole, which for kinetic reasons cannot be displaced by pyridine. Similar electrochemical behaviour was observed for the BODIPY‐stoppered rotaxanes **3**–**4**⋅Zn, where the addition of pyridine induced only small anodic shifts to the *E*_1/2_ of the P/P^+.^ couple, indicating pyridine is, again, unable to significantly compete with axle triazole coordination in the interlocked systems.

Interestingly, the second porphyrin oxidation couple P^+.^/P^2+^, in stark contrast to the afore‐discussed P/P^+.^ couple, displayed a significant and consistent cathodic shift (≈60–70 mV) upon addition of pyridine for all the receptors (Figure S8‐10). This observation indicates that, regardless of initial triazole coordination, additional pyridine coordination enhances the electron density at the zinc(II) porphyrin for the P^+.^/P^2+^ oxidation. As a proof‐of‐principle, voltammetric anion sensing studies were carried out for the three rotaxane receptors with HSO_4_^−^, Cl^−^ and OAc^−^ in CH_2_Cl_2_. In all cases quantitative analysis was carried out only for the P/P^+.^ couple. Titrations were conducted at constant ionic strength (100 mM) and monitored by square‐wave voltammetry (SWV).

**Figure 10:**
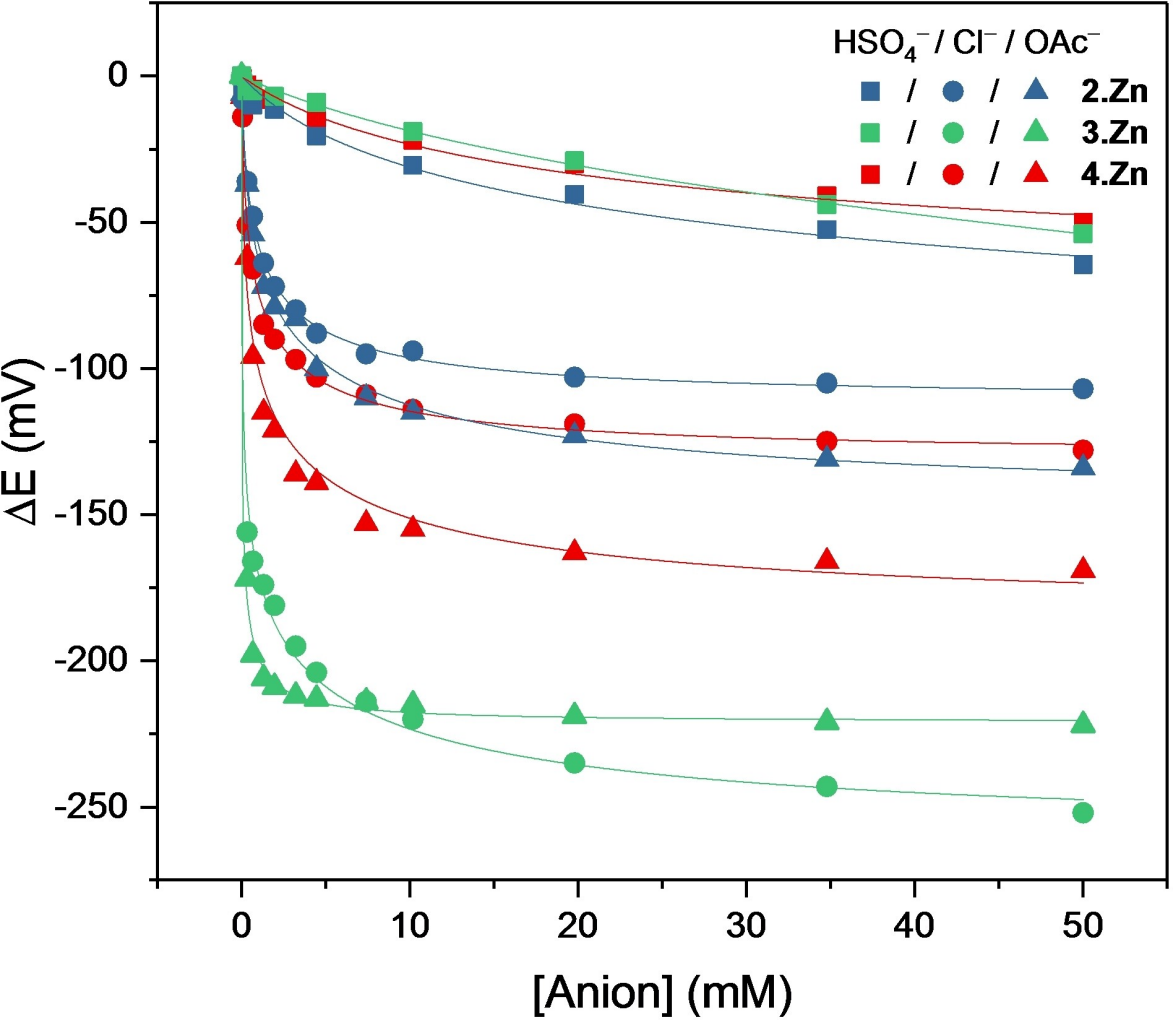
Cathodic voltammetric shifts of **2**⋅Zn (blue), **3**⋅Zn (green) and **4**⋅Zn (red) upon titration with HSO_4_^−^ (squares), Cl^−^ (circles) and OAc^−^ (triangles) in CH_2_Cl_2_, 100 mM TBAPF_6_. Solid lines represent fits to the 1 : 1 Nernst model.^[5g]^ The sensor response of each rotaxane separately is also shown in Figures S8‐11 to −13.

As shown in Figures 10 and S8‐11 to −13, all three anions induced significant cathodic perturbations for **2**⋅Zn, in particular Cl^−^ and OAc^−^ (see also Table 5). Rotaxane **4**⋅Zn displayed the same response trends: OAc^−^>Cl^−^>HSO_4_^−^, with a significantly enhanced response towards the first two anions. **3**⋅Zn responded even more strongly to OAc^−^ and Cl^−^ with large maximum cathodic shifts of −222 and −252 mV, respectively. In particular at high anion concentrations Cl^−^ now induced a larger response of up to −252 mV. A 1 : 1 host–guest binding stoichiometry was further indicated by fitting of the voltammetric isotherms to a Nernst binding model,[^5g^, ^22^] affording good fits in all cases. From this, anion binding association constants to the different receptor oxidation states (*K*_ox_ and *K*_red_) were also determined and are tabulated in Table 5. As expected, *K*_ox_ was significantly larger than *K*_red_ (often by multiple orders of magnitude), indicative of strong anion binding switch‐on upon receptor oxidation. Overall, this highlights the ability of these XB rotaxane receptors to sense anions electrochemically through significant, large cathodic shifts of the respective rotaxane's porphyrin P/P^+.^ half‐wave potential.

**Table 5:** Maximum cathodic shifts Δ*E*_Max_ [mV] of the P/P^+.^ couple of **2**–**4**⋅Zn upon titration with anions (at [anion]=50 mM) and anion binding constants to the oxidised (*K*_ox_) and native receptor state (*K*_red_) in CH_2_Cl_2_, 100 mM TBAPF_6_, obtained by fitting of the voltammetric isotherms according to a Nernst binding model Equation (1) (see the Supporting Information). Estimated error≤5 mV.

*E*_1/2_ in CH_2_Cl_2_/TBAPF_6_	Δ*E*_max_ [mV]	*K*_ox_ [M^−1^]	*K*_red_ [M^−1^]
**2**⋅Zn+HSO_4_^−^	−65	254	5
**2**⋅Zn+Cl^−^	−107	9 700	128
**2**⋅Zn+OAc^−^	−134	10 900	36
**3**⋅Zn+HSO4^−^	−54	97	0
**3**⋅Zn+Cl^−^	−252	80 000	31
**3**⋅Zn+OAc^−^	−222	4010000	718
**4**⋅Zn+HSO_4_^−^	−50	175	11
**4**⋅Zn+Cl^−^	−128	19 400	122
**4**⋅Zn+OAc^−^	−169	50 500	38

## Conclusion

In summary, two novel XB bis(iodotriazole) strapped zinc(II) porphyrins **1 a**⋅Zn and **1 b**⋅Zn were prepared, and their anion recognition properties were investigated by UV/visible titration. Interestingly, strong halide binding was observed for the shorter‐strap **1 a**⋅Zn porphyrin in acetone whereas intramolecular triazole coordination to the zinc(II) centre completely negated anion affinity in the longer‐strap analogue **1 b**⋅Zn. Using the CuAAC‐AMT strategy, **1 a**⋅Zn was exploited to construct the first examples of XB porphyrin‐BODIPY [2]rotaxanes **3**⋅Zn and **4**⋅Zn, which are capable of functioning as dual‐signalling chemosensors – detecting anions through changes in both fluorescent optical and redox electrochemical properties. In these interlocked systems, intercomponent interactions between the axle triazole motif and zinc(II) metal centre of the macrocyclic porphyrin substantially enhance the preorganisation of binding sites and strengthen their anion affinities. Fluorescence titrations conducted in acetone and H_2_O/acetone (2 : 98 *v*/*v*) demonstrated the ability of the rotaxanes to optically sense anions by quenching the respective rotaxane axle BODIPY‐centred emission. The relatively larger magnitudes of anion recognition‐induced quenching and stronger anion binding observed for **4**⋅Zn were attributed to the electron‐withdrawing perfluoroaryl group covalently linked to the BODIPY fluorophore and axle triazole. Voltammetric investigations revealed that the XB rotaxane receptors **3**⋅Zn and **4**⋅Zn sense anions through significant, large‐magnitude cathodic perturbations of their respective porphyrin P/P^+.^ oxidation couples. This demonstrates the unique MIM synthetic design versatility of integrating complementary optical and electrochemical motifs into the macrocycle and axle components of a XB rotaxane host for developing potential dual transducer anion sensory systems.

## Additional Information

Deposition Number 2095235 (for **1 a**⋅Zn) contains the supplementary crystallographic data for this paper. These data are provided free of charge by the joint Cambridge Crystallographic Data Centre and Fachinformationszentrum Karlsruhe Access Structures service.

## Conflict of interest

The authors declare no conflict of interest.

## Supporting information

As a service to our authors and readers, this journal provides supporting information supplied by the authors. Such materials are peer reviewed and may be re‐organized for online delivery, but are not copy‐edited or typeset. Technical support issues arising from supporting information (other than missing files) should be addressed to the authors.

Supporting Information
