# Supplementary material for: Halogen‐Bonding Strapped Porphyrin BODIPY Rotaxanes for Dual Optical and Electrochemical Anion Sensing
Source: Chemistry. 2021 Sep 6;27(58):14550–9. doi: 10.1002/chem.202102493 (PMC8596797; doi:10.1002/chem.202102493)
Supplement: Supplementary file 1 — Supporting Information [file CHEM-27-14550-s001.pdf]

Chemistry–A European Journal

Supporting Information

Halogen-Bonding Strapped Porphyrin BODIPY Rotaxanes for Dual Optical and Electrochemical Anion Sensing

Yuen Cheong Tse, Robert Hein, Edward J. Mitchell, Zongyao Zhang, and Paul D. Beer*

Table of Contents

S1 Synthesis and Characterisation 1

S2 Spectral Characterisation of Novel Compounds 22

S3 Single Crystal X-Ray Diffraction Studies 42

S4 UV-Visible Absorption Studies 45

S5 ^{1}H NMR Anion Binding Studies 48

S6 UV-Visible Anion Binding Studies 54

S7 Fluorescence Anion Binding Studies 57

S8 Electrochemical Studies 64

S9 References 73

S1 Synthesis and Characterisation

General Procedures

All solvents and reagents were purchased from commercial suppliers and used as received unless otherwise stated. Dry solvents were obtained by purging with nitrogen and then passing through an MBraun MPSP-800 column. H_2O was de-ionized and micro filtered using a Milli-Q ® Millipore machine. Column chromatography was carried out on Merck® silica gel 60 under a positive pressure of nitrogen. Routine NMR spectra were recorded on either a Varian Mercury 300, a Bruker AVIII 400 or a Bruker AVIII 500 spectrometer with ^{1}H NMR titrations recorded on a Bruker AVIII 500 spectrometer. TBA salts were stored in a vacuum desiccator containing phosphorus pentoxide prior to use. Where mixtures of solvents were used, ratios are reported by volume. Chemical shifts are quoted in parts per million relative to the residual solvent peak. Mass spectra were recorded on a Bruker µTOF spectrometer. Triethylamine was distilled from and stored over potassium hydroxide.

Tris[(1-benzyl-1H-1,2,3-triazol-4-yl)methyl]amine (TBTA)[1] was synthesised according to a reported procedure.[1]

Synthetic Procedures and Characterisation

Synthesis of XB Strapped Porphyrins

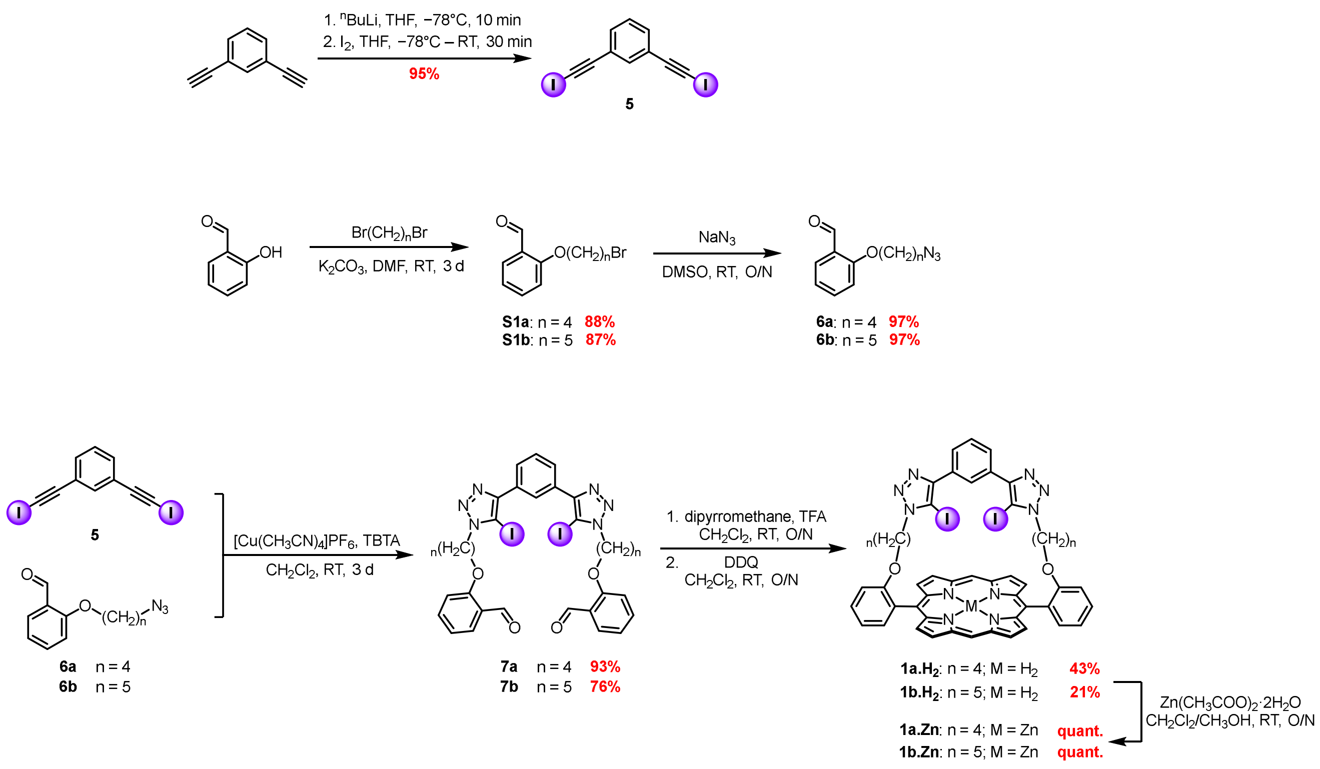

Scheme S1-1. Synthetic route of strapped porphyrin macrocycles **1a–b.Zn**.

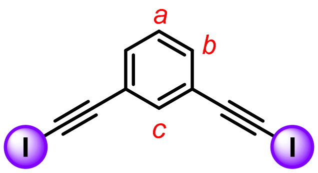

5

According to a reported procedure,[2] 1,3-bis(ethynyl)benzene (1.00 g, 7.93 mmol, 1 equiv) was dissolved in dry THF (25 mL) and the solution was cooled to –78°C. 2.5 M *n*-Butyl lithium in *n*-hexane (15.9 mL, 39.6 mmol, 5 equiv) was added slowly at –78°C and the reaction mixture was left to stir for 10 minutes. A solution of I_2 (10.1 g, 39.6 mmol, 5 equiv) in dry THF (30 mL) was added to the reaction mixture slowly and the reaction mixture was allowed to warm back to room temperature. H_2O (30 mL) and saturated $Na_2S_2O_{3\ (aq.)}$ (30 mL) were added and the product was extracted with $CHCl_3$ (50 mL × 3). The combined organic layer was washed with brine (20 mL), dried over anhydrous $MgSO_4$, filtered and concentrated on rotary evaporator. Further evaporation with silica gel column chromatography (petroleum ether 40–60°C boiling point) afforded the product as off-white solid (2.46 g, 82%).

^{1}H NMR (400 MHz, $CDCl_3$) δ (ppm): 7.50 (t, J = 1.7 Hz, 1H; *c*), 7.38 (dd, J = 7.8, 1.7 Hz, 2H; *b*), 7.26 (t, J = 7.8 Hz, 1H; *a*).

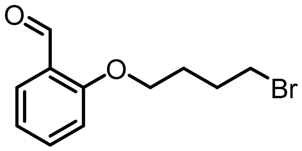

S1a

According to a reported procedure,[3] salicylaldehyde (3.00 g, 24.6 mmol, 1 equiv) and anhydrous K_2CO_3 (5.09 g, 36.8 mmol, 1.5 equiv) were added to dry DMF (25 mL). The mixture was stirred at room temperature for 10 minutes. 1,4-Dibromobutane (23.5 mL, 197 mmol, 8 equiv) was added and the reaction mixture was stirred at room temperature for 3 days. The solvent was removed *in vacuo* and the crude was purified by silica gel column chromatography (5% EtOAc in *n*-hexane) to afford the **S1a** as colourless oil (5.56 g, 88%).

^{1}H NMR (400 MHz, $CDCl_3$) δ (ppm): 10.50 (d, J = 0.8 Hz, 1H, CHO), 7.84 (dd, J = 7.7, 1.9 Hz, 1H; Aryl H), 7.54 (ddd, J = 8.4, 7.3, 1.8 Hz, 1H; Aryl H), 7.03 (t, J = 7.7 Hz, 1H; Aryl H), 6.97 (d, J = 8.5 Hz, 1H; Aryl H), 4.13 (t, J = 6.0 Hz, 2H; CH_2), 3.51 (t, J = 6.5 Hz, 2H; CH_2), 2.20 – 1.91 (m, 4H; CH_2).

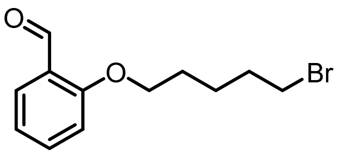

S1b

According to a modified reported procedure,[4] salicylaldehyde (3.00 g, 24.6 mmol, 1 equiv) and anhydrous K_2CO_3 (5.09 g, 36.8 mmol, 1.5 equiv) were added to dry DMF (25 mL). The mixture was stirred at room temperature for 10 minutes. 1,5-Dibromobutane (26.8 mL, 197 mmol, 8 equiv) was added and the reaction mixture was stirred at room temperature for 3 days. The solvent was removed *in vacuo* and the crude was purified by silica gel column chromatography (5% EtOAc in *n*-hexane) to afford the **S1a** as colourless oil (6.07 g, 87%).

^{1}H NMR (400 MHz, $CDCl_3$) δ(ppm): 10.51 (d, J = 0.8 Hz, 1H; CHO), 7.84 (dd, J = 7.7, 1.9 Hz, 1H; Aryl H), 7.53 (ddd, J = 8.4, 7.3, 1.9 Hz, 1H; Aryl H), 7.02 (tt, J = 7.5, 0.9 Hz, 1H; Aryl H), 6.97 (dd, J = 8.5, 0.9 Hz, 1H; Aryl H), 4.10 (t, J = 6.3 Hz, 2H; CH_2), 3.45 (t, J = 6.7 Hz, 2H; CH_2), 2.01 – 1.93 (m, 2H; CH_2), 1.93 – 1.85 (m, 2H; CH_2), 1.74 – 1.61 (m, 2H; CH_2).

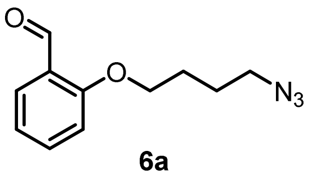

6a

According to a reported procedure,[3] bromide **S1a** (5.56 g, 21.6 mmol, 1 equiv) was dissolved in DMSO (50 mL). NaN_3 (1.69 g, 25.9 mmol, 1.2 equiv) was added and the reaction mixture was stirred at room temperature overnight. The crude was diluted with H_2O (100 mL) and extracted with diethyl ether (50 mL × 3). The combined organic layer was washed with brine (50 mL × 3), dried over anhydrous $MgSO_4$, filtered and concentrated on rotary evaporator to give the azide **6a** as yellow oil (4.62 g, 97%).

^{1}H NMR (400 MHz, $CDCl_3$) δ(ppm): 10.50 (d, J = 0.8 Hz, 1H, CHO), 7.84 (dd, J = 7.7, 1.9 Hz, 1H, ArH), 7.54 (ddd, J = 8.4, 7.3, 1.8 Hz, 1H, ArH), 7.03 (t, J = 7.7 Hz, 1H, ArH), 6.97 (d, J = 8.5 Hz, 1H, ArH), 4.12 (t, J = 6.0 Hz, 2H, CH_2), 3.39 (t, J = 6.5 Hz, 2H, CH_2), 2.02 – 1.90 (m, 2H, CH_2), 1.90 – 1.77 (m, 2H, CH_2).

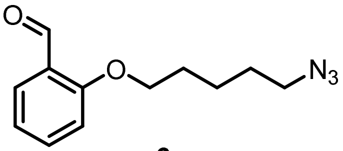

6a

According to a reported procedure,[5] bromide **S1b** (6.07 g, 21.3 mmol, 1 equiv) was dissolved in DMSO (60 mL). NaN_3 (1.66 g, 25.5 mmol, 1.2 equiv) was added and the reaction mixture was stirred at room temperature overnight. The crude was diluted with H_2O (100 mL) and extracted with diethyl ether (50 mL × 3). The combined organic layer was washed with brine (50 mL × 3), dried over anhydrous $MgSO_4$, filtered and concentrated on rotary evaporator to give the azide **6a** as yellow oil (5.11 g, 97%).

^{1}H NMR (400 MHz, $CDCl_3$) δ(ppm): 10.51 (d, J = 0.8 Hz, 1H; CHO), 7.84 (dd, J = 7.7, 1.8 Hz, 1H; Aryl H), 7.54 (ddd, J = 8.4, 7.3, 1.9 Hz, 1H; Aryl H), 7.02 (tt, J = 7.4, 0.9 Hz, 1H; Aryl H), 6.97 (d, J = 8.4 Hz, 1H; Aryl H), 4.10 (t, J = 6.3 Hz, 2H; CH_2), 3.33 (t, J = 6.7 Hz, 2H; CH_2), 1.96 – 1.84 (m, 2H; CH_2), 1.76 – 1.66 (m, 2H; CH_2), 1.66 – 1.57 (m, 2H; CH_2).

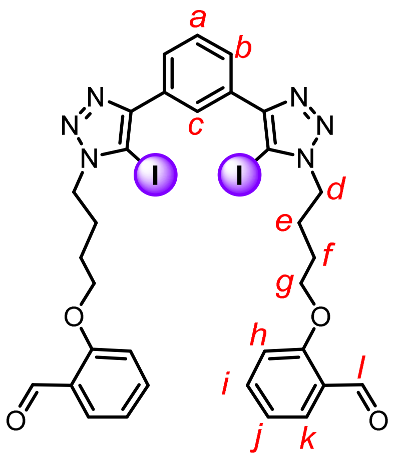

7a

General Procedure for CuAAC Click Reaction

$[Cu(CH_3CN)_4]PF_6$ (99 mg, 0.26 mmol, 0.2 equiv) and TBTA (140 mg, 0.26 mmol, 0.2 equiv) were added to dry and degassed THF (7 mL) and the mixture was stirred at room temperature for 20 minutes until all solid dissolved, upon which a pale yellow solution was obtained. A solution of azide **6** (870 mg, 3.97 mmol, 3 equiv) in dry and degassed THF (2 mL) was added, followed by a slow addition of bis(iodoalkyne) **5** (500 mg, 1.32 mmol, 1 equiv) in dry and degassed THF (2 mL). The reaction mixture turned green immediately upon the addition of **5** and was allowed to stir at room temperature under N_2 in dark for 3 days. The crude mixture was diluted with $CHCl_3$ (50 mL) and washed with basic EDTA (20 mL x 2) and brine (20 mL). The solvent was removed *in vacuo* and further purification with silica gel column chromatography (neat CH_2Cl_2 gradient to 20% EtOAc in CH_2Cl_2) gave the product as white foam (1.01 g, 93%).

^{1}H NMR (400 MHz, $CDCl_3$) δ (ppm): 10.52 (d, J = 0.8 Hz, 2H; *l*), 8.55 (t, J = 1.8 Hz, 1H; *c*), 7.99 (dd, J = 7.8, 1.8 Hz, 2H; *b*), 7.83 (dd, J = 7.6, 1.9 Hz, 2H; *k*), 7.58 (t, J = 7.8 Hz, 1H; *a*), 7.53 (ddd, J = 8.4, 7.4, 1.9 Hz, 2H; *i*), 7.02 (t, J = 7.6 Hz, 2H; *j*), 6.97 (d, J = 8.4 Hz, 2H; *h*), 4.59 (t, J = 7.0 Hz, 4H; *d*), 4.15 (t, J = 6.0 Hz, 4H; *g*), 2.31 – 2.19 (q, J = 6.0 Hz, 4H; *f*), 2.04 – 1.92 (q, J = 7.0Hz, 4H; *e*).

^{13}C NMR (126 MHz, $CDCl_3$) δ (ppm): 189.82, 161.15, 149.71, 136.26, 130.60, 129.20, 128.62, 127.82, 125.99, 124.79, 120.97, 112.40, 76.65, 67.34, 50.57, 26.61, 26.09.

HRMS (ESI +ve) m/z: 817.04841 ($[M+H]^+$, $C_{32}H_{31}O_4N_6I_2$ requires 817.04907).

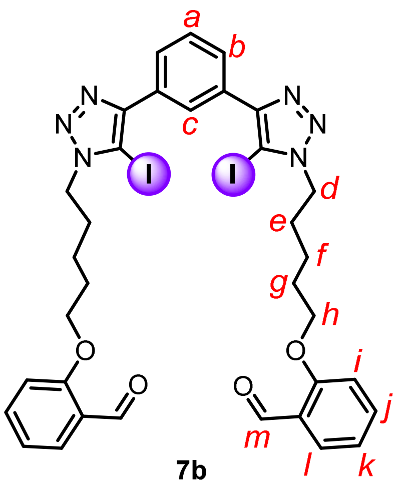

7b

Following the general procedure for CuAAC click reaction between bis-iodoalkyne **5** (500 mg, 1.32 mmol, 1 equiv) and azide **6b** (981 mg, 3.97 mmol, 3 equiv), purification by silica gel column chromatography (20% EtOAc in CH_2Cl_2) afforded compound **7b** as white powder (0.85 g, 76%).

^{1}H NMR (400 MHz, $CDCl_3$) δ (ppm): 10.51 (d, *J* = 0.9 Hz, 2H; *m*), 8.55 (t, *J* = 1.8 Hz, 2H; *c*), 8.00 (dd, *J* = 7.8, 1.7 Hz, 2H; *b*), 7.83 (ddd, *J* = 7.7, 1.9, 0.9 Hz, 2H; *l*), 7.58 (t, *J* = 7.8 Hz, 2H; *a*), 7.53 (d, *J* = 7.9 Hz, 2H; *j*), 7.02 (td, *J* = 7.5, 1.0 Hz, 2H; *k*), 6.96 (d, *J* = 8.4 Hz, 2H; *i*), 4.51 (t, *J* = 7.2 Hz, 4H; *d*), 4.10 (t, *J* = 6.3 Hz, 4H; *h*), 2.09 (q, *J* = 7.2 Hz, 4H; *e*), 1.95 (q, *J* = 6.3 Hz, 4H; *g*), 1.64 (p, *J* = 8.2 Hz, 4H; *f*).

^{13}C NMR (126 MHz, $CDCl_3$) δ (ppm): 189.97, 161.38, 149.56, 136.20, 130.70, 129.11, 128.48, 127.76, 126.24, 124.84, 120.81, 112.49, 76.77, 68.03, 50.81, 29.72, 28.68, 23.21.

HRMS (ESI +ve) m/z: 845.07991 ($[M+H]^+$, $C_{34}H_{35}O_4N_6I_2$ requires 845.08037).

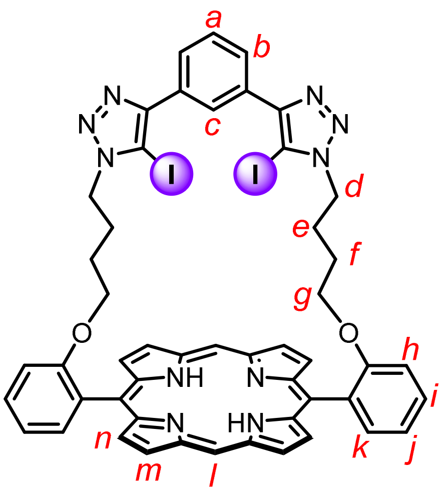

1a.H₂

General Procedure for Porphyrin Formation by Pyrrole-Aldehyde Condensation Reaction

Dialdehyde **7a** (400 mg, 0.49 mmol, 1 equiv) and dipyrromethane (143 mg, 0.98 mmol, 2 equiv) were dissolved in dry and degassed CH_2Cl_2 (100 mL). Trifluoroacetic acid (TFA) (28 μL, 0.30 mmol, 0.6 equiv) was added and the reaction mixture was stirred at room temperature under N_2 overnight. DDQ (167 mg, 0.74 mmol, 1.5 equiv) was added and the mixture was stirred at room temperature overnight. Et_3N (69 μL, 0.49 mmol, 1 equiv) was added and the crude was filtered thorugh filter paper to remove black polymeric side products. The filtrate was washed with saturated aqueous $NaHCO_3$ (50 mL × 3), dried over anhydrous $MgSO_4$, filtered and concentrated *in vacuo*. Further purification with silica gel column chromatography (10% EtOAc in CH_2Cl_2) gave the product **1a.H$_2$** as purple powder (240 mg, 43%).

^{1}H NMR (500 MHz, $CDCl_3$) δ (ppm): 10.04 (s, 2H; *l*), 9.22 (d, J = 4.5 Hz, 4H; *m*), 8.97 (d, J = 4.5 Hz, 4H; *n*), 7.90 (dd, J = 7.7, 1.7 Hz, 2H; *b*), 7.77 (ddd, J = 8.8, 7.3, 1.7 Hz, 2H; *i*), 7.62 (dd, J = 7.3, 1.7 Hz, 2H; *k*), 7.51 (t, J = 7.7 Hz, 1H; *a*), 7.45 (d, J = 8.8 Hz, 2H; *h*), 7.29 (t, J = 7.3 Hz, 2H; *j*), 7.17 (t, J = 1.7 Hz, 1H; *c*), 4.27 (t, J = 5.9 Hz, 4H; *d*), 4.05 (t, J = 7.2 Hz, 4H; *g*), 1.72 (q, J = 7.2Hz, 4H; *f*), 1.48 (q, J = 5.9Hz, 4H; *e*), –3.09 (s, 2H; pyrrole NH).

^{13}C NMR (126 MHz, $CDCl_3$) δ (ppm): 157.76, 149.08, 147.60, 145.15, 137.67, 131.66, 131.30, 130.12, 130.01, 129.95, 129.53, 127.78, 124.05, 119.91, 115.74, 111.74, 104.97, 67.80, 50.24, 29.85, 26.52, 25.30.

HRMS (ESI +ve) m/z: 1067.14824 ([M+H]$^+$, $C_{50}H_{41}O_2N_{10}I_2$ requires 1067.14978).

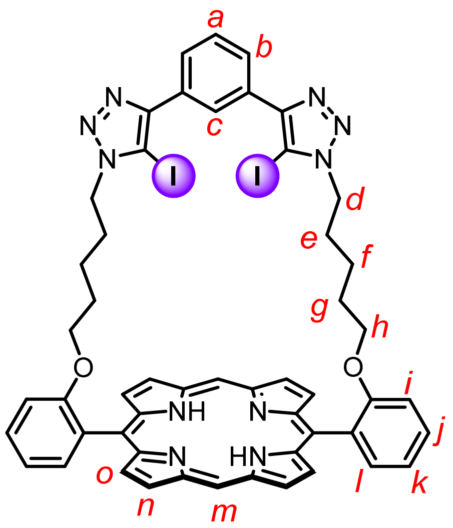

1b.H$_2$

Following the general procedure for porphyrin formation by pyrrole-aldehyde condensation reaction between bis-aldehyde **7b** (500 mg, 0.59 mmol, 1 equiv) and dipyrromethene (173 mg, 1.18 mmol, 2 equiv), purification by silica gel column chromatography (10% EtOAc in CH_2Cl_2) afforded strapped porphyrin **1b.H$_2$** as purple powder (136 mg, 21%).

^{1}H NMR (500 MHz, $CDCl_3$) δ (ppm): 10.21 (s, 2H; *m*), 9.33 (d, *J* = 4.5 Hz, 4H; *n*), 9.02 (d, *J* = 4.5 Hz, 4H; *o*), 7.78 (m, 4H; *b*, j), 7.71 (dd, *J* = 7.6, 1.8 Hz, 2H; *l*), 7.42 (d, *J* = 7.7 Hz, 2H; *i*), 7.40 (d, *J* = 7.2 Hz, 1H; *a*), 7.32 (t, *J* = 7.4 Hz, 2H; *k*), 7.29 (d, *J* = 1.9 Hz, 1H; *c*), 4.06 (t, *J* = 6.9 Hz, 4H; *d*), 3.90 (t, *J* = 6.5 Hz, 4H; *h*), 1.50 (p, *J* = 6.5 Hz, 4H; *g*), 1.33 (p, *J* = 6.9 Hz, 4H; *e*), 0.87 (p, *J* = 7.8 Hz, 4H; *f*).

^{13}C NMR (126 MHz, $CDCl_3$) δ (ppm): 158.06, 149.18, 147.61, 145.18, 136.87, 131.58, 131.30, 130.37, 130.08, 130.00, 129.42, 127.91, 125.09, 119.71, 115.87, 112.12, 104.92, 68.06, 50.03, 29.86, 29.30, 28.10, 22.73.

HRMS (ESI +ve) m/z: 1095.17706 ($[M+H]^+$, $C_{52}H_{45}O_2N_{10}I_2$ requires 1095.18108).

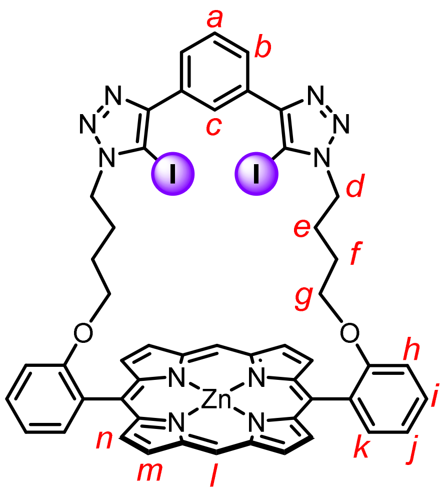

1a.Zn

General Procedure for Porphyrin Metalation Reaction

Free-base porphyrin **1a.H₂** (130 mg, 0.12 mmol, 1 equiv) was dissolved in 1:1 $CHCl_3$:CH_3OH (20 mL). $Zn(CH_3COO)_2$•$2H_2O$ (112 mg, 0.61 mmol, 5 equiv) was added and the reaction mixture was stirred at room temperature overnight. The organic solvent was removed *in vacuo* and the solid residue was redissolved in CH_2Cl_2 (50 mL) and washed with H_2O (30 mL × 3). The combined organic layer was dried over anhydrous $MgSO_4$, filtered and concentrated on rotary evaporator to give the product as shiny purple-red powder (138 mg, quant.).

^{1}H NMR (500 MHz, $CDCl_3$) δ (ppm): 10.01 (s, 2H; *l*), 9.24 (d, *J* = 4.5 Hz, 4H; *m*), 9.03 (d, *J* = 4.5 Hz, 4H; *n*), 7.98 (dd, *J* = 7.7, 1.7 Hz, 2H; *b*), 7.78 (ddd, *J* = 8.8, 7.3, 1.7 Hz, 2H; *i*), 7.67 (dd, *J* = 7.3, 1.7 Hz, 2H; *k*), 7.60 (t, *J* = 7.7 Hz, 1H; *a*), 7.46 (d, *J* = 8.8 Hz, 2H; *h*), 7.31 (t, *J* = 1.7 Hz, 1H, *c*), 7.29 (t, *J* = 7.3 Hz, 2H; *j*), 4.23 (t, *J* = 5.9 Hz, 4H; *d*), 4.05 (t, *J* = 7.2 Hz, 4H; *g*), 1.70 (m, 4H; *e*), 1.49 (m, 4H; *f*).

^{13}C NMR (126 MHz, $CDCl_3$) δ (ppm): 157.83, 150.53, 149.50, 149.23, 137.42, 132.63, 131.92, 131.38, 130.33, 129.76, 129.63, 127.88, 124.80, 119.71, 116.63, 111.68, 106.05, 67.81, 50.24, 29.86, 26.75, 25.37.

HRMS (ESI +ve) m/z: 1151.04192 ([M+Na]$^+$, $C_{50}H_{38}O_2N_{10}I_2NaZn$ requires 1151.04522).

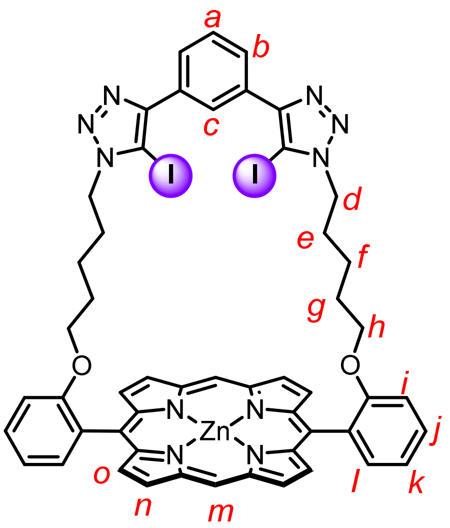

1b.Zn

Following the general procedure for porphyrin metalation reaction between free base porphyrin **1b.H$_2$** (132 mg, 0.12 mmol, 1 equiv) and Zn(CH_3COO)$_2$·2H_2O (111 mg, 0.60 mmol, 5 equiv), aqueous work-up afforded the product **1b.Zn** as purple powder (140 mg, quant.).

^{1}H NMR (500 MHz, $CDCl_3$) δ (ppm): 10.16 (s, 2H; *m*), 9.35 (d, *J* = 4.4 Hz, 4H; *n*), 9.04 (d, *J* = 4.4 Hz, 4H; *o*), 7.84 (dd, *J* = 7.2, 1.7 Hz, 2H; *b*), 7.76 (ddd, *J* = 9.1, 7.6, 1.8 Hz, 2H; *j*), 7.46 (dd, *J* = 8.0, 1.7 Hz, 2H; *l*), 7.42 – 7.37 (m, 3H; *a, i*), 7.31 (td, *J* = 7.4, 1.1 Hz, 2H; *k*), 6.95 (t, *J* = 1.6 Hz, 1H; *c*), 4.01 (t, *J* = 6.3 Hz, 4H; *d*), 3.91 (t, *J* = 6.7 Hz, 4H; *h*), 1.50 (p, *J* = 6.7 Hz, 2H; *g*), 1.30 – 1.26 (m, 4H; *e*), 0.80 (p, *J* = 7.7 Hz, 4H; *f*).

^{13}C NMR (126 MHz, $CDCl_3$) δ (ppm): 158.21, 150.45, 149.48, 149.40, 136.75, 132.36, 131.98, 131.74, 129.74, 129.60, 129.30, 128.07, 126.02, 119.54, 116.44, 112.09, 105.96, 67.98, 50.14, 29.86, 28.99, 28.07, 22.31.

HRMS (ESI +ve) m/z: 1157.09234 ([M+H]$^+$, $C_{52}H_{43}O_2N_{10}I_2Zn$ requires 1157.09458).

Synthesis of BODIPY-functionalised Stoppers

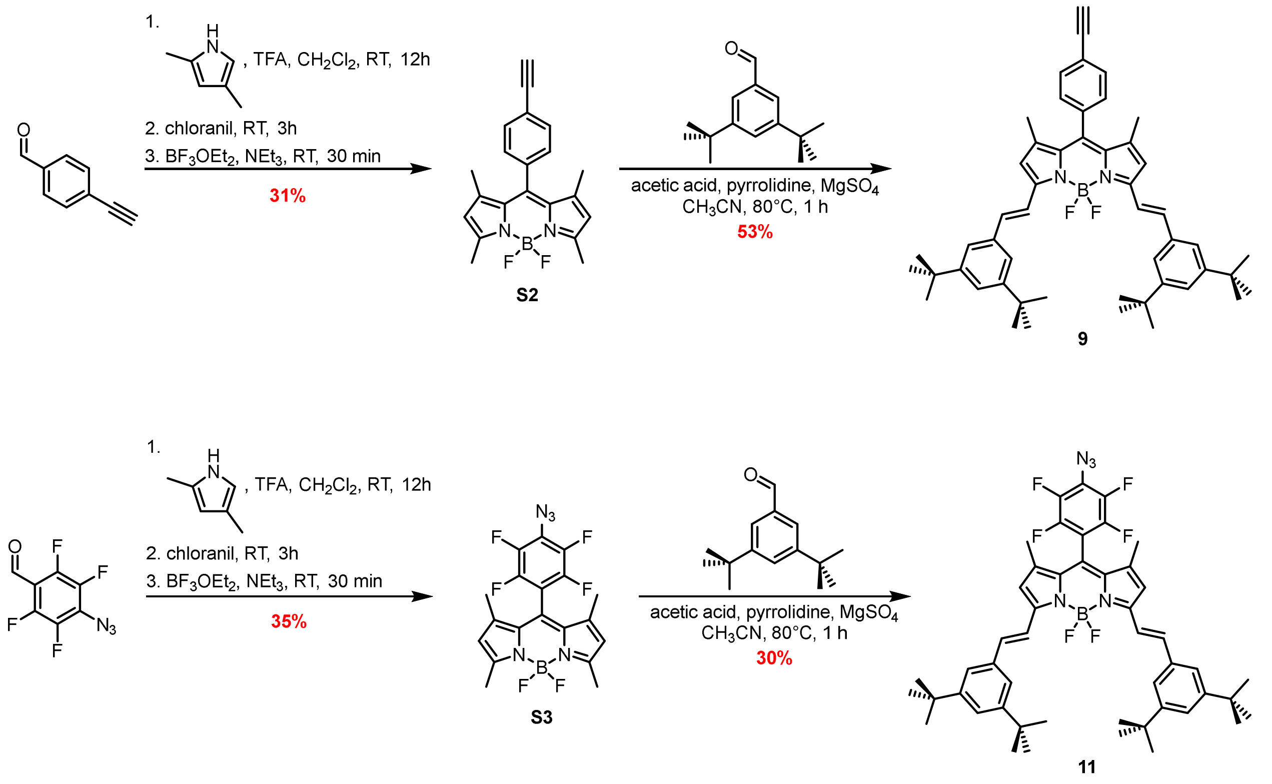

Scheme S1-2. Synthesis of BODIPY-functionalised stoppers alkyne **9** and azide **11**.

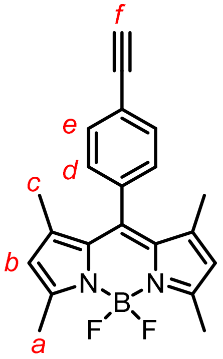

S2

General Procedure for BODIPY Synthesis

According to a reported procedure,[6] 4-ethynylbenzaldehyde (100 mg, 0.77 mmol, 1 equiv) and 2,4-dimethypyrrole (168 mg, 1.77 mmol, 2.3 equiv) were dissolved in dry degassed CH_2Cl_2 (50 mL) at RT. A catalytic amount of TFA (10 µL) was added and the reaction left to stir for 16 hours. *p*-Chloranil (208 mg, 0.85 mmol, 1.1 equiv) was then added and the reaction left to stir at RT for 2 hours. Distilled NEt_3 (0.26 mL, 1.9 mmol, 2.4 equiv) and $BF_3 \cdot OEt_2$ (0.26 mL, 2.1 mmol, 2.7 equiv) were added sequentially and the mixture left to stir for 30 minutes. The solution was then washed with H_2O (3 x 180 mL) and brine (180 mL) and dried over Na_2SO_4, filtered and the solvent removed *in vacuo*. The crude product was purified *via* column chromatography (petroleum ether (bp 40-60 °C):CH_2Cl_2 1:1 v/v) and the desired product was isolated as an orange-red solid (83 mg, 31%).

^{1}H NMR (400 MHz, $CDCl_3$) δ (ppm): 7.55 (d, J = 7.9 Hz, 2H; *e*), 7.20 (d, J = 7.9 Hz, 2H; *d*), 5.91 (s, 2H; *b*), 3.11 (s, 1H; *f*), 2.48 (s, 6H; *a*), 1.33 (s, 6H; *c*).

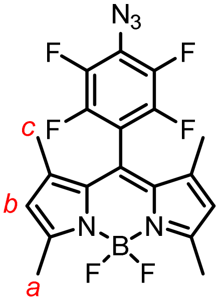

S3

Following general procedure for BODIPY synthesis using 2,4-dimethylpyrrole (472 µL, 4.68 mmol, 2.05 equiv) and 4-azido-tetrafluorobenzaldehyde (500 mg, 2.28 mmol, 1 equiv), crude was purified by silica gel column chromatography (CH_2Cl_2:*n*-hexane 2:3 v/v) to afford the product as a red solid (356 mg, 35%).

^{1}H NMR (400 MHz, $CDCl_3$) δ (ppm): 6.05 (s, 2H; *b*), 2.57 (s, 6H; *a*), 1.64 (s, 6H; *c*).

^{13}C NMR (151 MHz, $CDCl_3$) δ (ppm): 157.78, 144.04 (dm, $^1J_{CF}$ = 252 Hz, 2C), 141.73, 141.18 (dm, $^1J_{CF}$ = 252 Hz, 2C), 131.21, 123.44, 122.30, 32.08, 29.85, 14.94, 13.81

^{19}F NMR (376 MHz, $CDCl_3$) δ (ppm): -139.77 – -139.91 (m), -146.16 (q, *J* = 32.2 Hz), -149.95 – -150.14 (m).

HRMS (ESI +ve) m/z: 438.1317 ($[M+H]^+$, $C_{19}H_{15}N_5B_1F_6$ requires 438.1321).

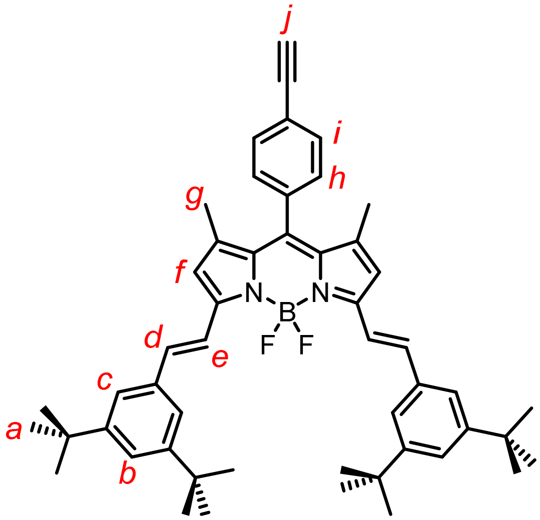

9

General Procedure for Knoevenagel Condensation

To a mixture of **S2** (100 mg, 0.23 mmol, 1 equiv) in CH_3CN (10 mL), $MgSO_4$ (275 mg, 2.3 mmol, 10 equiv), 3,5-di-*tert*-butylbenzaldehyde (200 mg, 0.91 mmol, 4 equiv), pyrrolidine (163 mg, 2.3 mmol, 10 eq) and glacial acetic acid (132 µL, 2.3 mmol, 10 equiv) were added. The reaction was immersed in an oil bath at 60°C, upon which a colour change from orange to blue was observed, for 1 h. The solvent was removed *in vacuo* and the crude dissolved in CH_2Cl_2 (10 mL) and washed with 0.05 M HCl (10 mL), saturated $NaHCO_3$ solution (10 mL) and brine (10 mL). The combined organics were dried over $MgSO_4$, filtered and the solvents removed *in vacuo*. Purification was achieved by column chromatography (eluent: CH_2Cl_2:*n*-hexane 3:2 v/v) to afford a blue powder (58 mg, 30%).

^{1}H NMR (400 MHz, $CDCl_3$) δ (ppm): 7.77 (d, J = 16.3 Hz, 2H; *e*), 7.65 (d, J = 8.3 Hz; *i*), 7.45 (d, J = 1.8 Hz, 4H; *c*), 7.41 (t, J = 1.8 Hz, 2H; *b*), 7.35 (d, J = 8.3 Hz, 2H; *h*), 7.29 (d, J = 16.3 Hz, 2H; *d*), 6.65 (s, 2H; *f*), 3.19 (s, 1H; *j*), 1.48 (s, 6H; *g*), 1.37 (s, 36H; *a*).

^{13}C NMR (101 MHz, $CDCl_3$) δ (ppm): 153.03, 151.25, 141.84, 137.44, 136.01, 135.96, 133.02, 132.93, 128.83, 123.71, 122.93, 121.95, 118.98, 117.87, 83.10, 78.68, 35.01, 31.55, 22.82, 15.04.

^{19}F NMR (376 MHz, $CDCl_3$) δ (ppm): -138.56 (q, J = 32.1 Hz).

HRMS (ESI +ve) m/z: 748.4748 ($[M+H]^+$, $C_{51}H_{59}N_2B_1F_2$ requires 748.4752).

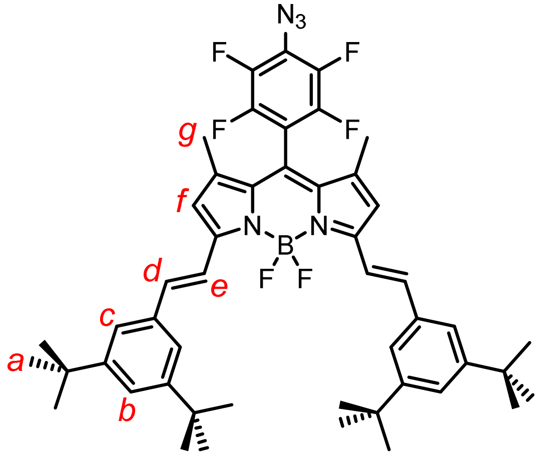

11

Following general procedure for Knoevenagel condensation with **S3** (100 mg, 0.23 mmol, 1 eq) and 3,5-di-*tert*-butylbenzaldehyde (200 mg, 0.91 mmol, 4 eq), product was obtained after silica gel column chromatography (CH_2Cl_2:*n*-hexane 3:2 v/v) as a blue powder (58 mg, 30%).

^{1}H NMR (400 MHz, $CDCl_3$) δ (ppm): 7.76 (d, J = 16.3 Hz, 2H; *d*), 7.48 – 7.41 (m, 6H; *b,c*), 7.33 (d, J = 16.3 Hz, 2H; *e*), 6.71 (s, 2H; *f*), 1.72 (s, 6H; *h*), 1.38 (s, 36H; *a*).

^{13}C NMR (126 MHz, $CDCl_3$) δ (ppm): 154.09, 151.27, 140.07, 138.59, 135.77, 132.99, 123.97, 122.01, 119.38, 118.73, 118.52, 34.90, 31.95, 31.42, 29.72, 29.38, 22.71, 14.14, 13.94.

^{19}F NMR (376 MHz, $CDCl_3$) δ (ppm): -138.57 (q, J = 32.3 Hz), -139.17 – -139.32 (m), -150.16 – -150.32 (m).

HRMS (ESI +ve) m/z: 838.4439 ($[M+H]^+$, $C_{49}H_{55}N_5B_1F_6$ requires 838.4457).

Synthesis of XB [2]Rotaxanes

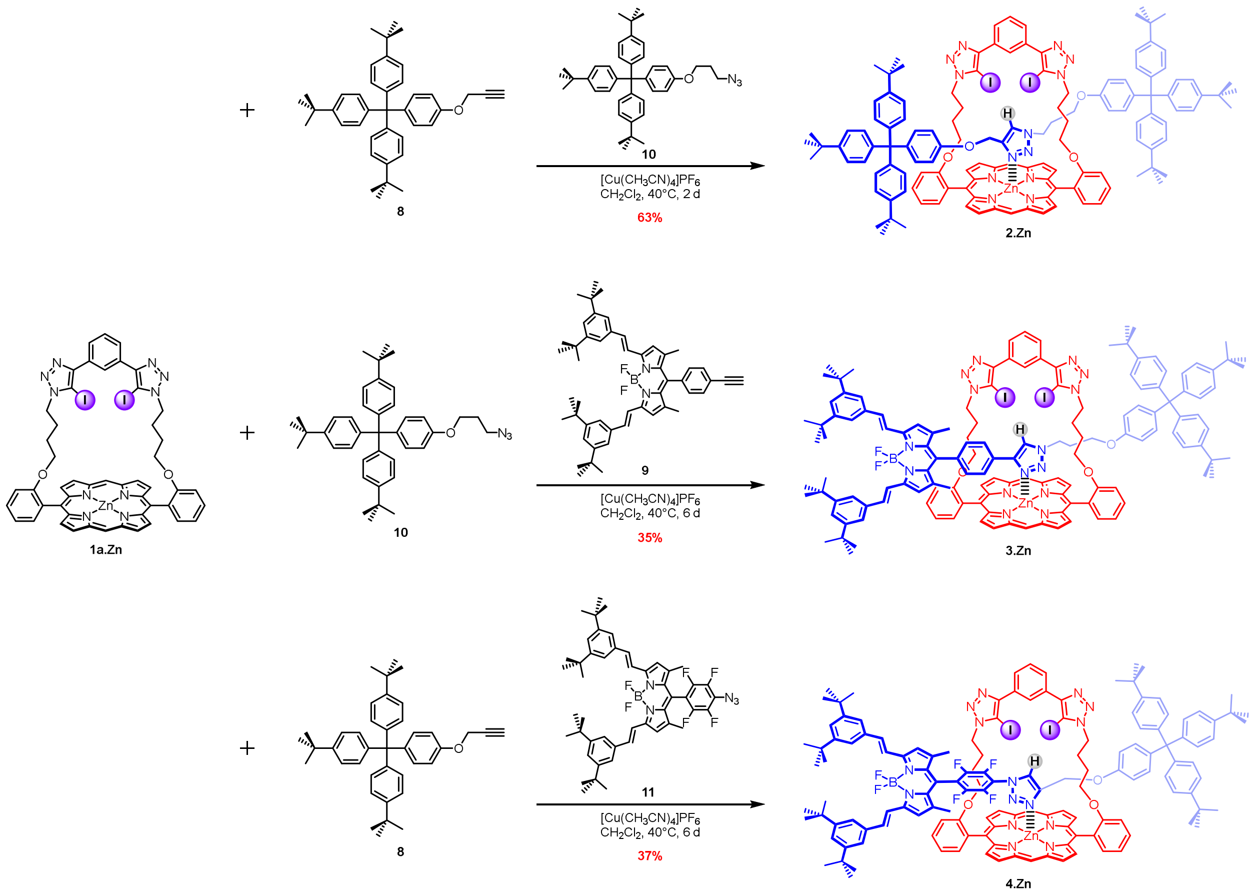

Scheme S1-3. Synthesis of neutral [2]rotaxanes **2–4.Zn** *via* CuAAC-AMT.

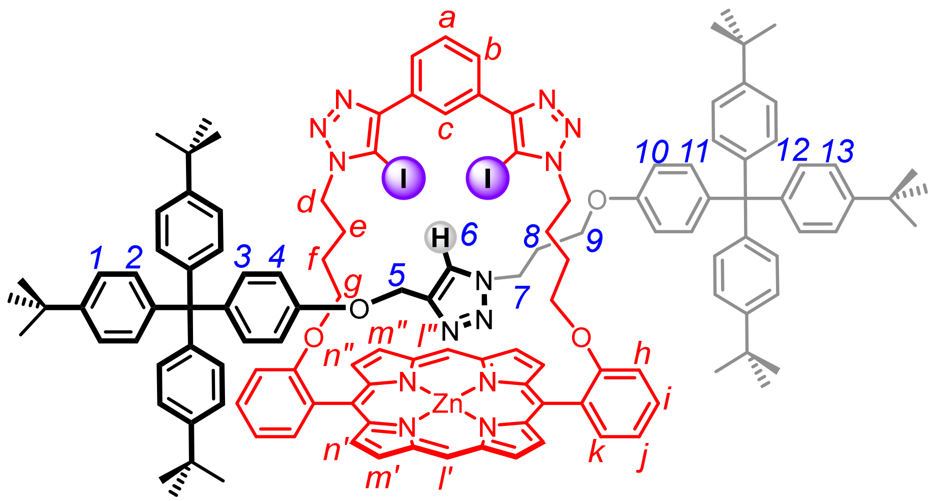

2.Zn

General Procedure for CuAAC-AMT Synthesis of [2]Rotaxanes

Strapped porphyrin **1a.Zn** (10 mg, 0.009 mmol, 1 equiv) and $[Cu(CH_3CN)_4]PF_6$ (3.3 mg, 0.009 mmol, 1 equiv) were dissolved in dry and degassed CH_2Cl_2 (0.8 mL) in a sealed vial and the mixture was stirred at room temperature under N_2 for 15 minutes. A solution of terphenyl-stoppered azide **10** (26 mg, 0.044 mmol, 5 equiv) in CH_2Cl_2 (0.5 mL) was added into the reaction vial, followed by addition of terphenyl-stoppered alkyne **8** (24 mg, 0.044 mmol, 5 equiv) dissolved in CH_2Cl_2 (0.5 mL). The reaction mixture was stirred at 40°C in dark for two days. The crude was diluted with CH_2Cl_2 (20 mL) and washed with basic aqueous EDTA (20 mL). The aqueous layer was back-extracted with CH_2Cl_2 (10 mL × 3) to minimise the loss of products. The combined organic layer was dried with anhydrous $MgSO_4$, filtered and concentrated on rotary evaporator. The crude solid residue obtained was purified by preparative TLC (2% EtOAc in CH_2Cl_2) to afford the product [2]rotaxane **2.Zn** as shiny red powder (13 mg, 63%).

^{1}H NMR (400 MHz, acetone-*d*$_6$) δ (ppm): 10.03 (s, 1H; *l'*), 9.63 (s, 1H, *l''*), 9.18 (d, *J* = 4.5 Hz, 2H; *m'*), 8.97 (d, *J* = 4.5 Hz, 2H; *m''*), 8.84 (d, *J* = 4.3 Hz, 2H; *n'*), 8.78 (d, *J* = 4.3 Hz, 2H; *n''*), 7.96 (dd, *J* = 7.3, 1.7 Hz, 2H; *b*), 7.70 (d, *J* = 8.2 Hz, 2H; *i*), 7.62 (dd, *J* = 7.7, 1.8 Hz, 2H; *k*), 7.59 (t, *J* = 1.8 Hz, 1H; *c*), 7.49 – 7.40 (m, 12H; *2,12*), 7.36 (t, *J* = 7.3 Hz, 1H; *a*), 7.31 (d, *J* = 5.6 Hz, 2H; *h*), 7.30 – 7.24 (m, 14H; *1,13,j*), 7.13 (d, *J* = 8.7 Hz, 2H; *3*), 6.89 (d, *J* = 8.7 Hz, 2H; *11*), 6.43 (d, *J* = 8.6 Hz, 2H; *4*), 6.24 (s, 1H; 6^{Trz}), 5.22 (d, *J* = 8.6 Hz, 2H; *10*), 4.22 – 4.04 (m, 4H; *d*), 3.65 – 3.43 (m, 4H; *g*), 2.92 (t, *J* = 6.2 Hz, 2H; *7*), 2.55 (t, *J* = 5.2 Hz, 2H; *9*), 1.58 – 1.49 (m, 4H; *f*), 1.06 (p, *J* = 6.2 Hz, 2H; *8*), 0.76 – 0.55 (m, 4H; *e*). (Signal of proton *5* missing due to peaks overlap).

^{13}**C NMR** (126 MHz, $CDCl_3$) δ (ppm): 158.91, 156.26, 154.72, 150.15, 150.06, 149.57, 149.02, 148.83, 148.51, 148.49, 144.52, 144.47, 139.71, 139.40, 139.35, 137.19, 132.59, 132.25, 131.83, 131.77, 131.67, 131.42, 131.32, 130.94, 130.90, 130.19, 129.19, 128.87, 127.20, 126.30, 124.37, 124.34, 121.70, 119.71, 115.31, 113.42, 113.08, 112.99, 105.55, 68.77, 63.28, 63.26, 62.99, 56.85, 50.18, 45.51, 34.55, 34.52, 31.66, 31.62, 25.67.

HRMS (ESI +ve) m/z: 2260.8035 ($[M+H]^+$, $C_{130}H_{134}I_2N_{13}O_4Zn$ requires 2260.8088)

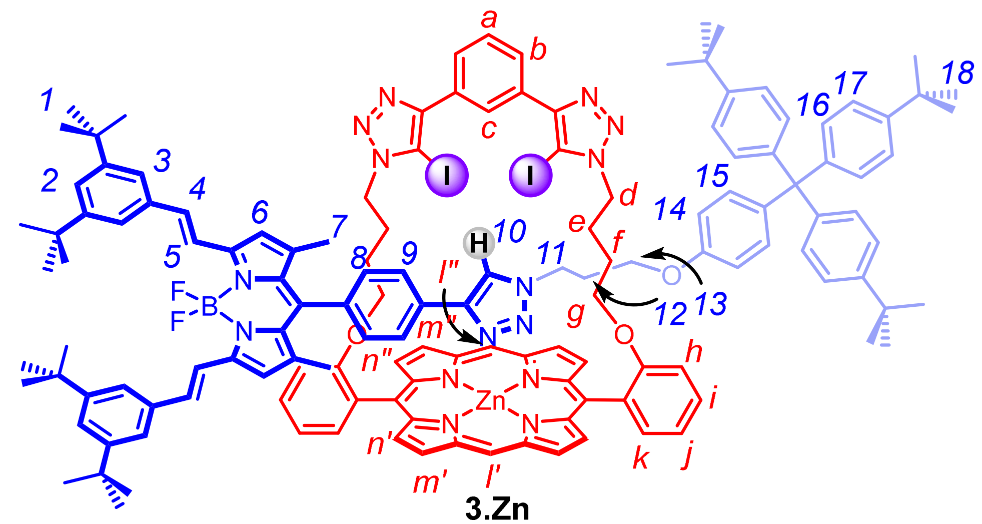

3.Zn

Following general procedure for CuAAC-AMT, strapped porphyrin **1a.Zn** (10 mg, 0.009 mmol, 1 equiv) was reacted with BODIPY-stoppered alkyne **9** (33 mg, 0.044 mmol, 5 equiv) and terphenyl-stoppered azide **10** (26 mg, 0.044 mmol, 5 equiv). The crude was purified by preparative TLC (4% EtOAc in CH_2Cl_2) to give the product [2]rotaxane **3.Zn** as blue powder (7.6 mg, 35%).

^{1}H NMR (500 MHz, $CDCl_3$) δ (ppm): 10.10 (s, 1H; *l'*), 9.77 (s, 1H; *l"*), 9.26 (d, *J* = 4.4 Hz, 2H; *m'*), 9.11 (d, *J* = 4.4 Hz, 2H; *m"*), 8.98 (d, *J* = 4.4 Hz, 2H; *n'*), 8.95 (d, *J* = 4.4 Hz, 2H; *n"*), 8.02 (dd, *J* = 7.3, 1.8 Hz, 2H; *b*), 7.80 (d, *J* = 16.2 Hz, 2H; *4*), 7.76 (t, *J* = 8.0 Hz, 2H; *i*), 7.56 (dd, *J* = 7.7, 1.8 Hz, 2H; *k*), 7.47 (d, *J* = 1.8 Hz, 4H; *3*), 7.42 (t, *J* = 1.8 Hz, 2H; *2*), 7.38 (t, *J* = 7.6 Hz, 1H; *a*), 7.35 (d, *J* = 8.5 Hz, 2H; *h*), 7.33 – 7.29 (m, 9H; *c,5,16*), 7.28 (d, *J* = 9.0 Hz, 2H; *15*), 7.24 – 7.14 (m, 4H; *j,14*), 7.12 – 7.06 (m, 6H; *17*), 6.66 (s, 2H; *6*), 6.56 (s, 1H; 10^{Trz}), 6.37 (d, *J* = 8.3 Hz, 2H; *8*), 4.22 – 4.13 (m, 2H; *9*), 4.09 – 3.99 (m, 4H; *d*), 3.79 – 3.69 (m, 4H; *g*), 1.73 – 1.57 (m, 4H, *e*), 1.47 (s, 6H; *7*) 1.39 (s, 36H; *1*), 1.36 (s, 27H; *18*), 0.86 – 0.76 (m, 4H, *f*). (Signals of proton *11–13* missing due to peaks overlap).

^{13}C NMR (126 MHz, $CDCl_3$) δ (ppm): 158.80, 152.91, 151.30, 150.33, 150.13, 149.48, 149.38, 148.53, 148.33, 144.46, 142.03, 137.24, 137.04, 136.15, 134.52, 133.37, 132.16, 132.08, 131.99, 131.76, 131.57, 130.88, 130.80, 130.06, 129.61, 128.97, 128.86, 127.05, 125.88, 125.65, 124.37, 123.64, 121.96, 119.99, 119.20, 117.75, 115.78, 113.37, 112.85, 105.95, 105.84, 68.87, 63.05, 53.57, 49.98, 44.65, 35.04, 34.54, 31.66, 31.59, 29.86, 25.82, 25.61, 22.85, 15.20, 14.28.

HRMS (ESI +ve) m/z: 2466.94248 ($[M+H]^+$, $C_{141}H_{148}O_3N_{15}BF_2I_2Zn$ requires 2466.93668)

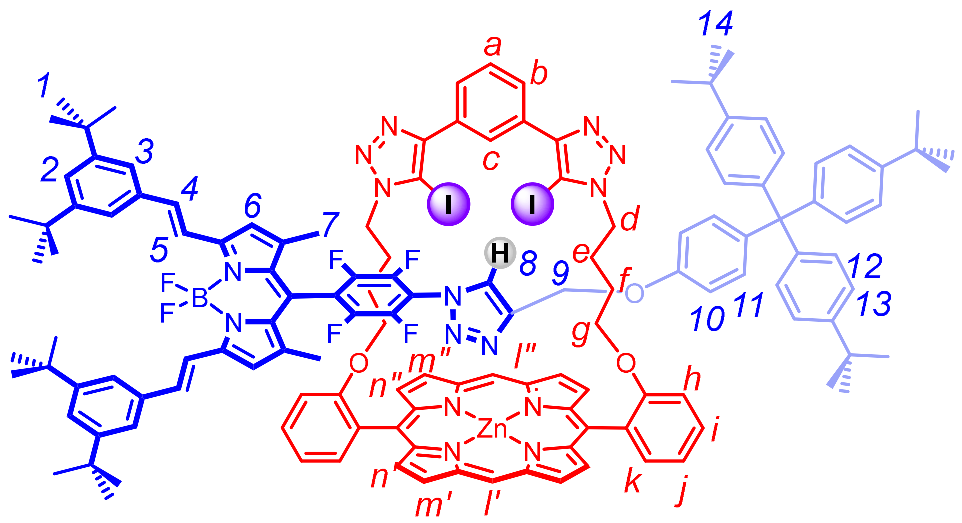

4.Zn

Following general procedure for CuAAC-AMT, strapped porphyrin **1a.Zn** (10 mg, 0.009 mmol, 1 equiv) was reacted with BODIPY-stoppered azide **11** (37 mg, 0.044 mmol, 5 equiv) and terphenyl-stoppered alkyne **8** (24 mg, 0.044 mmol, 5 equiv). The crude was purified by preparative TLC (0.5% EtOAc in CH_2Cl_2) to give the product [2]rotaxane **4.Zn** as blue powder (8 mg, 37%).

^{1}H NMR (400 MHz, CD_2Cl_2) δ (ppm): 9.98 (s, 1H; *l'*), 9.57 (s, 1H; *l''*), 9.21 (d, *J* = 4.5 Hz, 2H; *m'*), 9.03 (d, *J* = 4.5 Hz, 2H; *m''*), 8.93 (d, *J* = 4.4 Hz, 2H; *n'*), 8.91 (d, *J* = 4.4 Hz, 2H; *n''*), 7.92 (d, *J* = 6.5 Hz, 2H; *b*), 7.80 (t, *J* = 7.5 Hz, 2H; *i*), 7.75 (d, *J* = 16.3 Hz, 2H; *4*), 7.51 – 7.46 (m, 7H; *2,3,a*), 7.45 – 7.34 (m, 12H; *5,12,h,k*), 7.25 (d, *J* = 8.6 Hz, 6H; *13*), 7.21 (d, *J* = 7.8 Hz, 2H; *j*), 7.13 (t, *J* = 1.8 Hz, 1H; *c*), 6.76 (s, 2H; *6*), 6.70 (d, *J* = 8.8 Hz, 2H; *11*), 6.15 (s, 1H; *8*), 4.61 (d, *J* = 8.7 Hz, 2H; *10*), 4.25 – 4.03 (m, 4H; *d*), 3.96 – 3.73 (m, 4H; *g*), 1.63 (s, 6H; *7*), 1.39 (s, 36H; *1*), 1.38 (s, 27H; *14*), 0.56 (s, 2H; *9*).

^{13}C NMR (126 MHz, $CDCl_3$) δ (ppm): 158.74, 154.38, 151.42, 150.29, 150.08, 149.52, 149.19, 149.08, 148.38, 148.17, 144.46, 140.07, 139.73, 138.91, 137.31, 135.89, 132.61, 132.36, 131.99, 131.71, 131.47, 131.18, 130.78, 130.68, 129.43, 128.65, 127.33, 126.54, 124.40, 124.17, 123.12, 122.19, 119.92, 118.82, 118.77, 118.55, 115.54, 113.14, 112.93, 105.63, 105.58, 68.72, 63.15, 50.28, 35.04, 34.55, 31.67, 31.56, 29.85, 25.96, 25.74, 25.65, 14.01, 1.17.

^{19}F NMR (376 MHz, CD_2Cl_2) δ (ppm): -137.70 – -137.87 (m), -138.35 (q, *J* = 32.6 Hz), -145.01 – -145.18 (m).

HRMS (ESI +ve) m/z: 2510.8677 ($[M+H]^+$ $C_{139}H_{140}BF_6I_2N_{15}O_3Zn$ requires 2510.8670).

S2 Spectral Characterisation of Novel Compounds

Spectral Characterisation of XB Strapped Porphyrins and Precursors

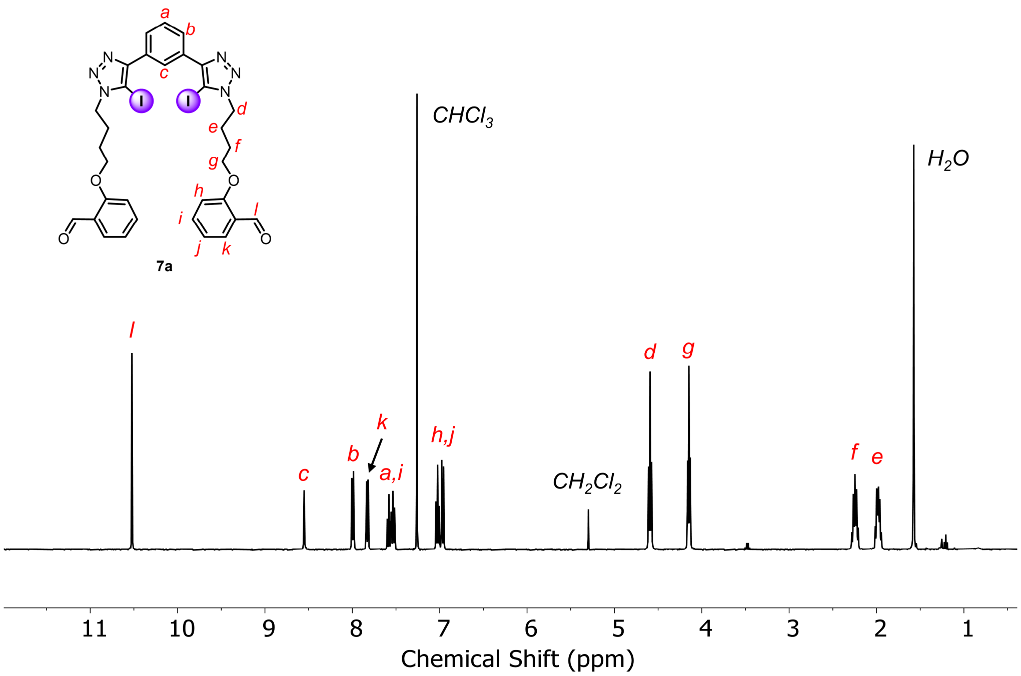

Figure S2-1. ^{1}H NMR spectrum of **7a** (400 MHz, 298 K, $CDCl_3$).

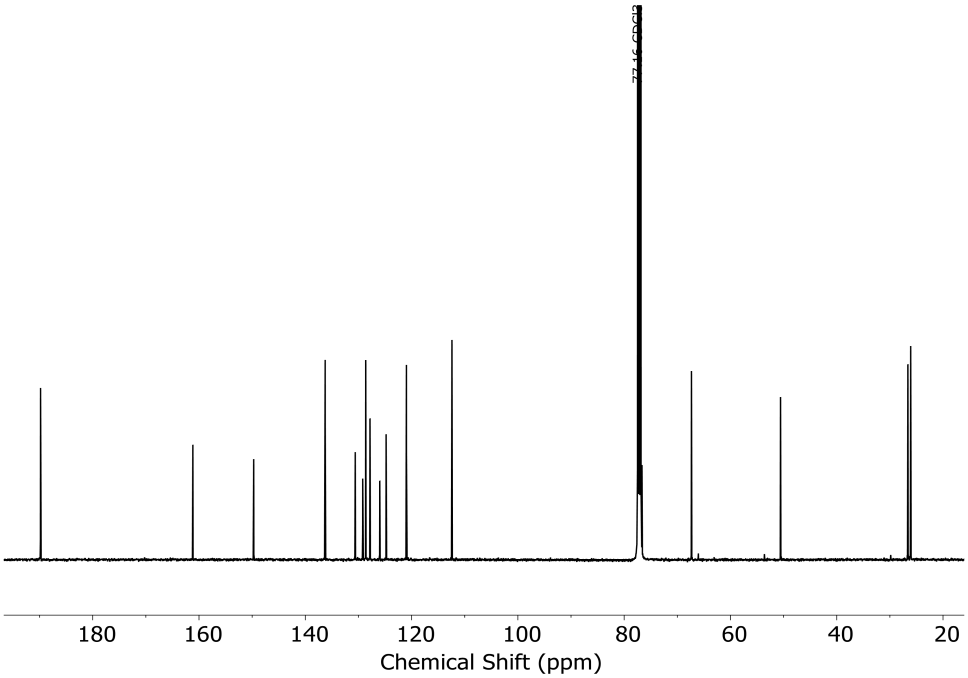

Figure S2-2. ^{13}C NMR spectrum of **7a** (126 MHz, 298 K, $CDCl_3$).

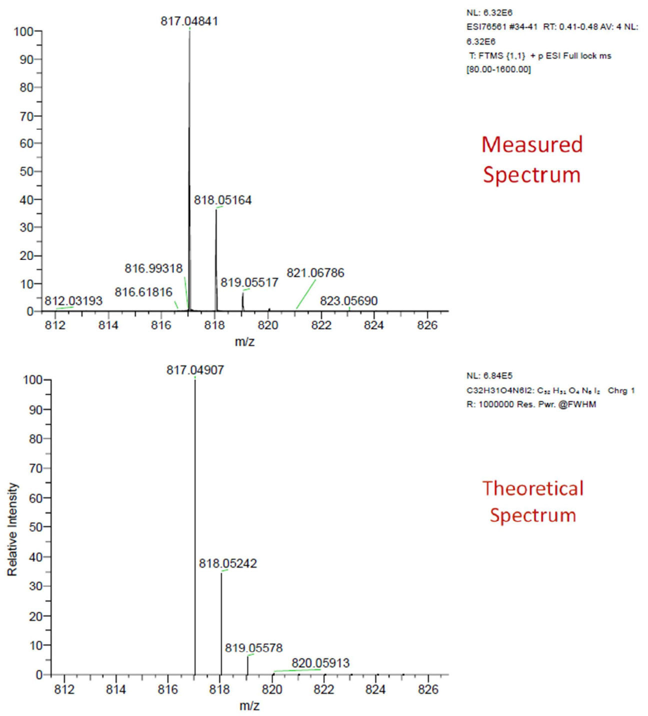

Figure S2-3. Measured (top) and theoretical (bottom) high resolution ESI mass spectrum of **7a**.

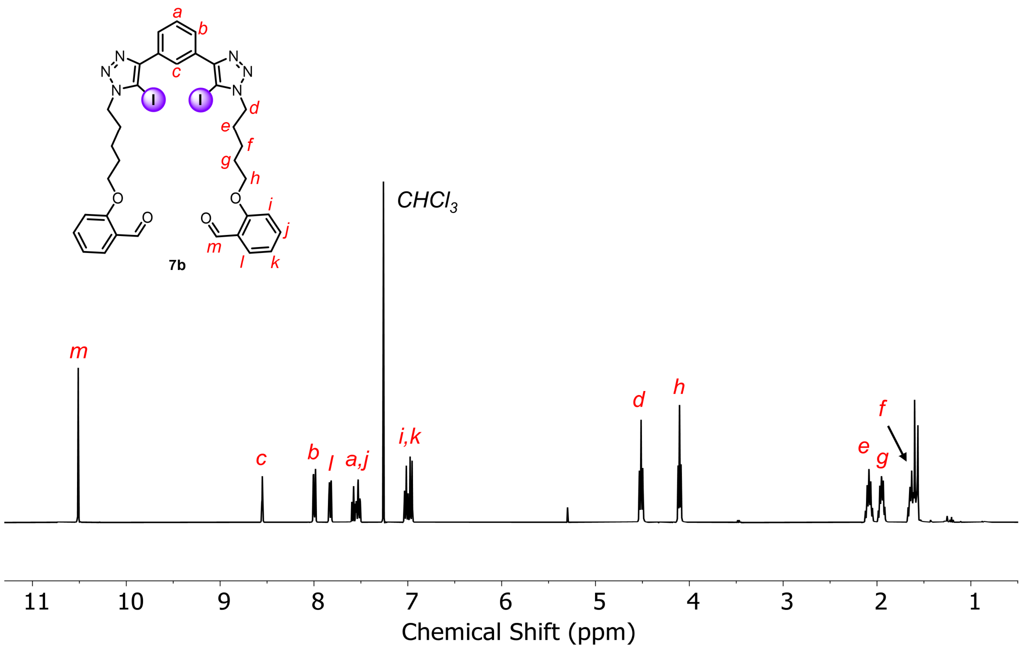

Figure S2-4. ^{1}H NMR spectrum of **7b** (400 MHz, 298 K, $CDCl_3$).

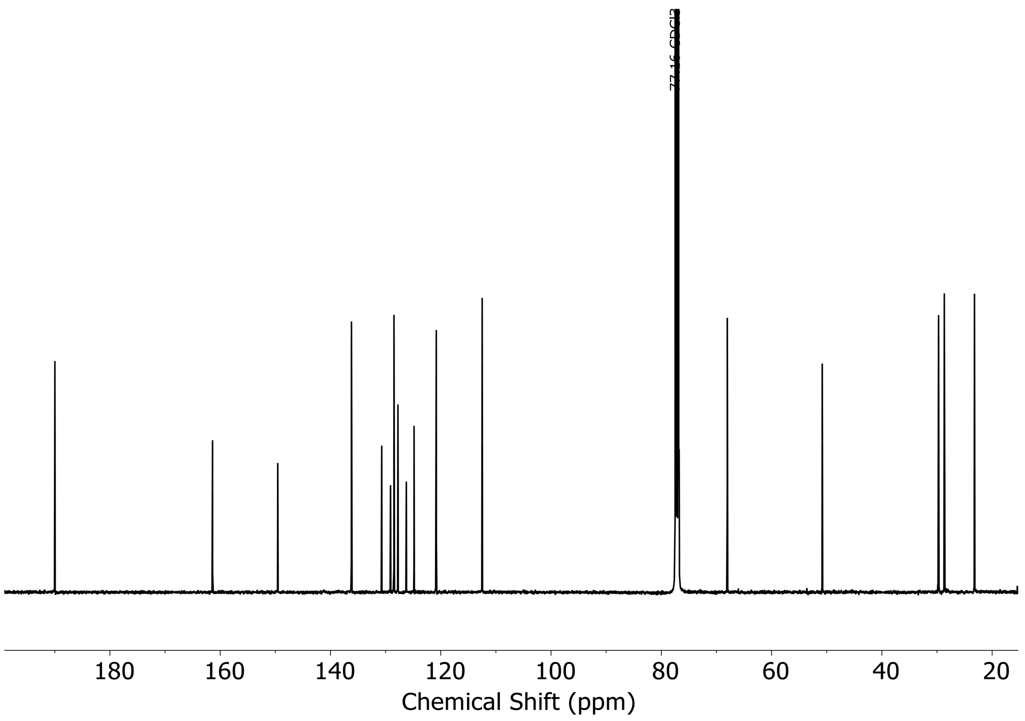

Figure S2-5. ^{13}C NMR spectrum of **7b** (126 MHz, 298 K, $CDCl_3$).

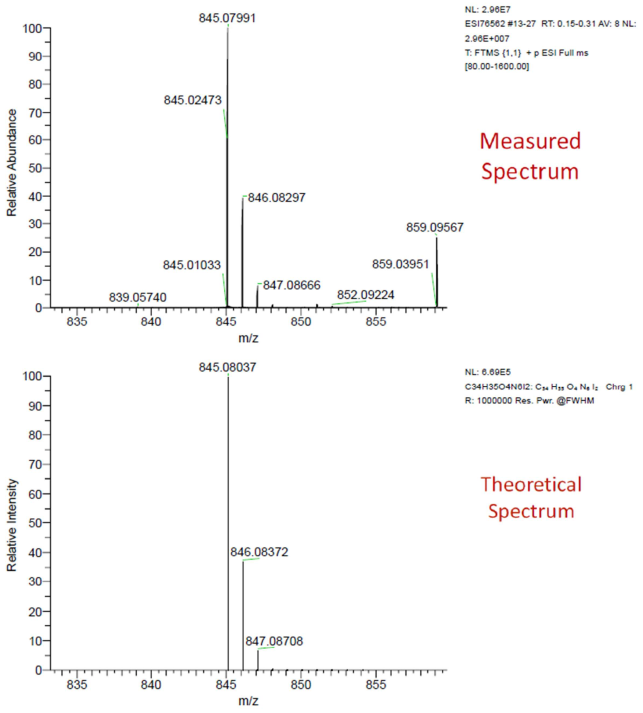

Figure S2-6. Measured (top) and theoretical (bottom) high resolution ESI mass spectrum of **7b**.

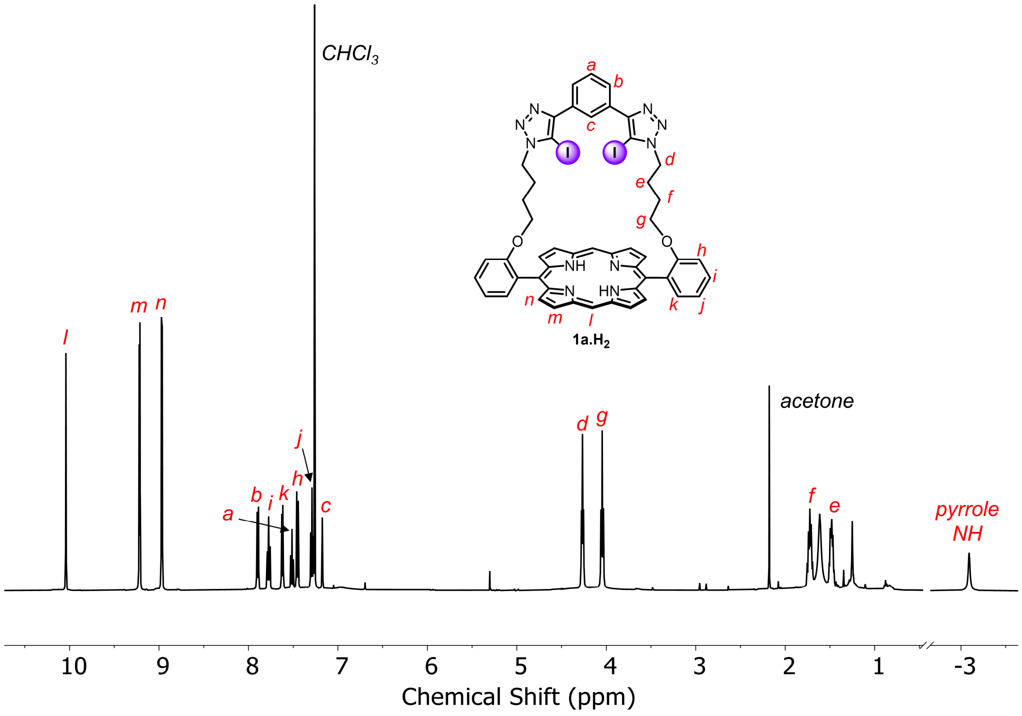

Figure S2-7. ^{1}H NMR spectrum of **1a.H$_2$** (500 MHz, 298 K, $CDCl_3$).

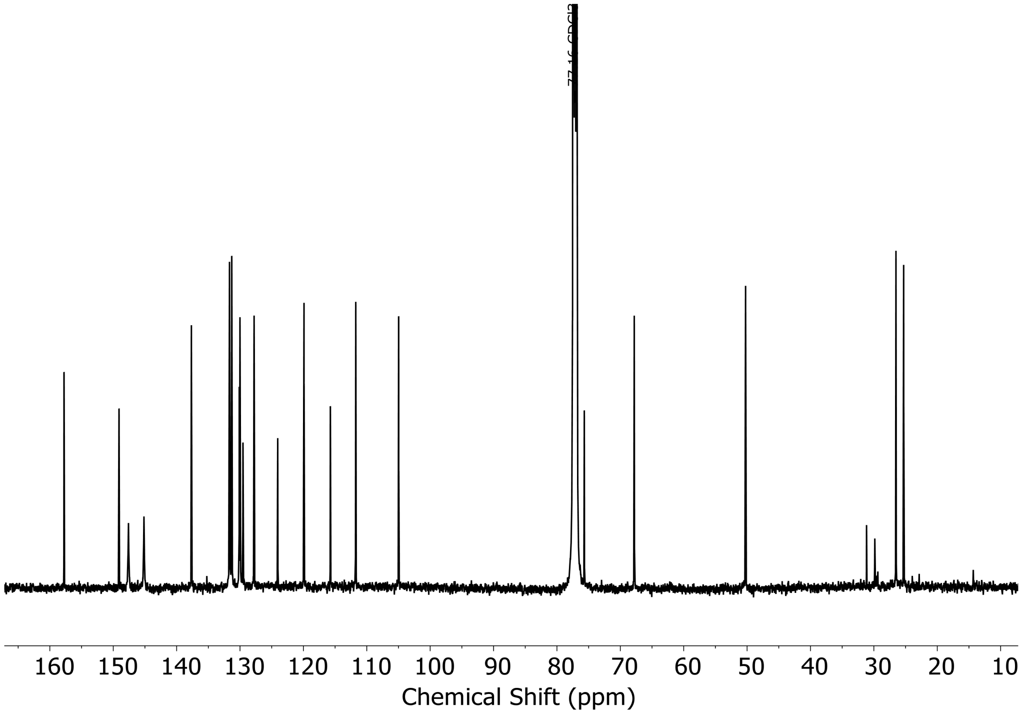

Figure S2-8. ^{13}C NMR spectrum of **1a.H$_2$** (126 MHz, 298 K, $CDCl_3$).

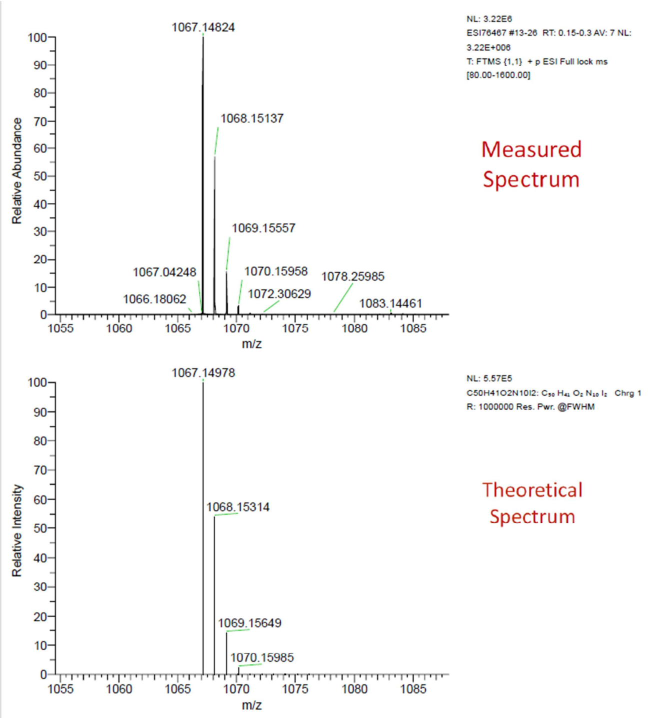

Figure S2-9. Measured (top) and theoretical (bottom) high resolution ESI mass spectrum of **1a.H₂**.

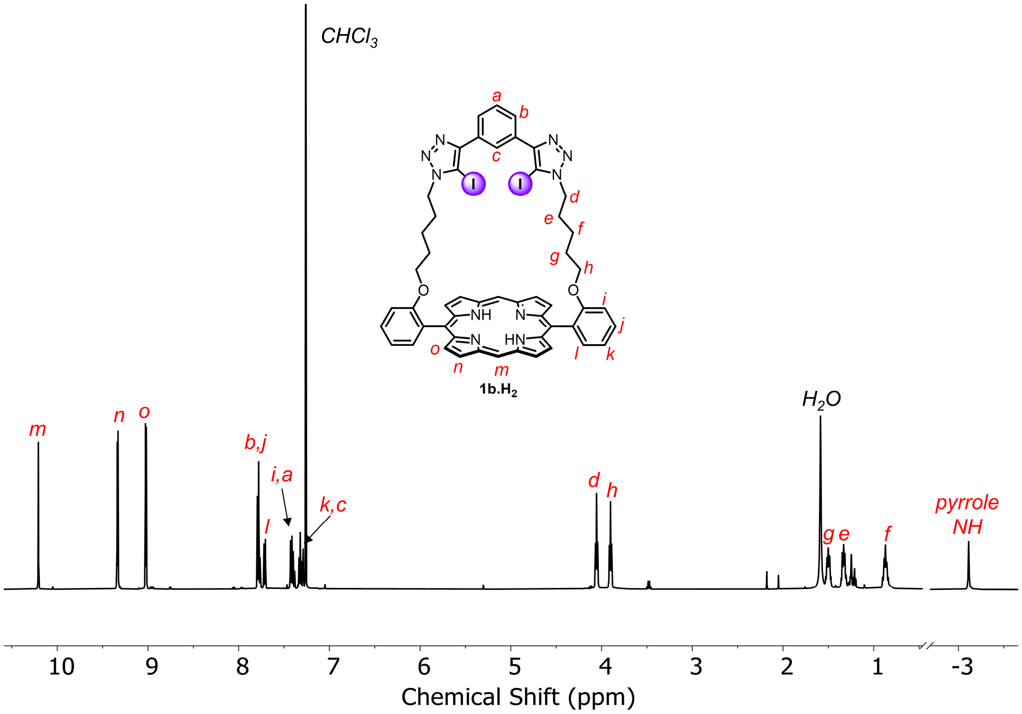

Figure S2-10. ^{1}H NMR spectrum of **1b.H$_2$** (500 MHz, 298 K, $CDCl_3$).

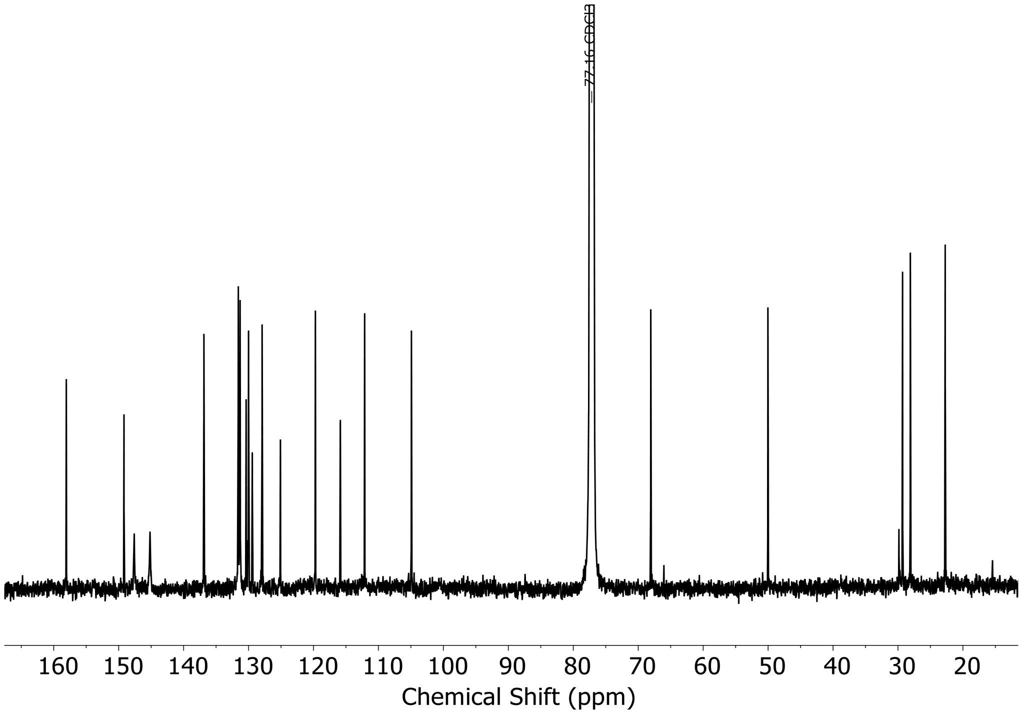

Figure S2-11. ^{13}C NMR spectrum of **1b.H$_2$** (126 MHz, 298 K, $CDCl_3$).

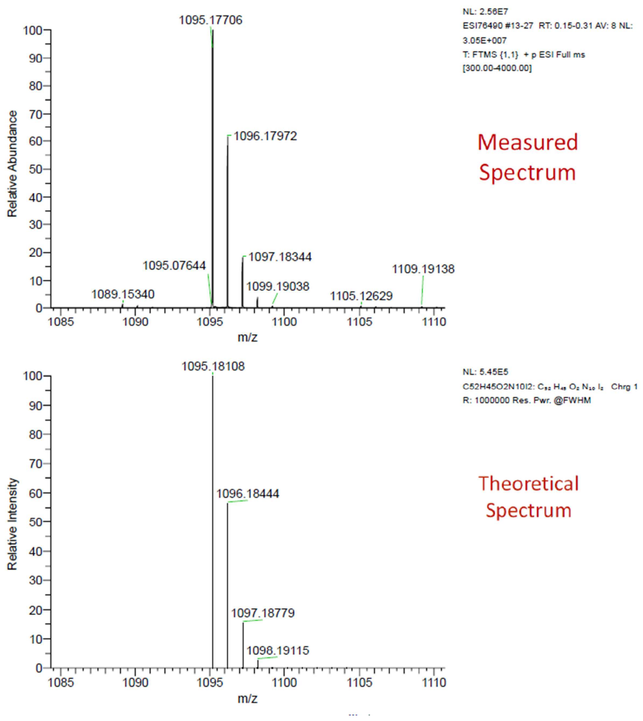

Figure S2-12. Measured (top) and theoretical (bottom) high resolution ESI mass spectrum of **1b.H$_2$**.

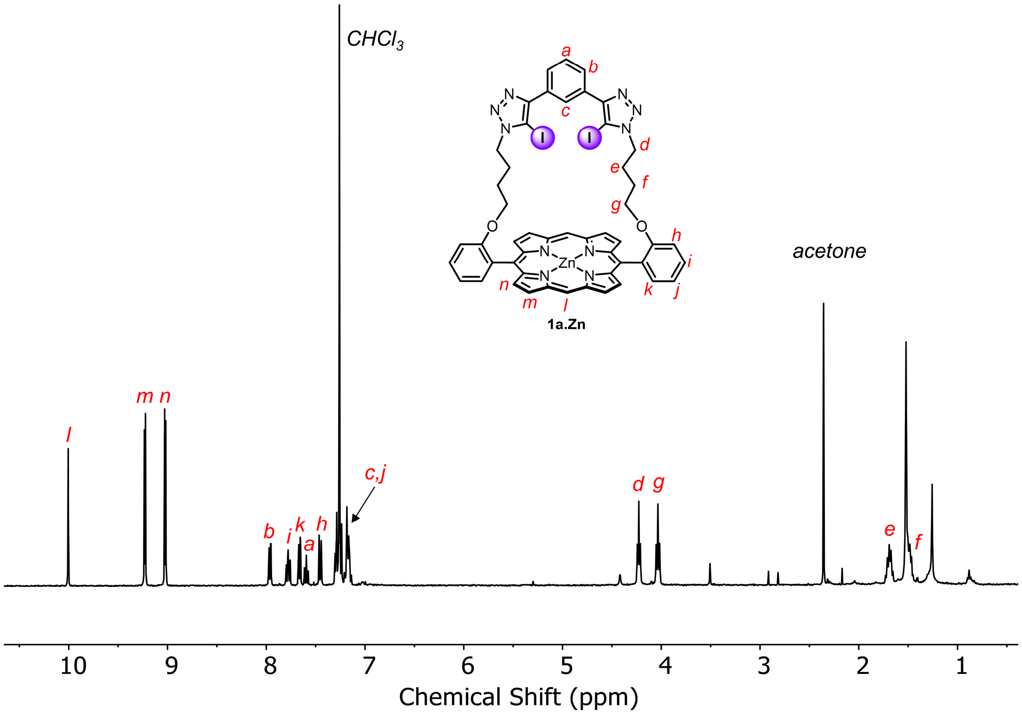

Figure S2-13. ^{1}H NMR spectrum of **1a.Zn** (500 MHz, 298 K, $CDCl_3$).

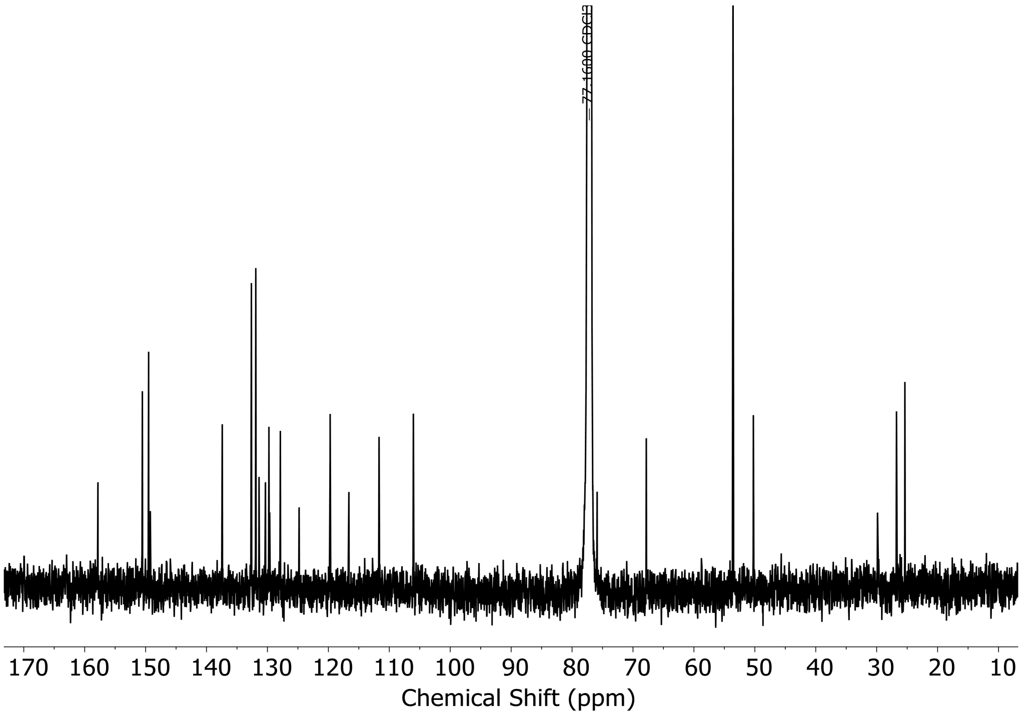

Figure S2-14. ^{13}C NMR spectrum of **1a.Zn** (126 MHz, 298 K, $CDCl_3$).

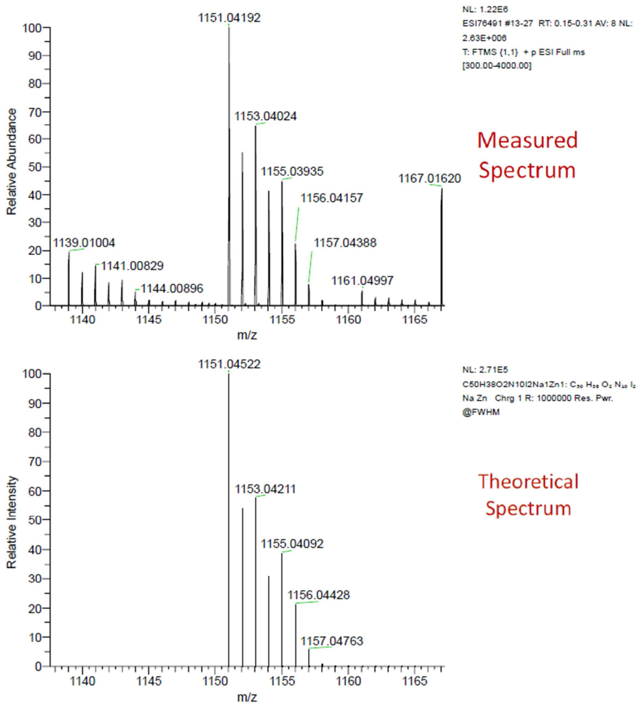

Figure S2-15. Measured (top) and theoretical (bottom) high resolution ESI mass spectrum of **1a.Zn**.

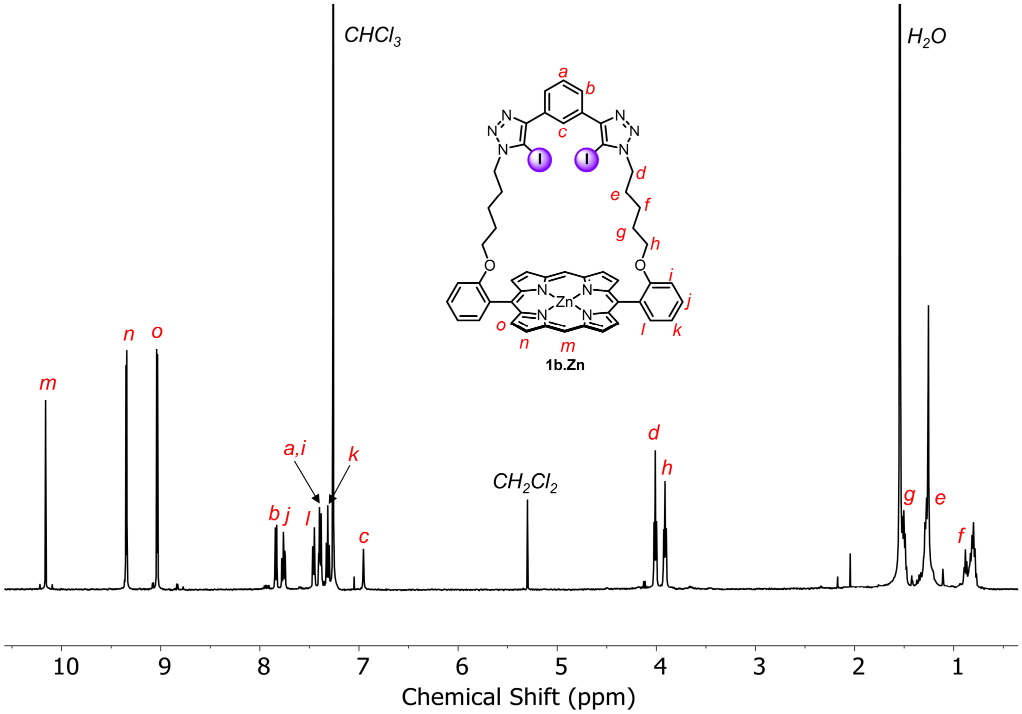

Figure S2-16. ^{1}H NMR spectrum of **1b.Zn** (500 MHz, 298 K, $CDCl_3$).

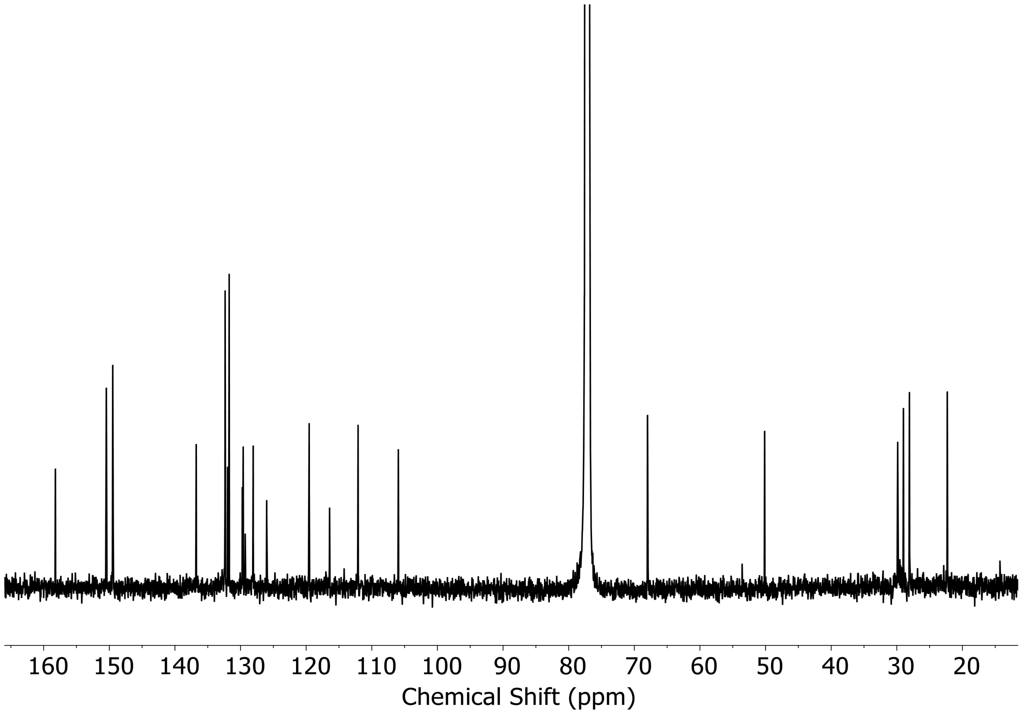

Figure S2-17. ^{13}C NMR spectrum of **1b.Zn** (126 MHz, 298 K, $CDCl_3$).

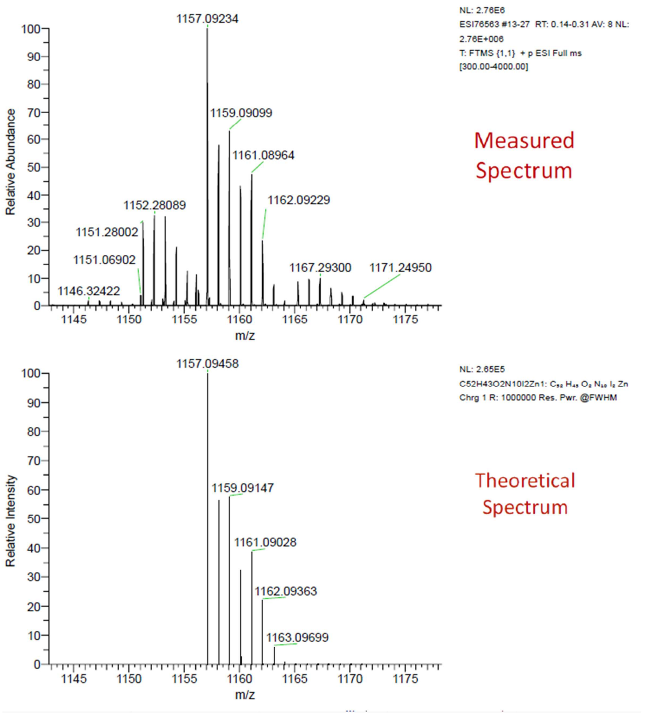

Figure S2-18. Measured (top) and theoretical (bottom) high resolution ESI mass spectrum of **1b.Zn**.

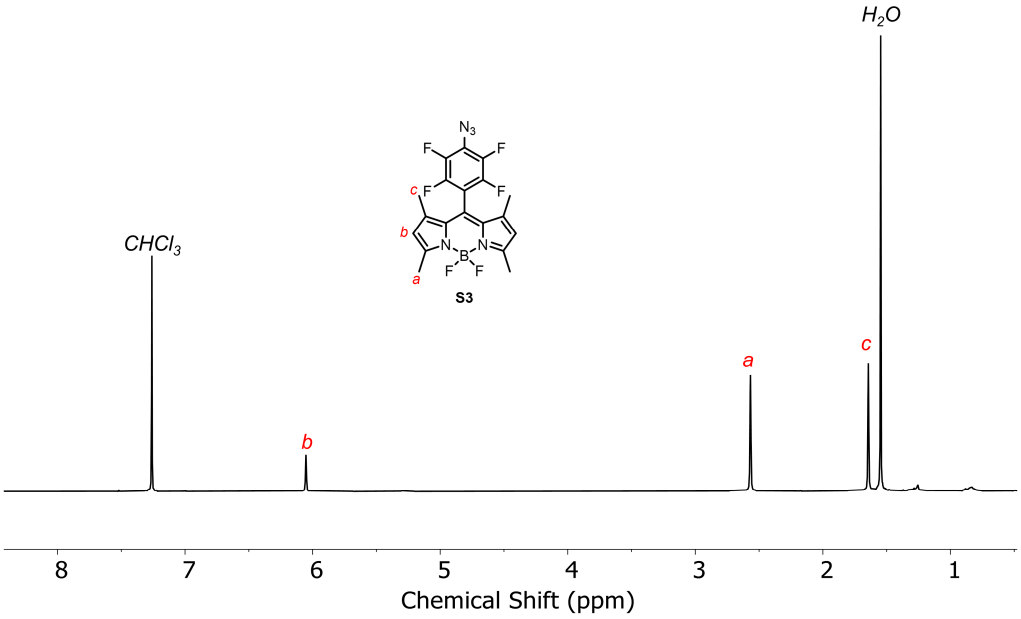

Figure S2-19. ^{1}H NMR spectrum of **S3** (400 MHz, 298 K, $CDCl_3$).

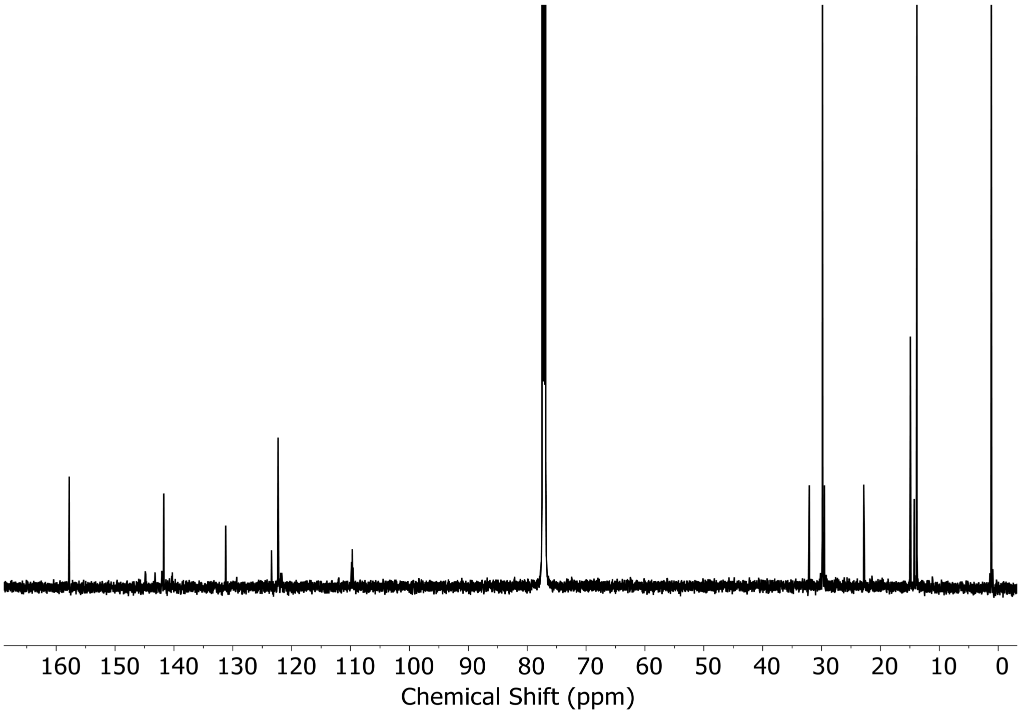

Figure S2-20. ^{13}C NMR spectrum of **S3** (151 MHz, 298 K, $CDCl_3$).

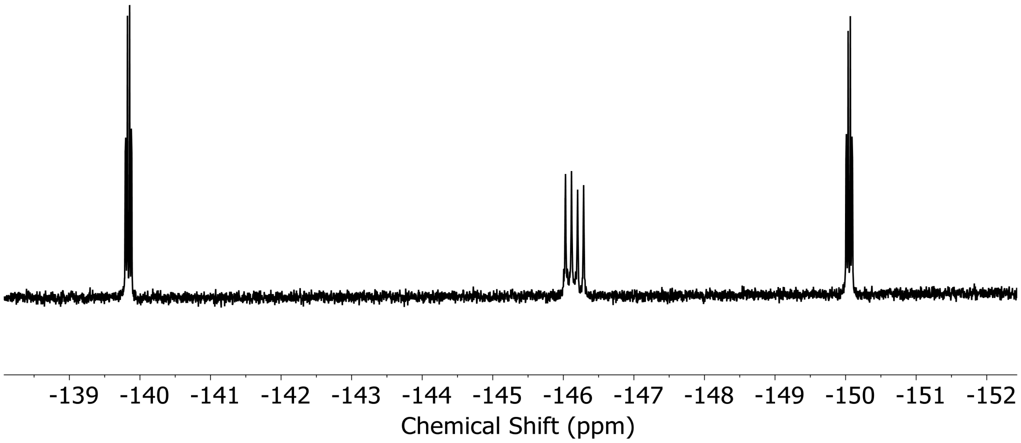

Figure S2-21. ^{19}F NMR spectrum of **S3** (376 MHz, 298 K, $CDCl_3$).

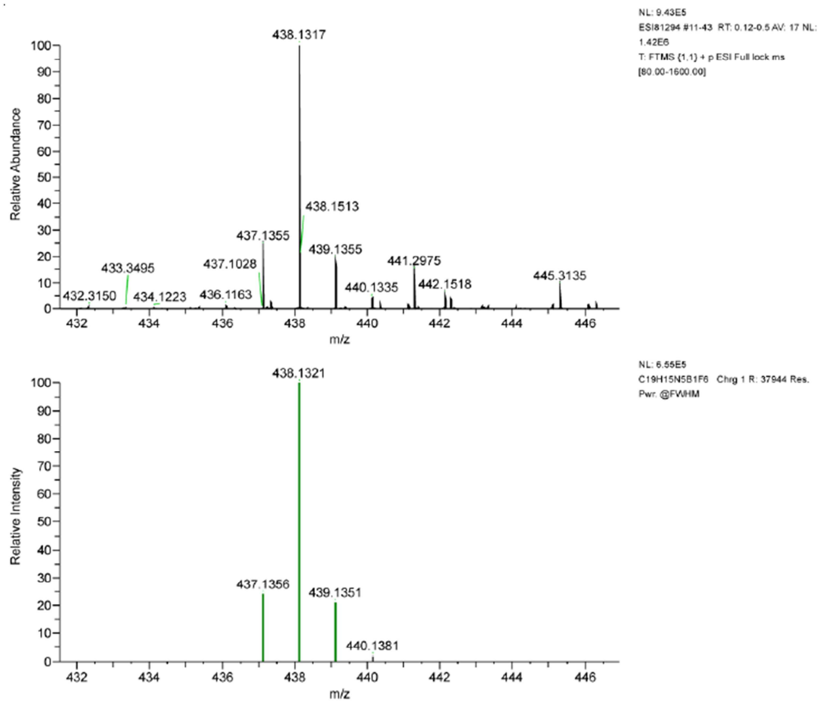

Figure S2-22. Measured (top) and theoretical (bottom) high resolution ESI mass spectrum of **S3**.

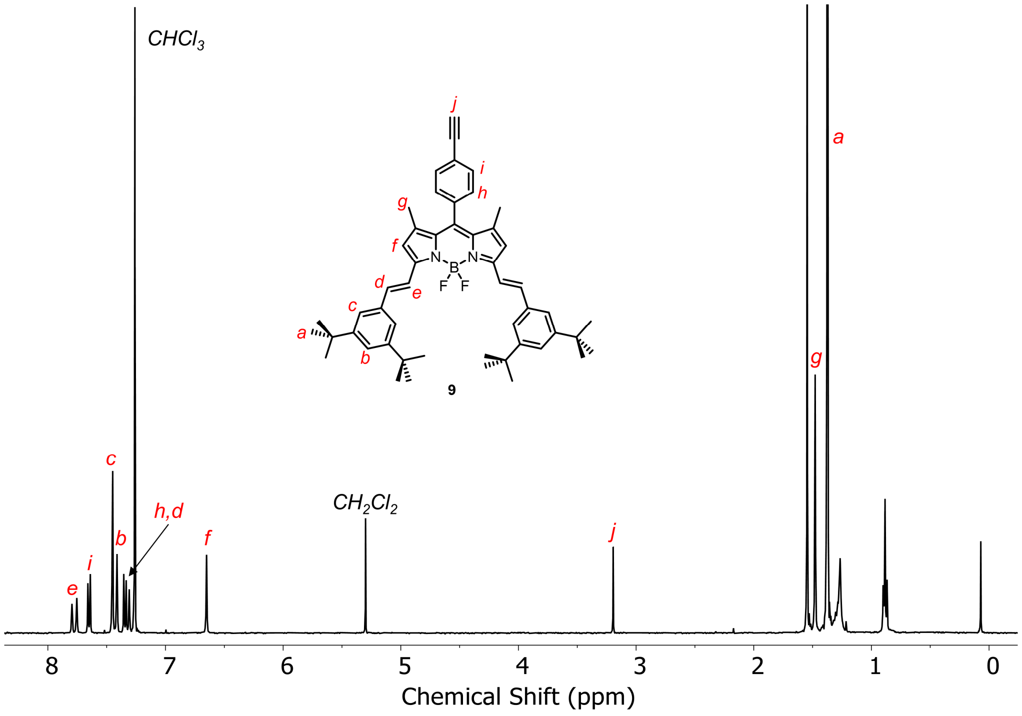

Figure S2-23. ^{1}H NMR spectrum of **9** (400 MHz, 298 K, $CDCl_3$).

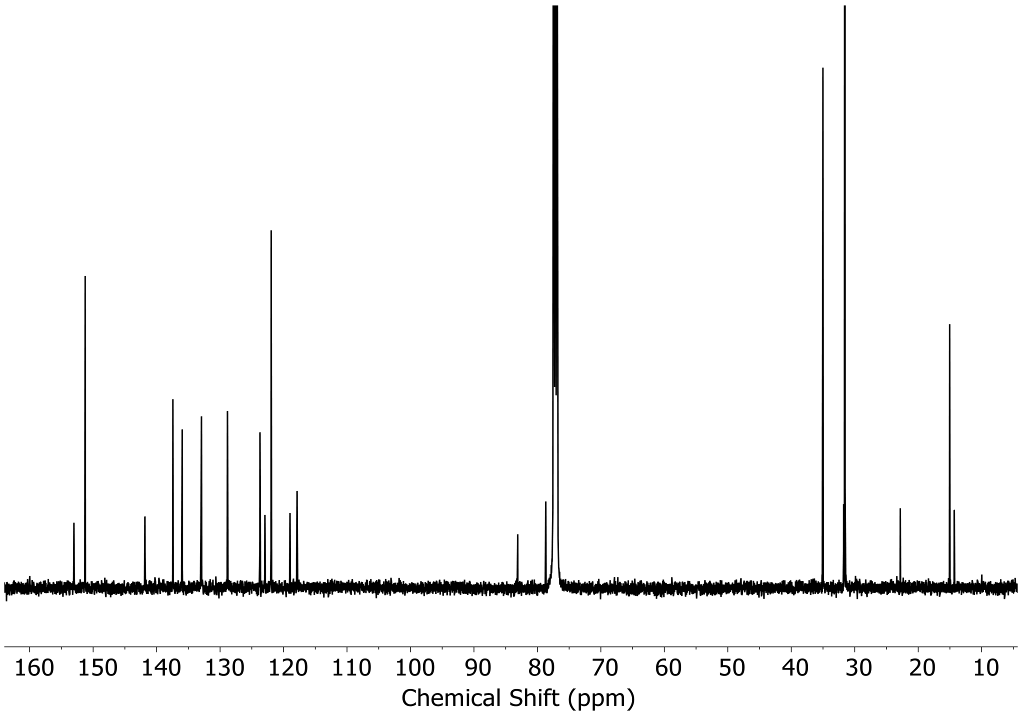

Figure S2-24. ^{13}C NMR spectrum of **9** (101 MHz, 298 K, $CDCl_3$).

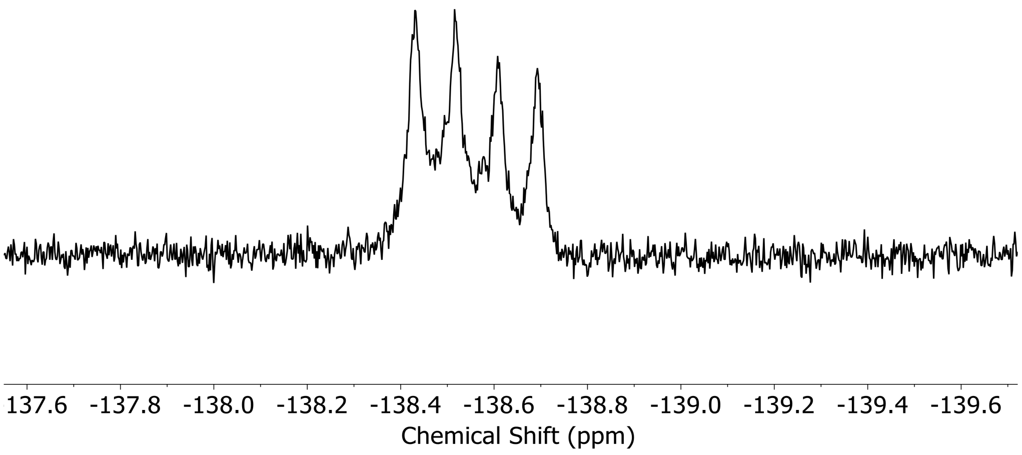

Figure S2-25. ^{19}F NMR spectrum of **9** (376 MHz, 298 K, $CDCl_3$).

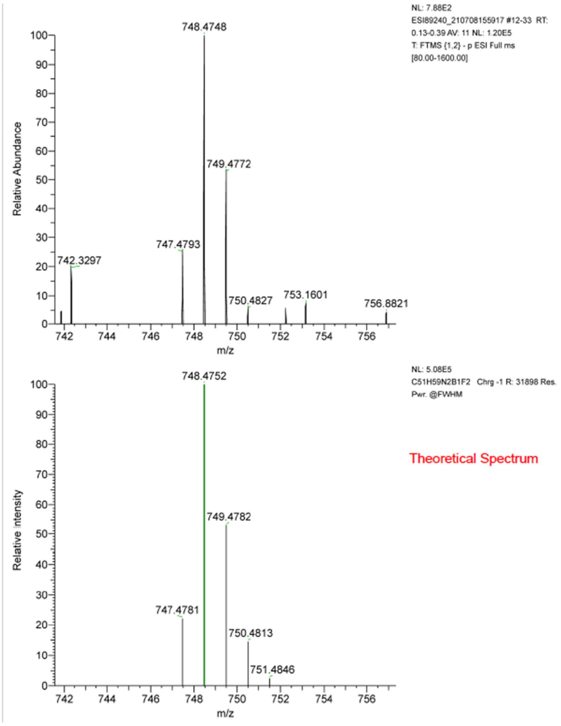

Figure S2-26. Measured (top) and theoretical (bottom) high resolution ESI mass spectrum of **9**.

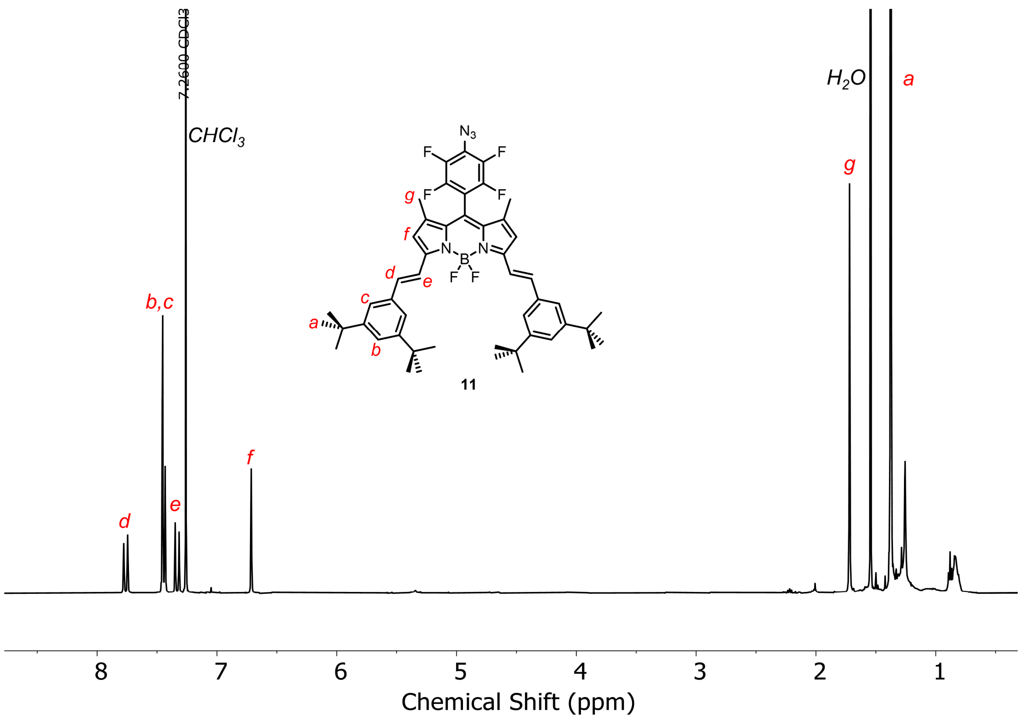

Figure S2-27. ^{1}H NMR spectrum of **11** (400 MHz, 298 K, $CDCl_3$).

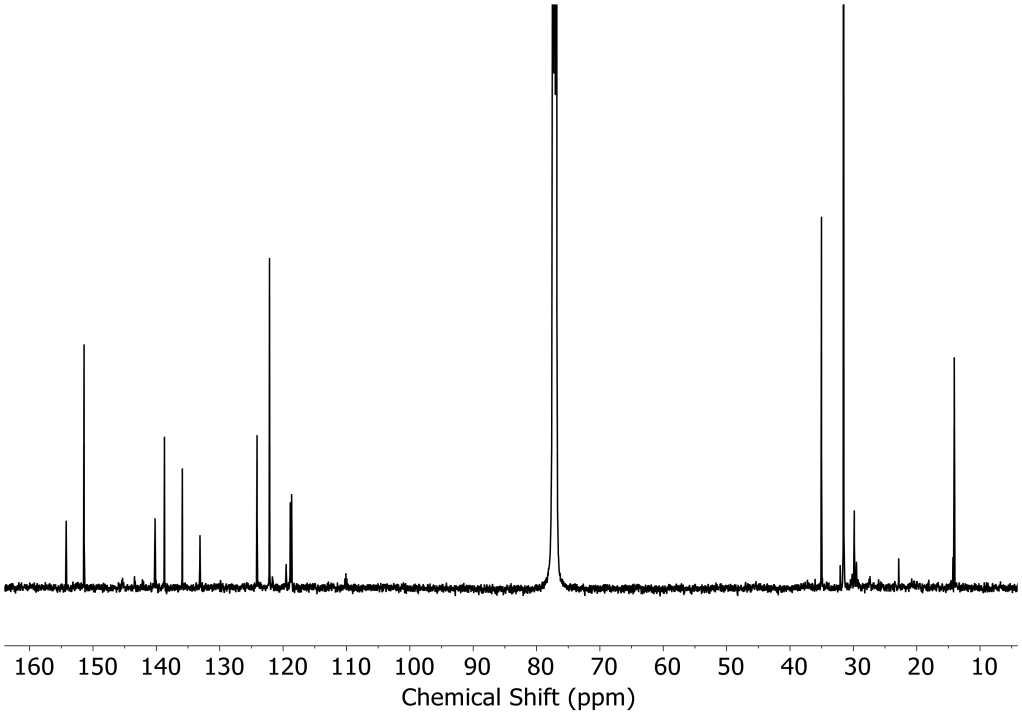

Figure S2-28. ^{13}C NMR spectrum of **11** (126 MHz, 298 K, $CDCl_3$).

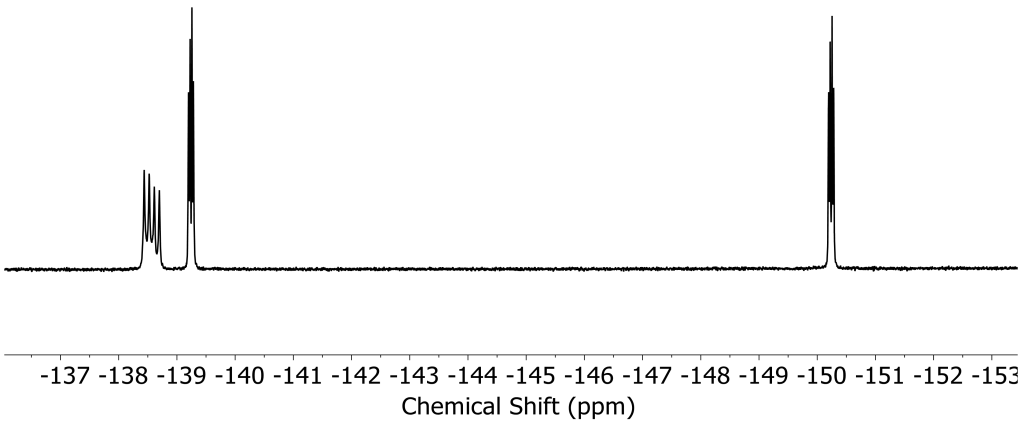

Figure S2-29. ^{19}F NMR spectrum of **11** (376 MHz, 298 K, $CDCl_3$).

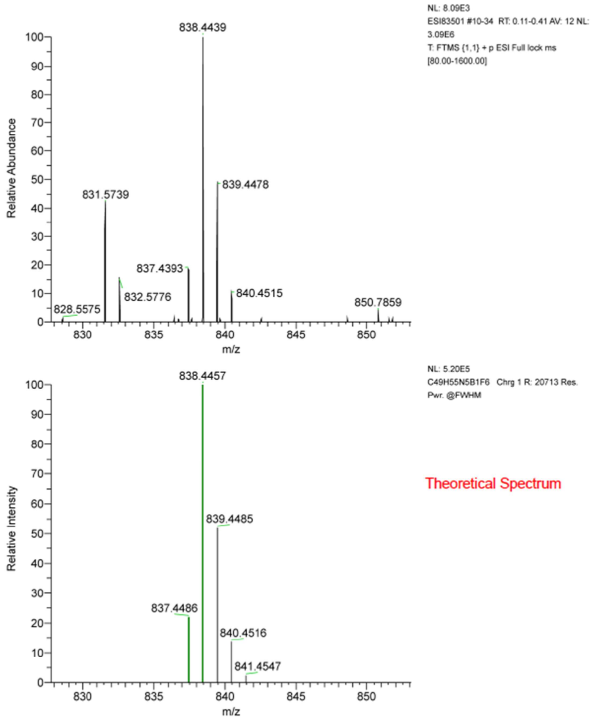

Figure S2-30. Measured (top) and theoretical (bottom) high resolution ESI mass spectrum of **11**.

Spectral Characterisation of XB [2]Rotaxanes

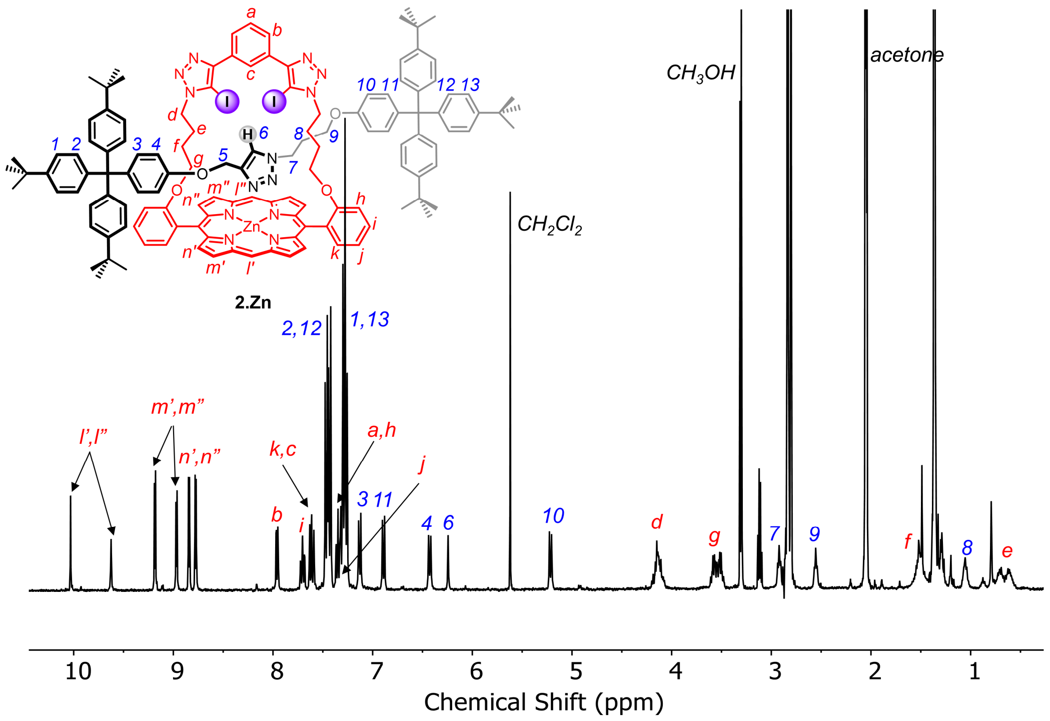

Figure S2-31. ^{1}H NMR spectrum of **2.Zn** (400 MHz, 298 K, acetone-d_6).

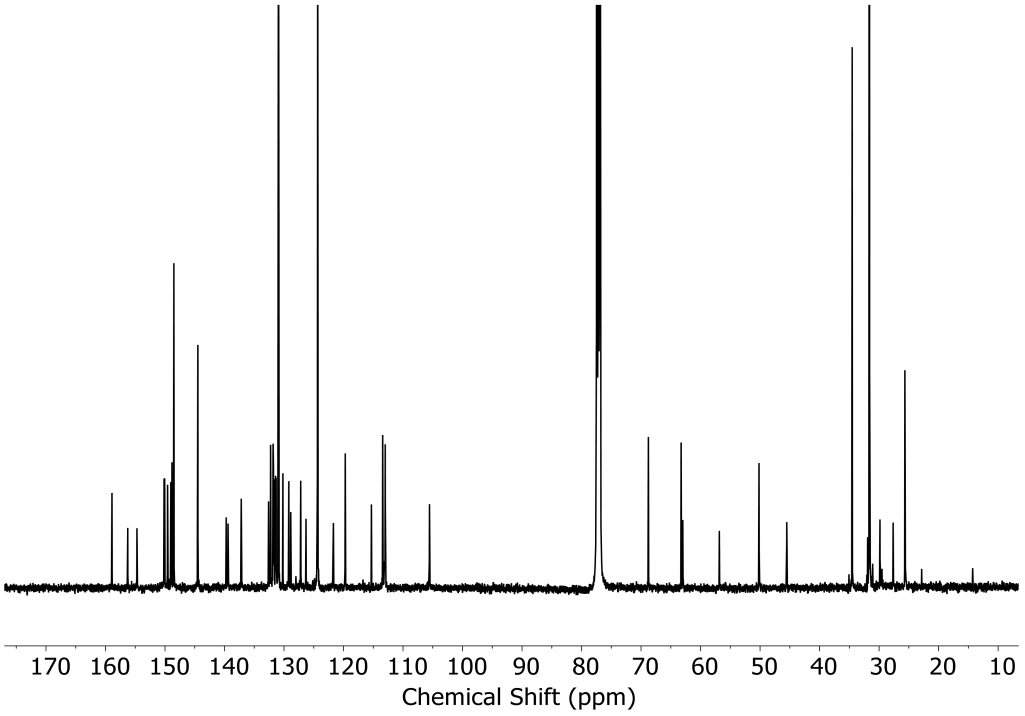

Figure S2-32. ^{13}C NMR spectrum of **2.Zn** (126 MHz, 298 K, $CDCl_3$).

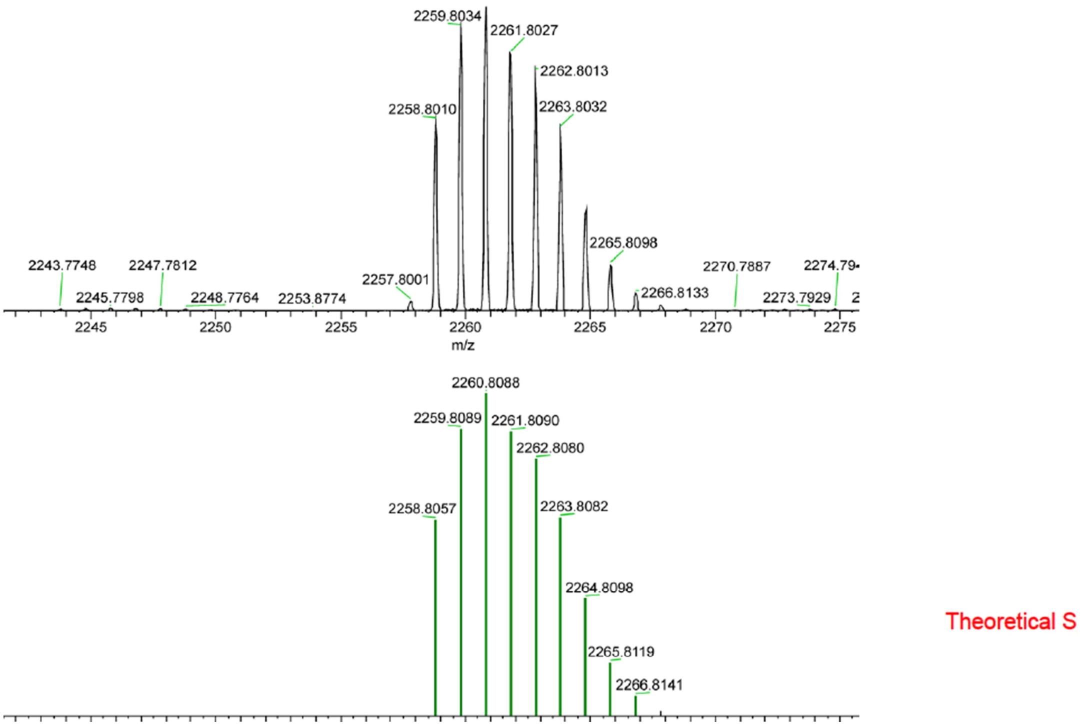

Figure S2-33. Measured (top) and theoretical (bottom) high resolution ESI mass spectrum of **2.Zn**.

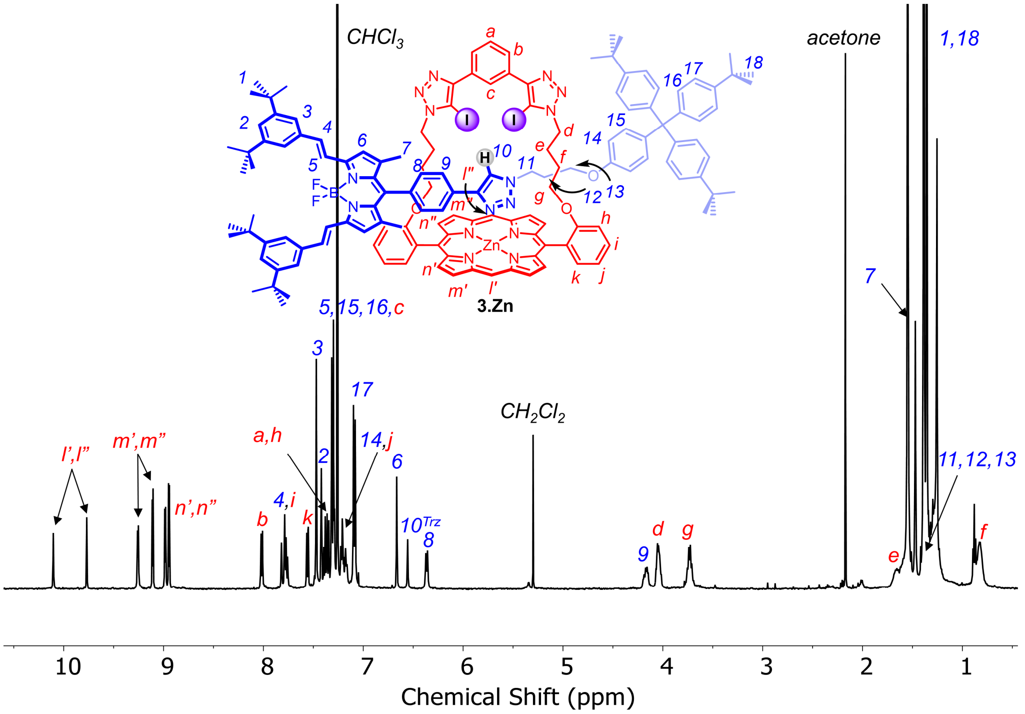

Figure S2-34. ^{1}H NMR spectrum of **3.Zn** (500 MHz, 298 K, $CDCl_3$).

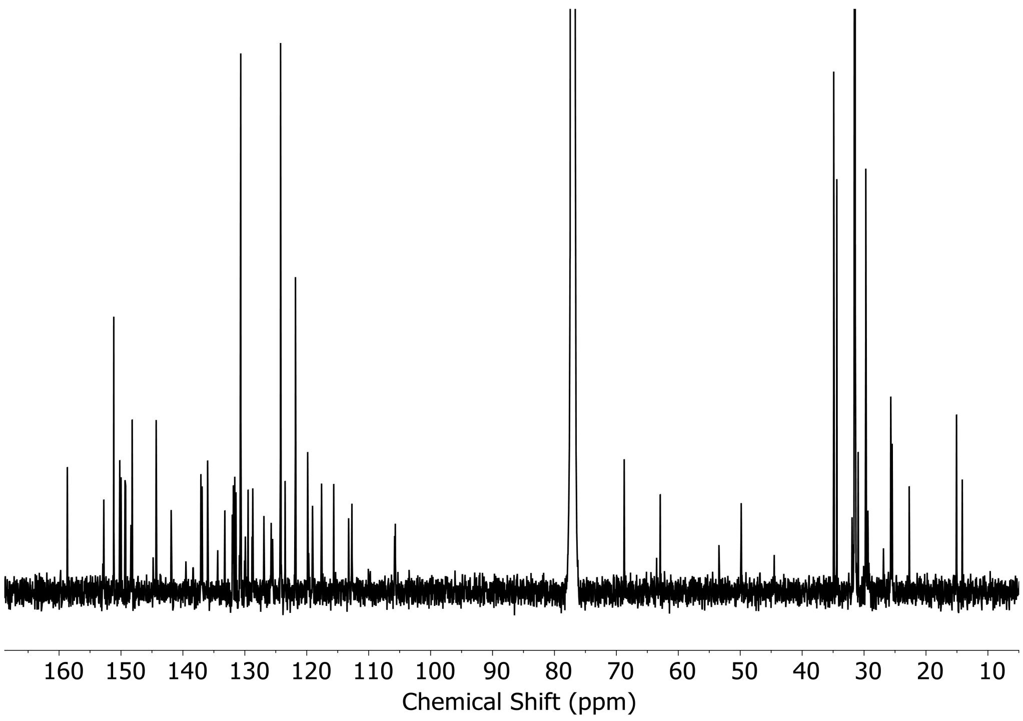

Figure S2-35. ^{13}C NMR spectrum of **3.Zn** (126 MHz, 298 K, $CDCl_3$).

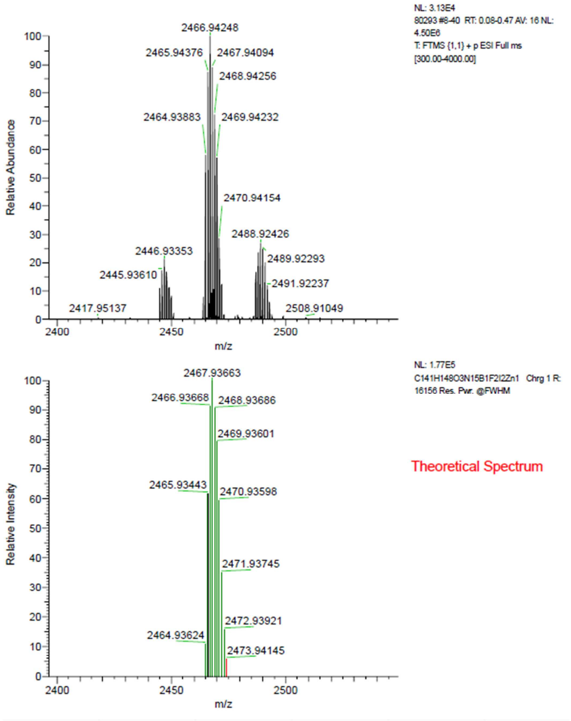

Figure S2-36. Measured (top) and theoretical (bottom) high resolution ESI mass spectrum of **3.Zn**.

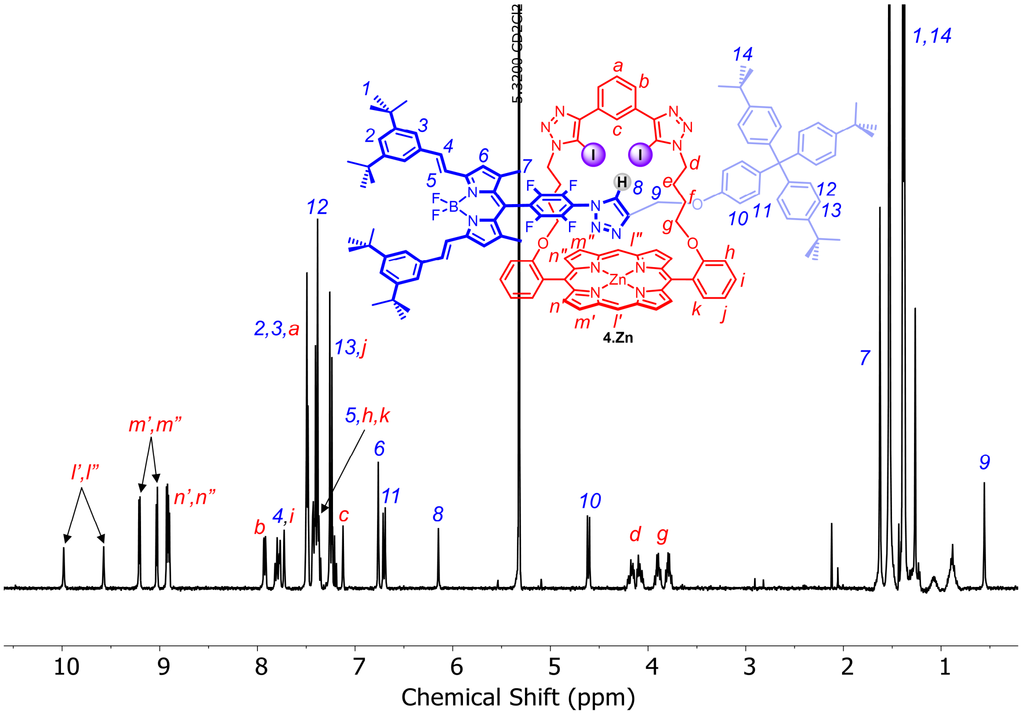

Figure S2-37. ^{1}H NMR spectrum of **4.Zn** (400 MHz, 298 K, CD_2Cl_2).

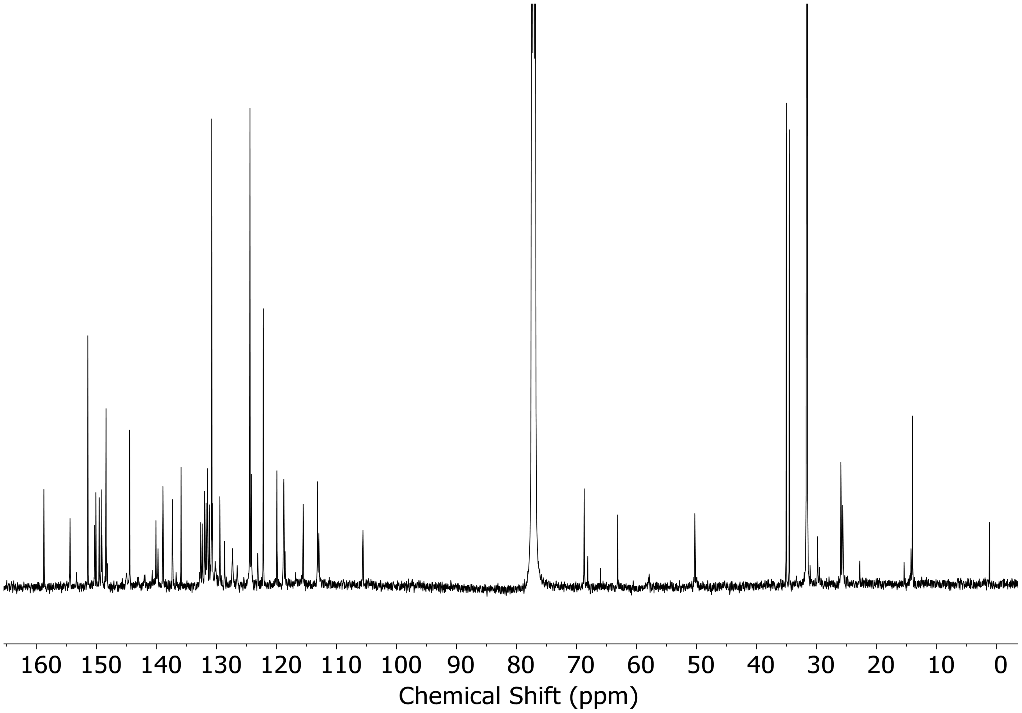

Figure S2-38. ^{13}C NMR spectrum of **4.Zn** (126 MHz, 298 K, $CDCl_3$).

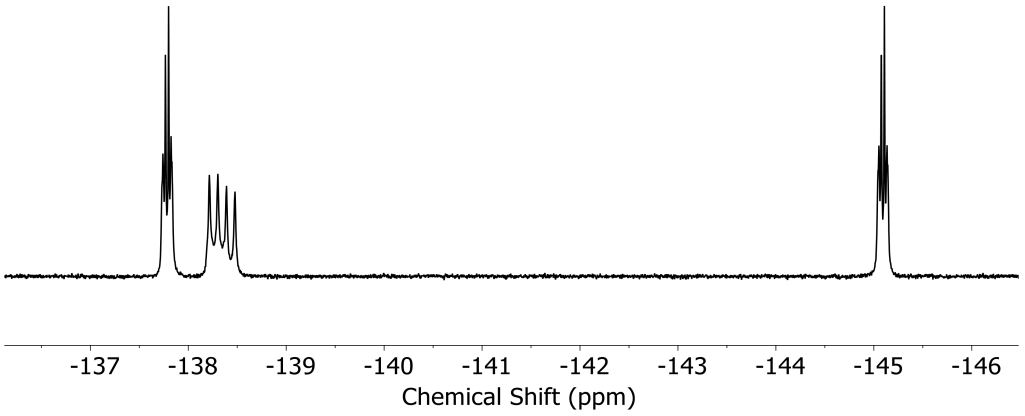

Figure S2-39. ^{19}F NMR spectrum of **4.Zn** (376 MHz, 298 K, CD_2Cl_2).

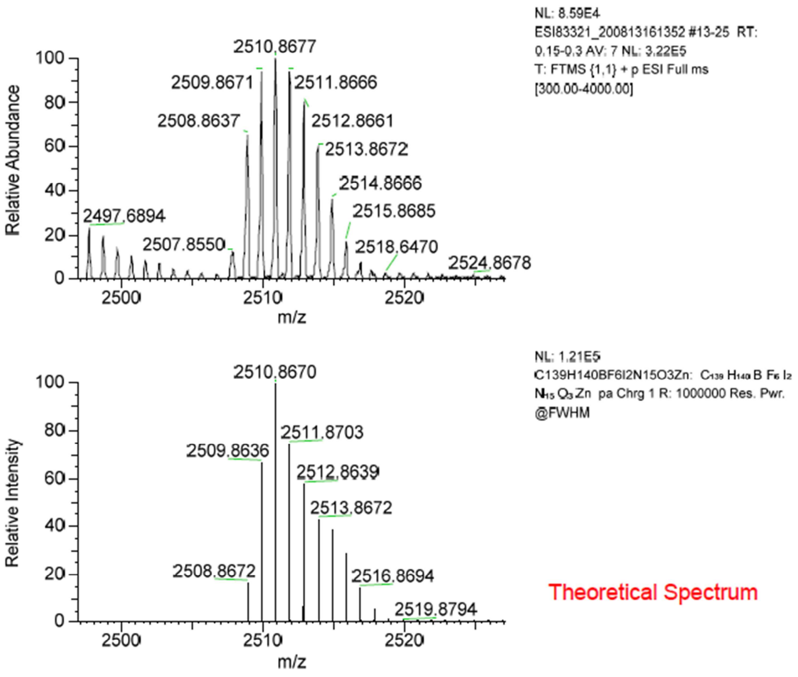

Figure S2-40. Measured (top) and theoretical (bottom) high resolution ESI mass spectrum of **4.Zn**.

S3 Single Crystal X-Ray Diffraction Studies

Single crystals of **1a.Zn** suitable for X-ray analysis were each coated with Paratone-N oil, suspended in a small fibre loop, and placed in a cold gaseous nitrogen stream on a Oxford Diffraction Supernova X-ray diffractometer performing ϕ- and ω-scans at 150(2) K. Diffraction intensities were measured using graphite monochromated Cu Kα radiation (λ = 1.54056 Å). Data collection, indexing, initial cell refinements, frame integration, final cell refinements and absorption corrections were accomplished using the program Crysalispro. Scattering factors and anomalous dispersion corrections were taken from the *International Tables for X-ray Crystallography*. All structures were solved by direct methods using SHELXS and refined against F^2 on all data by full-matrix least squares with SHELXL following established refinement strategies.[7]

All non-hydrogen atoms were refined anisotropically. All hydrogen atoms binding to carbon were included into the model at geometrically calculated positions and refined using a riding model. The isotropic displacement parameters of all hydrogen atoms were fixed to 1.2 times the U value of the atoms they are linked to (1.5 times for methyl groups). Details of the data quality and a summary of the residual values for the refinements are listed in Table S3-1. Crystal structure of **1a.Zn** is shown in Figure S3-1.

Deposition Number 2095235 (for **1a.Zn**) contains the supplementary crystallographic data for this paper. These data are provided free of charge by the joint Cambridge Crystallographic Data Centre and Fachinformationszentrum Karlsruhe.

Table S3-1. Crystal data and structure refinement for **1a.Zn**.

Identification code	**1a.Zn**	
Empirical formula	C52 H42 Cl4 I2 N10 O2 Zn	
Formula weight	1299.92	
Temperature	150(2) K	
Wavelength	1.54184 Å	
Crystal system	Monoclinic	
Space group	$P2_1/c$	
Unit cell dimensions	a = 23.4412(2) Å	α= 90°.
	b = 11.38600(10) Å	β= 93.3872(10)°.
	c = 18.6576(2) Å	γ = 90°.
Volume	4971.04(8) $Å^3$	
Z	4	
Density (calculated)	1.737 Mg/m^3	
Absorption coefficient	12.850 mm^{-1}	
F(000)	2576	
Crystal size	0.25 x 0.25 x 0.25 mm^3	
Theta range for data collection	3.778 to 76.424°.	
Index ranges	-29<=h<=28, -12<=k<=13, -23<=l<=22	
Reflections collected	56701	
Independent reflections	10299 [R(int) = 0.0716]	
Completeness to theta = 67.684°	100.0 %	
Absorption correction	Sphere	
Max. and min. transmission	0.08027 and 0.00973	
Refinement method	Full-matrix least-squares on F^2	
Data / restraints / parameters	10299 / 0 / 640	
Goodness-of-fit on F^2	1.066	
Final R indices [I>2sigma(I)]	R1 = 0.0699, wR2 = 0.1677	
R indices (all data)	R1 = 0.0715, wR2 = 0.1716	
Extinction coefficient	n/a	
Largest diff. peak and hole	3.033 and -0.870 e.$Å^{-3}$	

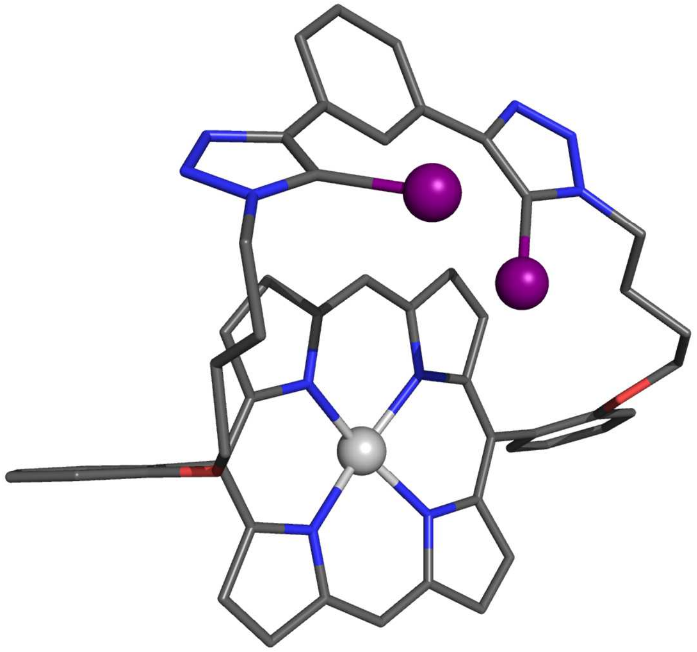

Figure S3-1. Crystal structure of **1a.Zn**. Hydrogen atoms are omitted for clarity. Gray = carbon, blue = nitrogen, red = oxygen, purple = iodine, silver = zinc.

S4 UV-Visible Absorption Studies

UV-Visible absorption Characterisation

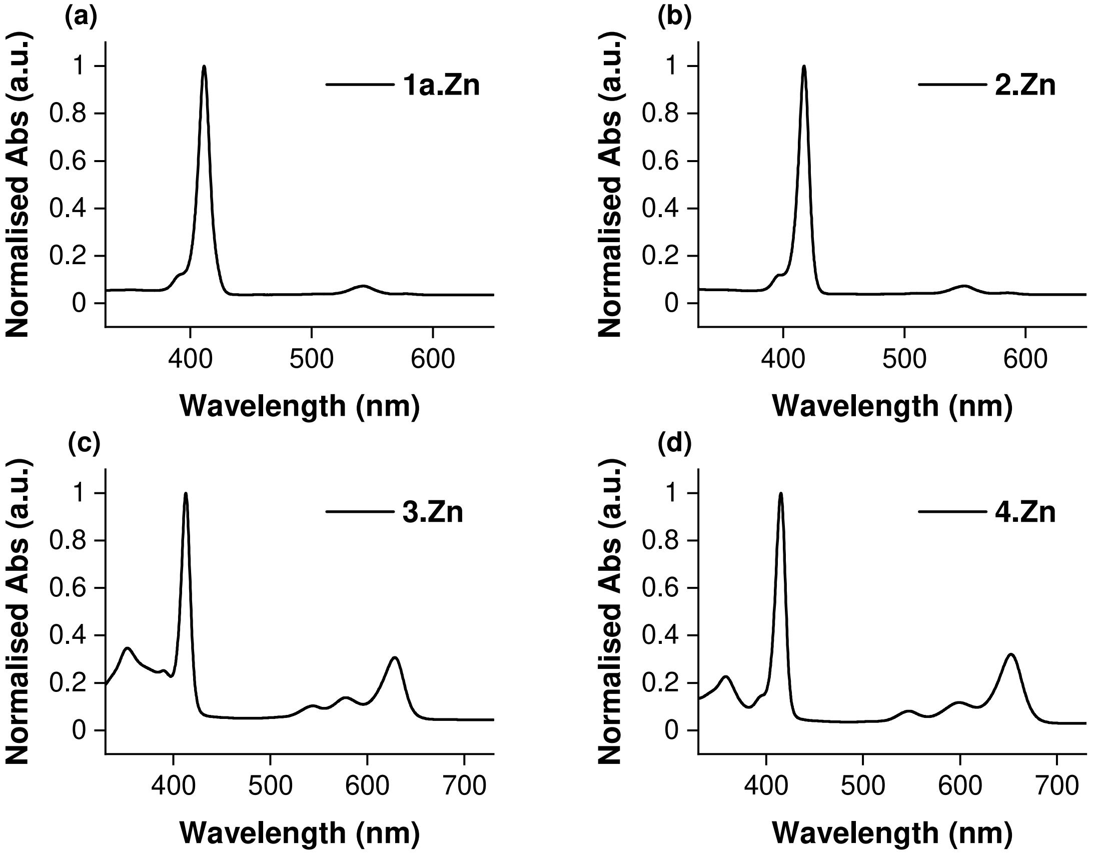

Figure S4-1. Normalised UV-visible absorption spectra of (a) **1a.Zn**, (b) **2.Zn**, (c) **3.Zn** and (d) **4.Zn** ([receptor] = 3 μM, 298 K, acetone).

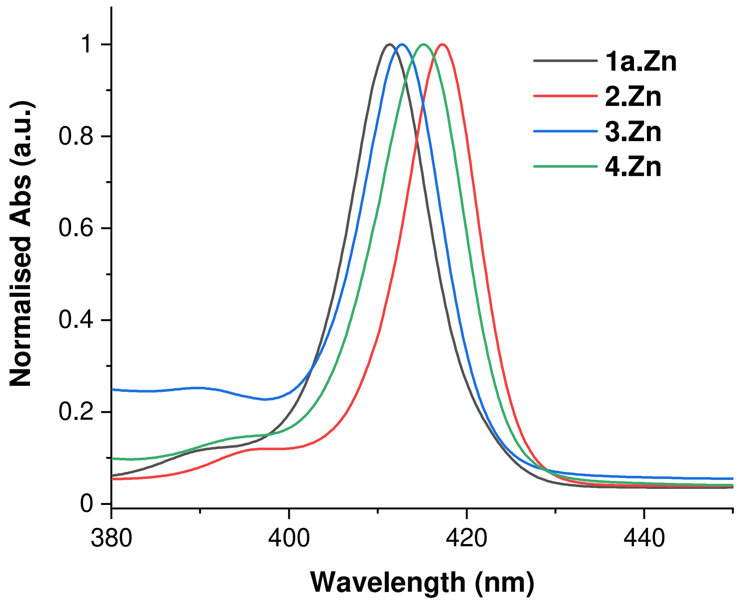

Figure S4-2. Comparative normalised UV-visible absorption spectra of (a) **1a.Zn**, (b) **2.Zn**, (c) **3.Zn** and (d) **4.Zn** ([receptor] = 3 μM, 298 K, acetone).

Effect of Pyridine Addition

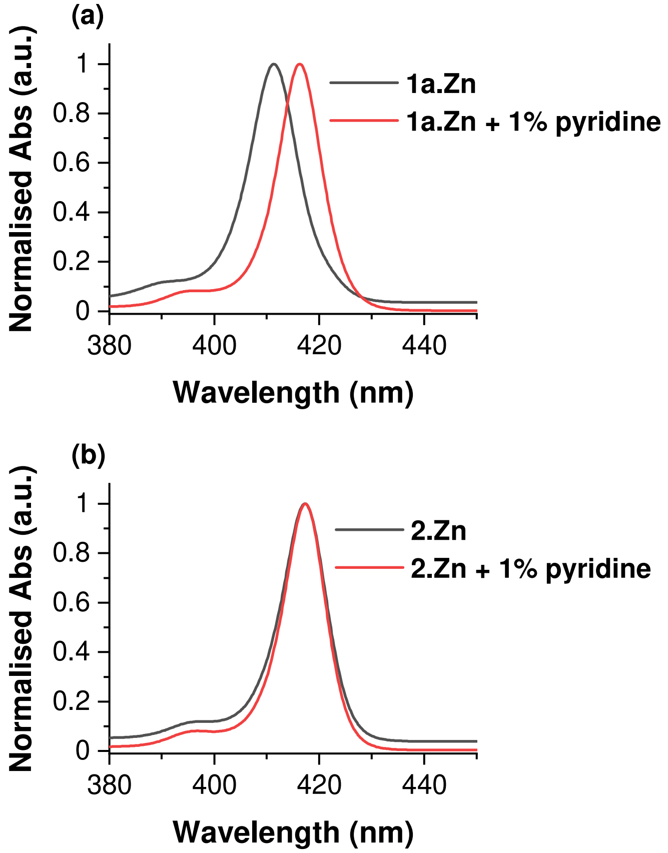

Figure S4-3. Effect of adding 1% pyridine to the normalised UV-visible absorption spectra of (a) **1a.Zn** and (b) **2.Zn** ([receptor] = 3 μM, 298 K, acetone).

S5 ^{1}H NMR Anion Binding Studies

General Procedures

^{1}H NMR titration experiments were performed on a Bruker AVIII 500 MHz spectrometer. In a typical experiment, a solution of the appropriate TBA salt was added to a solution of the receptor molecule at 298 K in acetone-d_6 or D_2O/acetone-d_6 (2% v/v). Both TBA salt and receptor were dissolved in the same solvent. TBA was chosen as the counter-cation due to its non-coordinating nature. A 0.05 M solution of the salt was added to 0.50 mL of a 1.0 mM solution of receptor, where 1.0 equivalent of salt added corresponds to 10.0 μL of the salt solution. 17 data points corresponding to 0.0, 0.2, 0.4, 0.6, 0.8, 1.0, 1.2, 1.4, 1.6, 1.8, 2.0, 2.5, 3.0, 4.0, 5.0, 7.0 and 10.0 equivalents of added guest anion were obtained. The binding of anions with all receptors were found to be fast on the NMR timescale.

Stability constants were obtained using BindFit,[8–10] using a host-guest 1:1 binding model. For the anion titrations, the recorded spectra during the titration are shown, followed by the binding isotherms obtained by monitoring the changes in the chemical shift of the internal benzene proton *c* and axle triazole proton *6* were monitored for titrations with **1a.Zn** and **2.Zn** respectively. Filled dots represent the experimental data, while the solid lines show the fitted data (calculated isotherm).

^{1}H NMR Titration Spectra

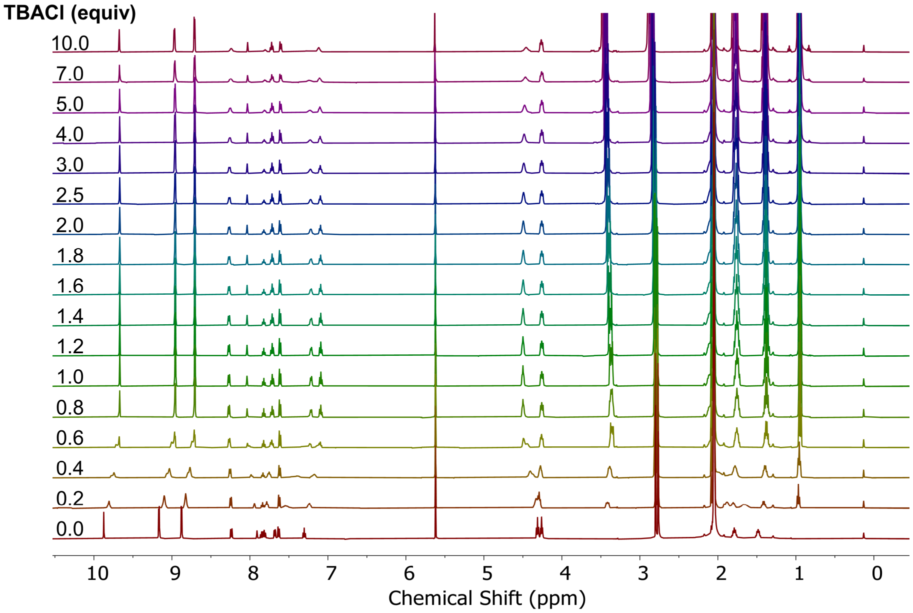

Figure S5-1. ^{1}H NMR titration spectra of **1a.Zn** upon addition of 10 equivalents of TBACl ([**1a.Zn**] = 1.0 mM, 500 MHz, 298 K, acetone-d_6).

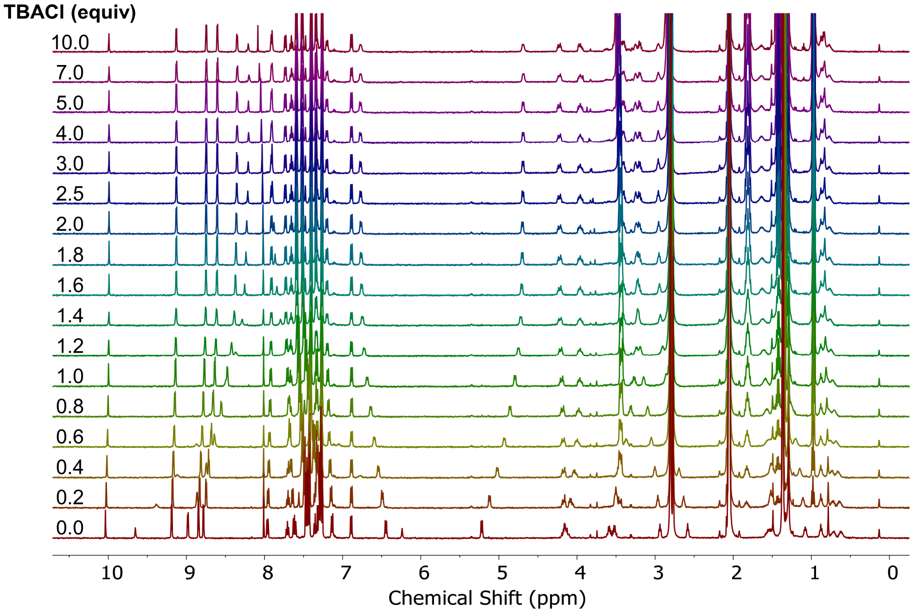

Figure S5-2. ^{1}H NMR titration spectra of **2.Zn** upon addition of 10 equivalents of TBACl ([**2.Zn**] = 1.0 mM, 500 MHz, 298 K, acetone-d_6).

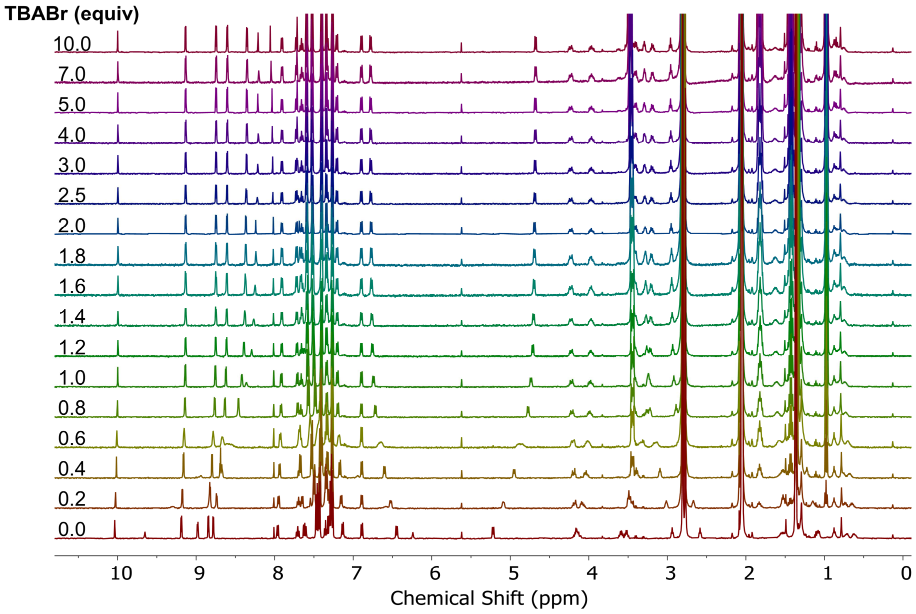

Figure S5-3. ^{1}H NMR titration spectra of **2.Zn** upon addition of 10 equivalents of TBABr ([**2.Zn**] = 1.0 mM, 500 MHz, 298 K, acetone-d_6).

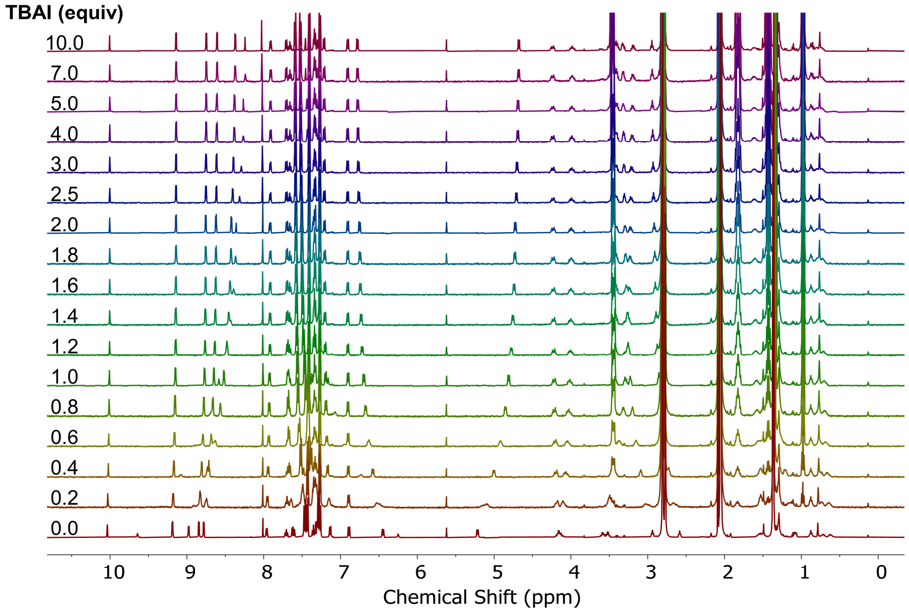

Figure S5-4. ^{1}H NMR titration spectra of **2.Zn** upon addition of 10 equivalents of TBAI ([**2.Zn**] = 1.0 mM, 500 MHz, 298 K, acetone-d_6).

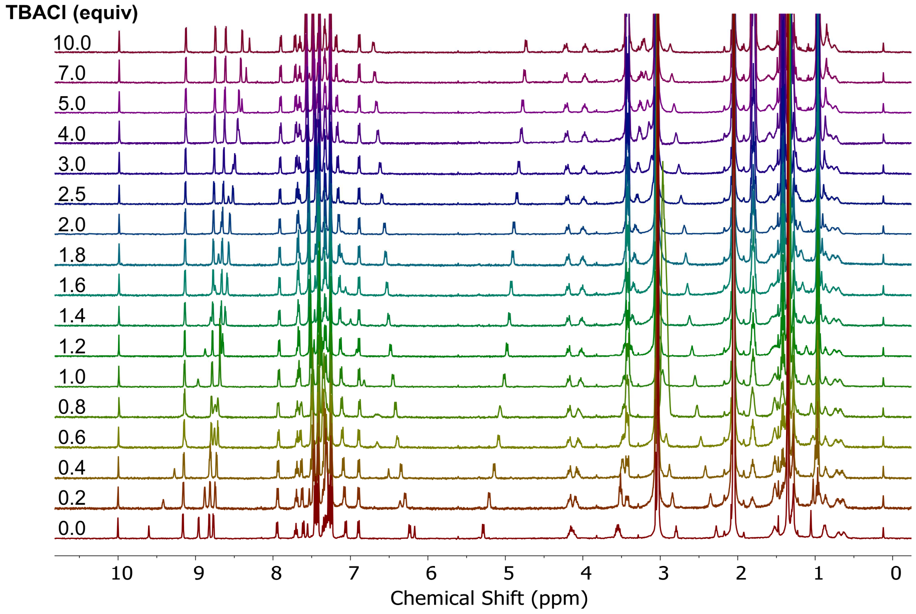

Figure S5-5. ^{1}H NMR titration spectra of **2.Zn** upon addition of 10 equivalents of TBACl ([**2.Zn**] = 1.0 mM, 500 MHz, 298 K, acetone-d_6).

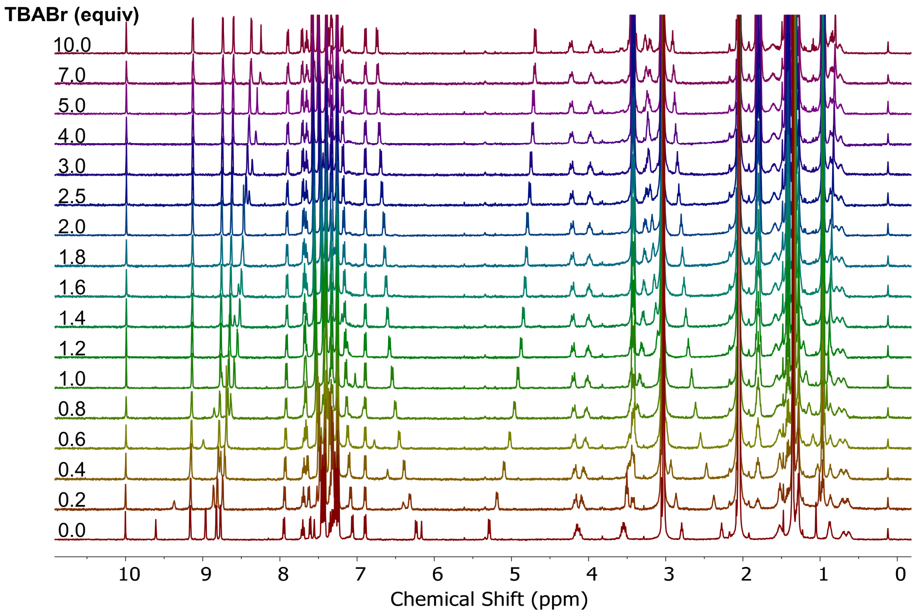

Figure S5-6. ^{1}H NMR titration spectra of **2.Zn** upon addition of 10 equivalents of TBABr ([**2.Zn**] = 1.0 mM, 500 MHz, 298 K, acetone-d_6).

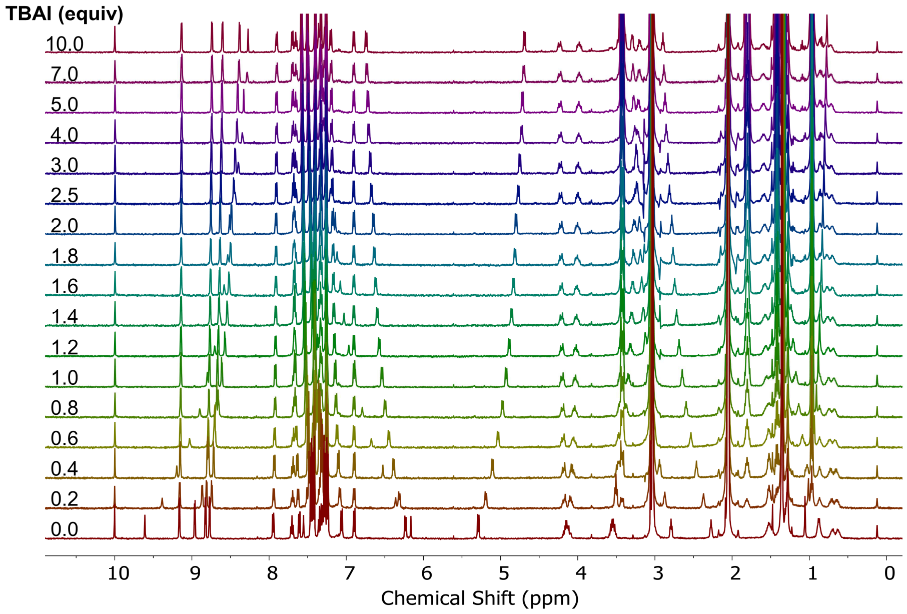

Figure S5-7. ^{1}H NMR titration spectra of **2.Zn** upon addition of 10 equivalents of TBAI ([**2.Zn**] = 1.0 mM, 500 MHz, 298 K, acetone-d_6).

^{1}H NMR Binding Isotherms

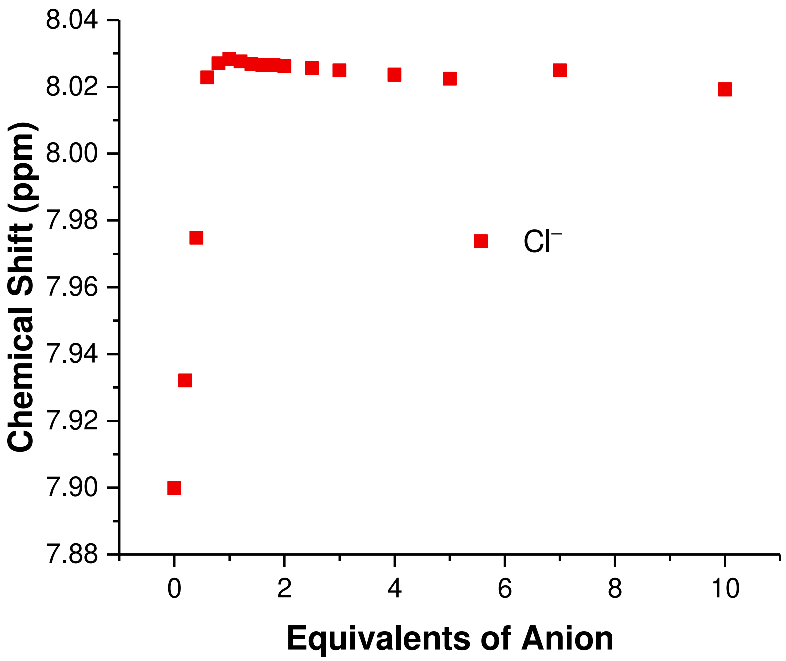

Figure S5-8. Binding isotherms of **1a.Zn** showing changes in chemical shift of internal benzene proton *c* with increasing equivalents of chloride. ([**1a.Zn**] = 1.0 mM, 500 MHz, 298 K, acetone-d_6).

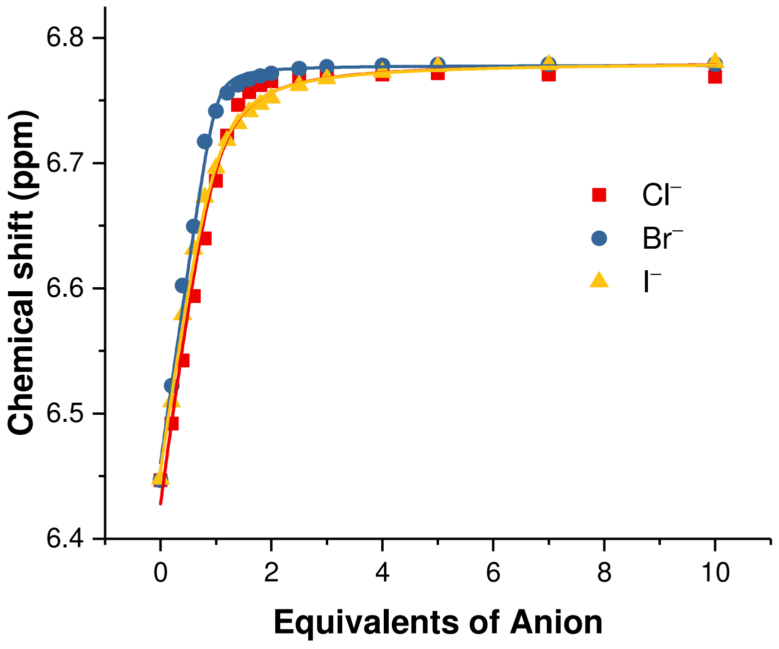

Figure S5-9. Binding isotherms of **2.Zn** showing changes in chemical shift of axle triazole proton *6* with increasing equivalents of anions. ([**2.Zn**] = 1.0 mM, 500 MHz, 298 K, acetone-d_6).

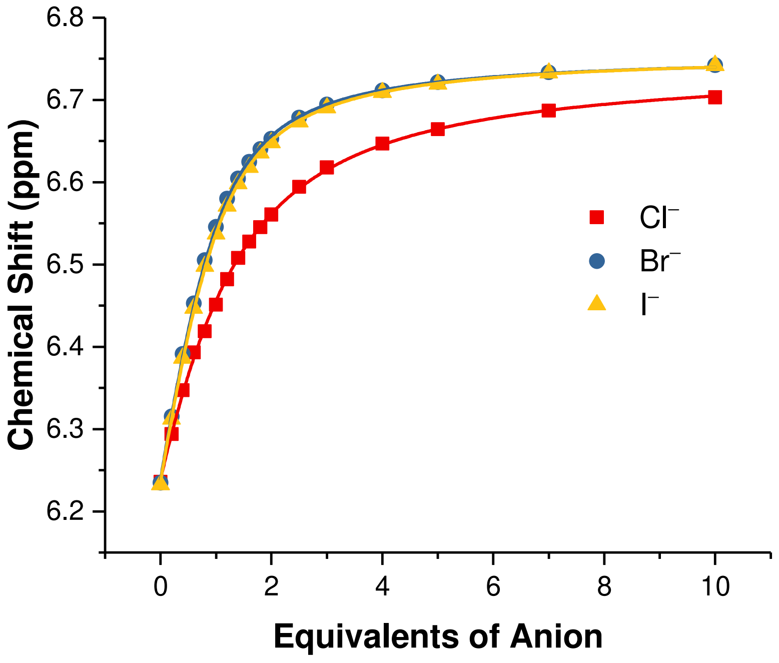

Figure S5-10. Binding isotherms of **2.Zn** showing changes in chemical shift of axle triazole proton *6* with increasing equivalents of anions. ([**2.Zn**] = 1.0 mM, 500 MHz, 298 K, 2% D_2O/acetone-d_6).

S6 UV-Visible Anion Binding Studies

General Procedures

UV-visible anion titration experiments were performed using a Horiba Duetta at 298 K. The host molecule **1a.Zn** was dissolved in acetone to give a concentration of 3.0 μM. The TBA salt of the anion was dissolved in the solution of the host molecule to obtain a concentration of 0.3 mM. Aliquots of the anion solution were added to 1.0 mL of the host solution in a quartz cuvette, where the sample was then thoroughly mixed before UV-visible spectra were recorded.

Stability constants were obtained by global analysis using OriginLab,[9,10] using a host-guest 1:1 binding model. The theoretical binding isotherms and calculated concentration profiles of the complexes were compared with the experimental data to ensure validity of the model used. For the anion titrations, the recorded spectra during the titration are shown, followed by the global fitting binding isotherms obtained by monitoring the changes in the absorbance (λ_{abs} = 380–414 nm). Filled dots represent the experimental data, while the solid lines show the fitted data (calculated isotherm). The arrows on the recorded spectra denote the direction of change in absorbance (increase or decrease) on anion addition.

UV-Visible Titration Sepctra

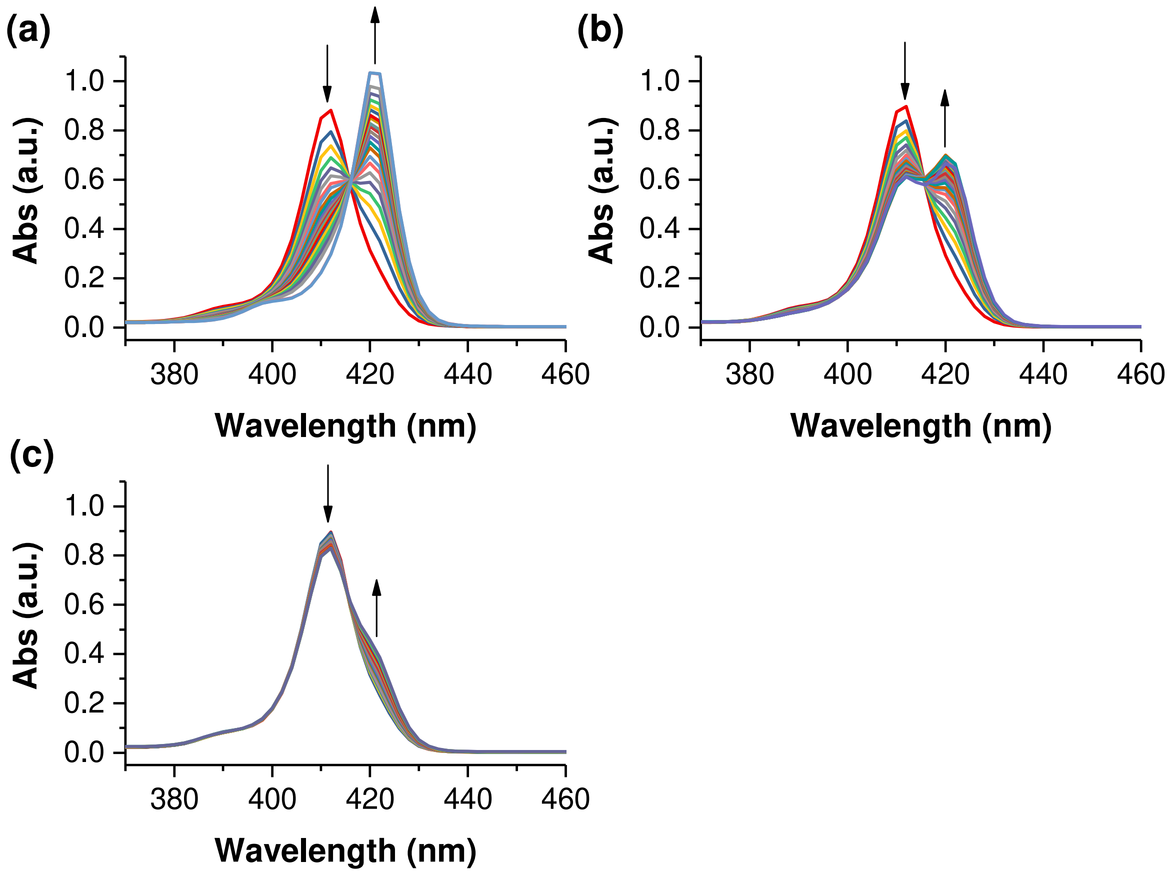

Figure S6-1. UV-visible titration spectra of **1a•Zn** showing changes in porphyrin Soret band absorbance upon addition of 10 equivalents of (a) TBACl, (b) TBABr and (c) TBAI ([**1a.Zn**] = 3 μM, 298 K, acetone).

UV-Visible Binding Isotherms

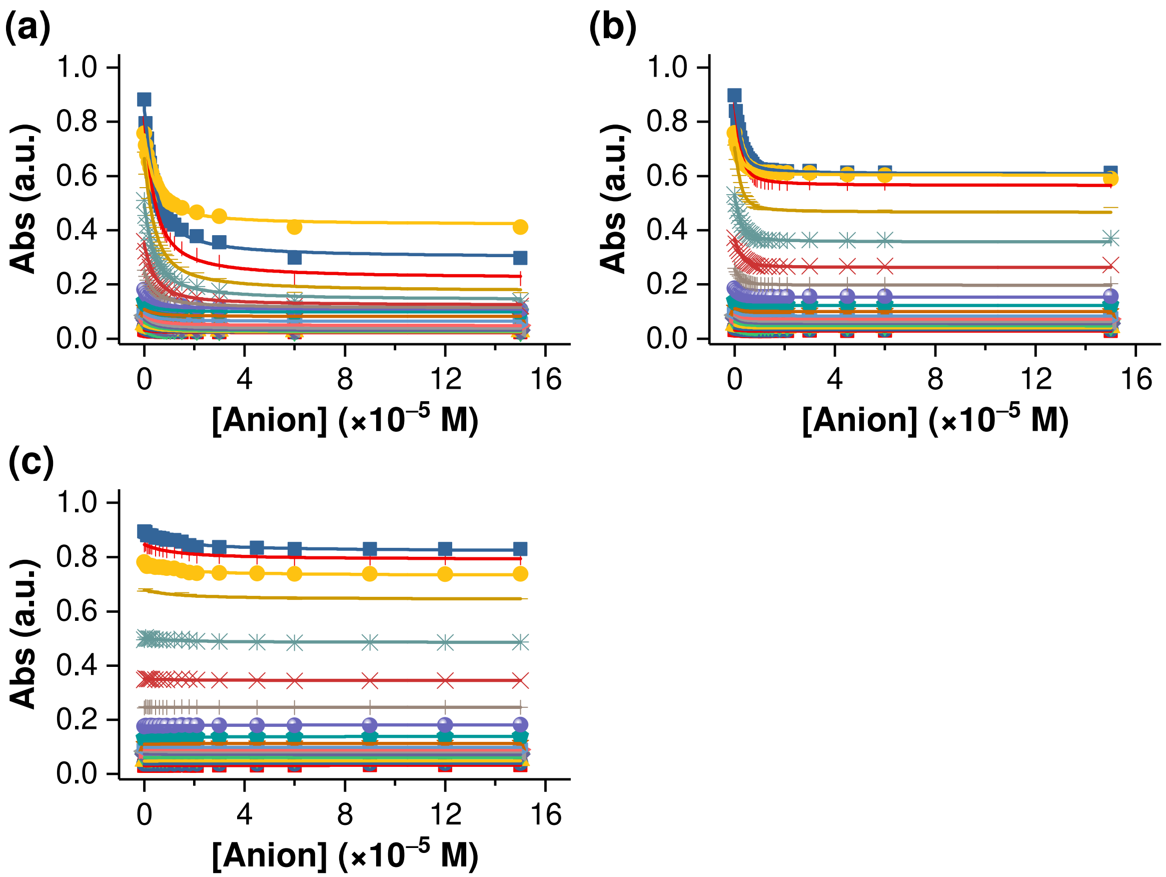

Figure S6-2. Global fitting of UV-visible titration data of **1a.Zn** with (a) TBACl, (b) TBABr and (c) TBAI in the range of 380–414 nm ([**1a.Zn**] = 3 µM, 298 K, acetone).

S7 Fluorescence Anion Binding Studies

General Procedures

Fluorescence anion titration experiments were performed using a Horiba Duetta at 298 K. The host molecules **3–4.Zn** were dissolved in acetone or H_2O/acetone (2% v/v) to give a concentration of 1.0 μM. The TBA salt of the anion was dissolved in the solution of the host molecule to obtain a concentration of 1.0 mM. Aliquots of the anion solution were added to 1.0 mL of the host solution in a quartz cuvette, where the sample was then thoroughly mixed before fluorescence spectra were recorded (λ_{ex} = 575 nm and 595 nm for **3.Zn** and **4.Zn** respectively).

Stability constants were obtained by global analysis using OriginLab,[9,10] using a host-guest 1:1 binding model. The theoretical binding isotherms and calculated concentration profiles of the complexes were compared with the experimental data to ensure validity of the model used. For the anion titrations, the recorded spectra during the titration are shown, followed by the global fitting binding isotherms obtained by monitoring the changes in the emission intensities (λ_{em}= 620-650 nm and 650-680 nm for **3.Zn** and **4.Zn** respectively). Filled dots represent the experimental data, while the solid lines show the fitted data (calculated isotherm). The arrows on the recorded spectra denote the direction of change in fluorescence intensity (increase or decrease) on anion addition.

Fluorescence Titration Spectra

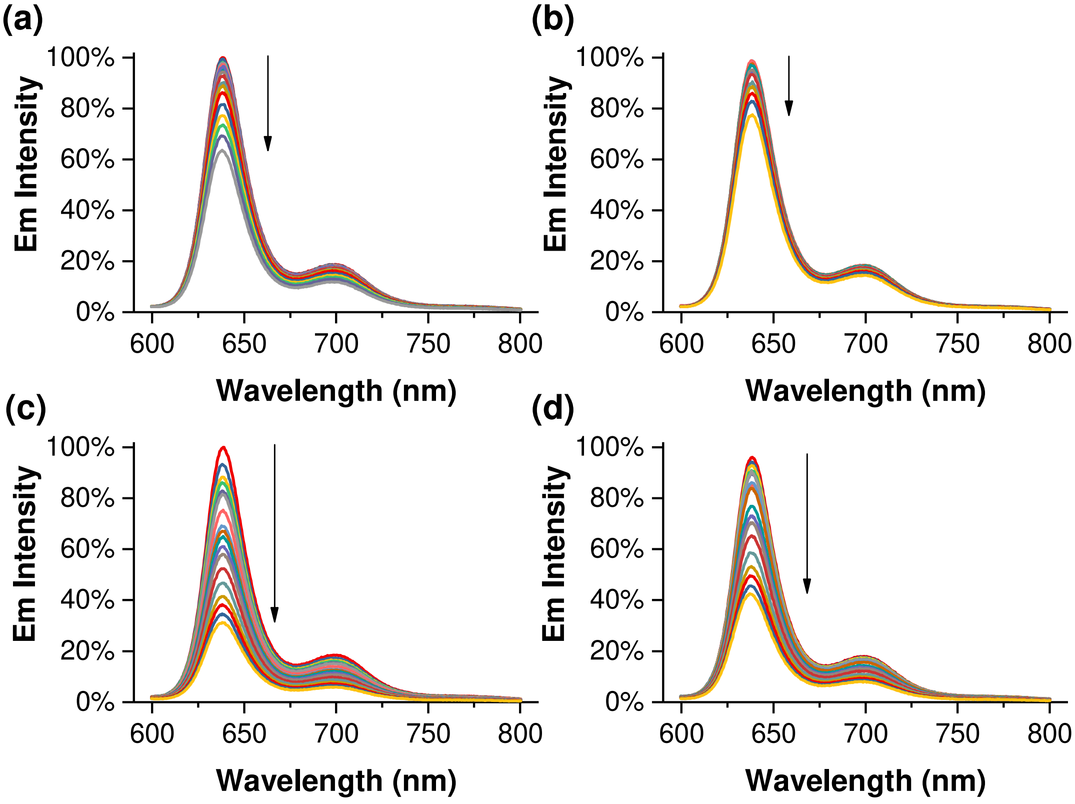

Figure S7-1. Fluorescence titration spectra of **3•Zn** upon addition of 500 equivalents of (a) TBACl, (b) TBABr, (c) TBAOAc and (d) TBA_2SO_4 ([**3.Zn**] = 1 μM, λ_{ex} = 575 nm, 298 K, acetone).

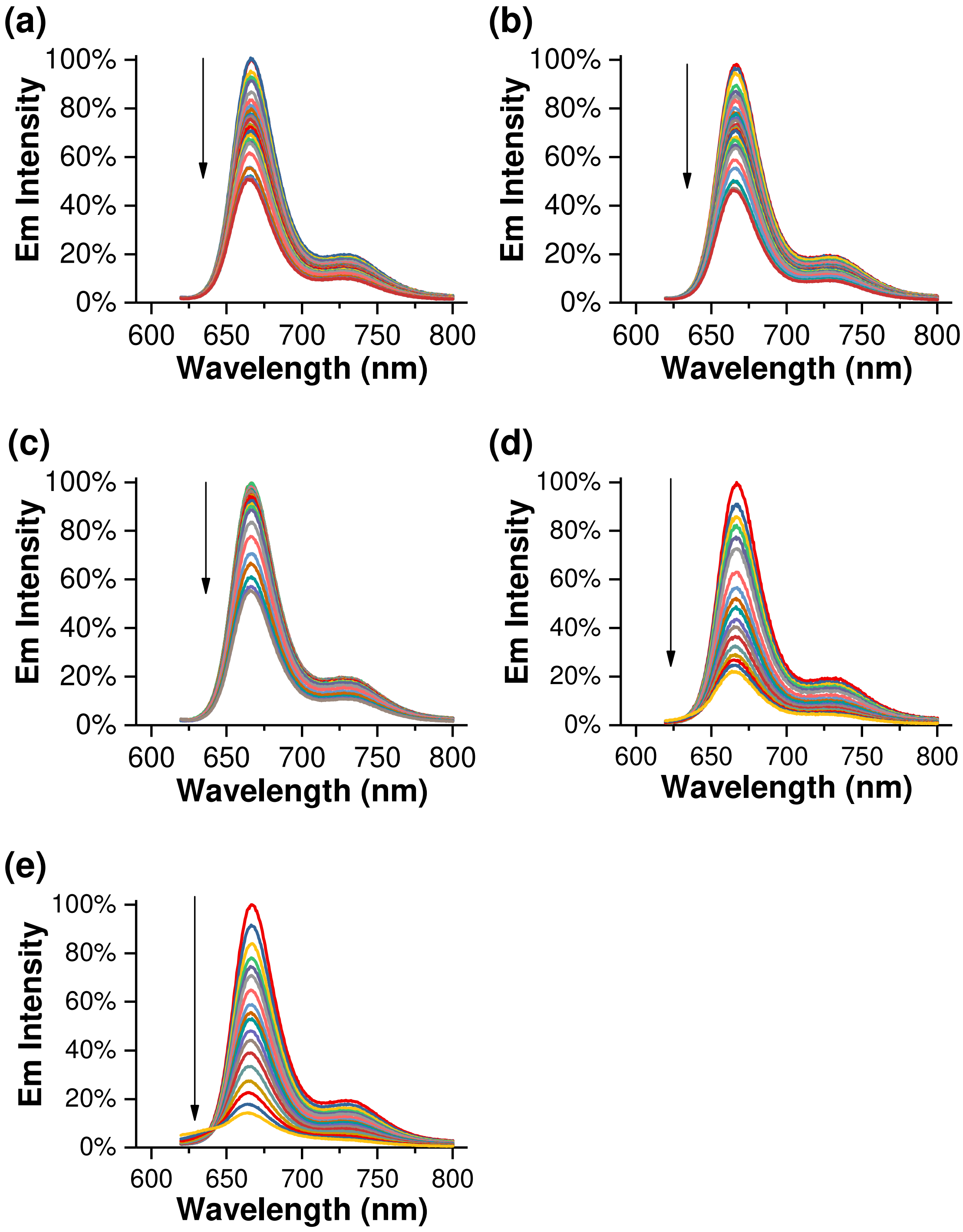

Figure S7-2. Fluorescence titration spectra of **4•Zn** upon addition of 500 equivalents of (a) TBACl, (b) TBABr, (c) TBAI, (d) TBAOAc and (e) TBA_2SO_4 ([**4.Zn**] = 1 μM, λ_{ex} = 595 nm, 298 K, acetone).

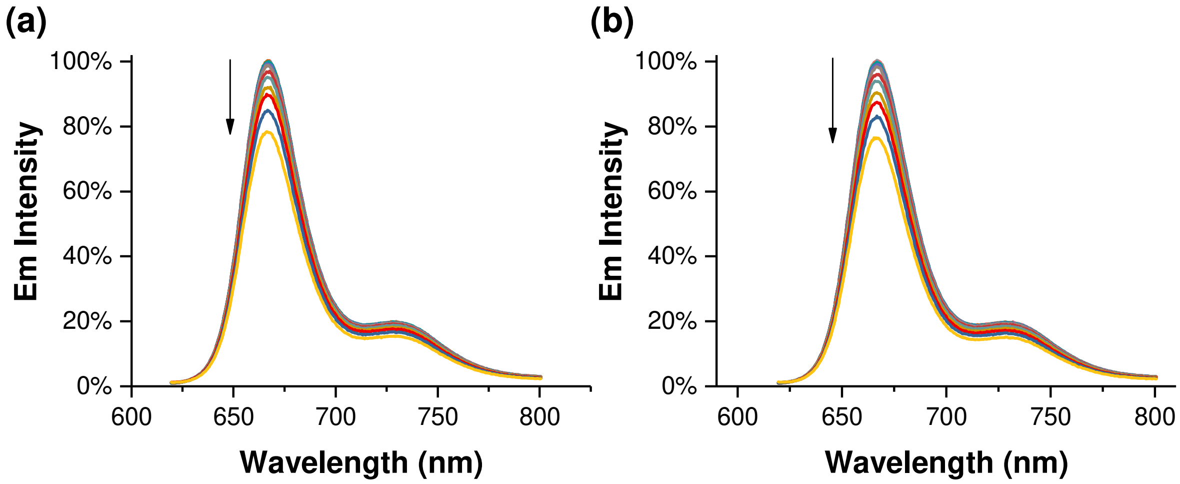

Figure S7-3. Fluorescence titration spectra of **4•Zn** upon addition of 500 equivalents of (a) TBACl and (b) TBABr ([**4.Zn**] = 1 μM, λ_{ex} = 595 nm, 298 K, 2% H_2O/acetone).

Fluorescence Binding Isotherms

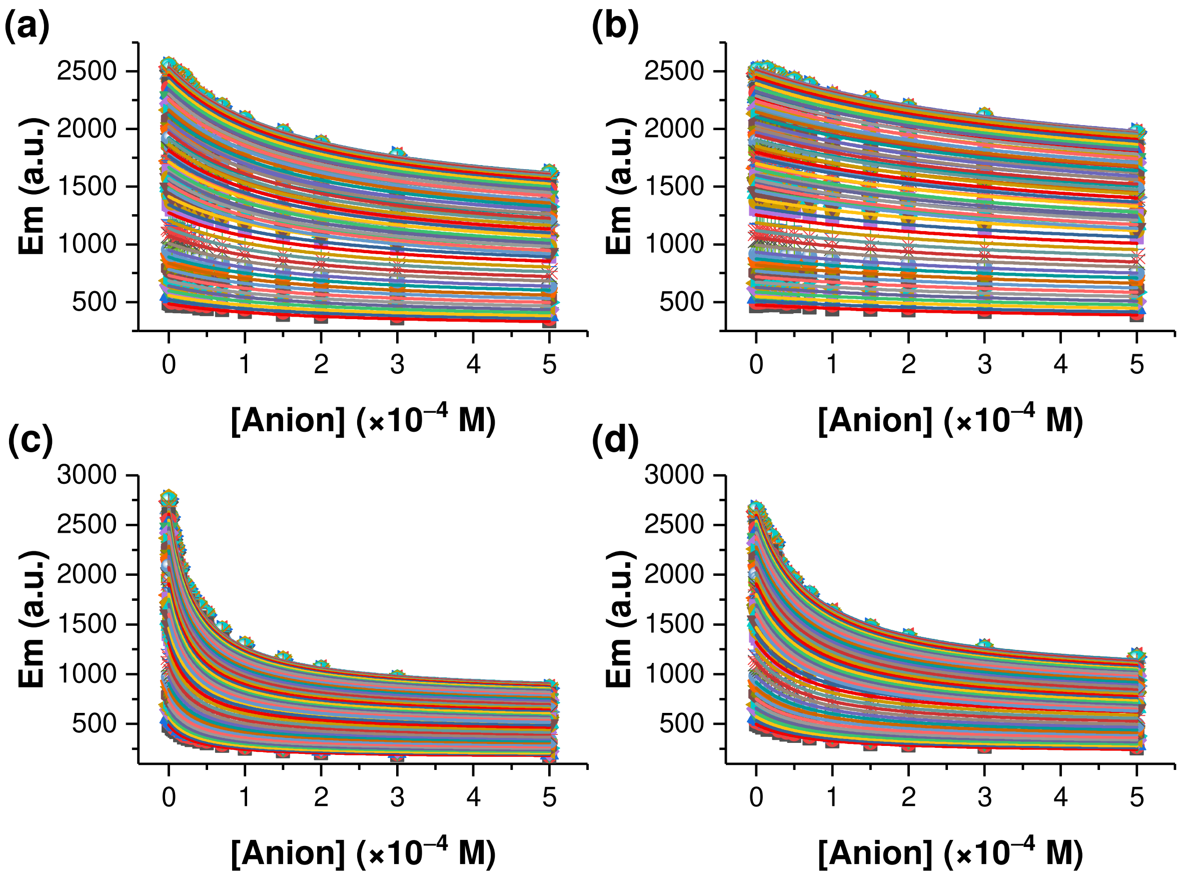

Figure S7-4. Global fitting of fluorescence titration data of **3.Zn** with (a) TBACl, (b) TBABr, (c) TBAOAc and (d) TBA_2SO_4 in the range of 620–650 nm ([**3.Zn**] = 1 μM, λ_{ex} = 575 nm, 298 K, acetone).

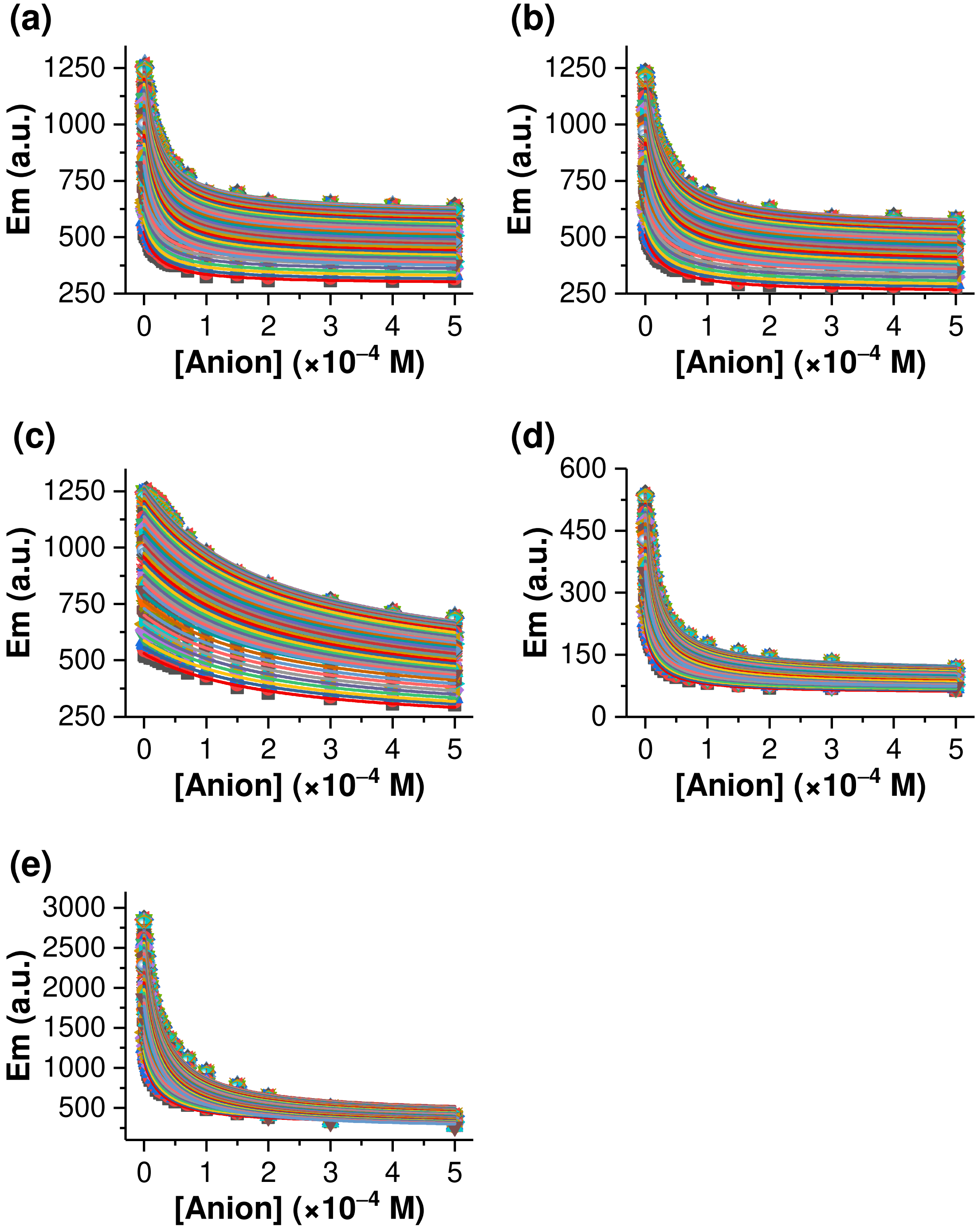

Figure S7-5. Global fitting of fluorescence titration data of **4.Zn** with (a) TBACl, (b) TBABr, (c) TBAI, (d) TBAOAc and (d) TBA_2SO_4 in the range of 650–680 nm ([**4.Zn**] = 1 μM, λ_{ex} = 595 nm, 298 K, acetone).

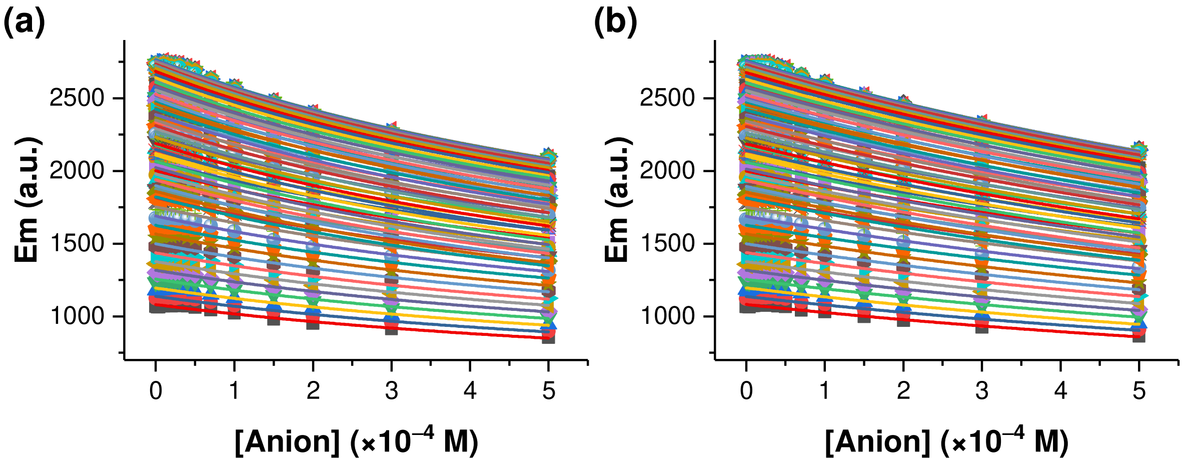

Figure S7-6. Global fitting of fluorescence titration data of **4.Zn** with (a) TBACl and (b) TBABr in the range of 650–680 nm ([**4.Zn**] = 1 µM, λ_{ex} = 595 nm, 298 K, 2% H_2O/acetone).

S8 Electrochemical Studies

General Procedures

All experiments were conducted on an Autolab potentiostat (Metrohm) in a three electrode set up. As working electrode a glassy carbon electrode was used (3 mm diameter, BASi), which was polished mechanically with alumina slurry (0.05 µm, Buehler) followed by sonication in $EtOH/H_2O$. The counter electrode was a Pt wire, while as reference electrode a leak-free Ag|AgCl electrode (Innovative Instruments Ltd.) or a non-aqueous $Ag|AgNO_3$ reference electrode (with an inner filling solution of 10 mM $AgNO_3$ in CH_3CN, 100 mM $TBAPF_6$) were used (the latter for all anion titrations). All potentials are reported with respect to Fc/Fc^+ as internal standard.

CVs were recorded using a step potential of 2.4 mV and at a scan rate of 100 mV/s, unless otherwise noted. The electrochemical reversibility was assessed by recording CVs at varying scan rates (25, 50, 75, 100, 200, 400, 600 and 800 mV/s). SWVs were recorded using a step potential of 2 mV, a 20 mV amplitude and a frequency of 25 Hz. All electrochemical titrations were followed by SWV and half-wave potentials were obtained as the peak potential from SWV. In all cases 100 mM $TBAPF_6$ was used as supporting electrolyte. In anion sensing studies both the overall host concentration (0.1 mM) as well as the total ionic strength was kept constant (100 mM; by titration of an initial host solution containing 100 mM $TBAPF_6$ titrated with a solution containing 100 mM TBA-anion and 0.1 mM host).

S8.1 General Characteristics and Reversibility

A well-defined oxidative redox wave, ascribable to the one-electron oxidative porphyrin redox couple $P/P^{+\bullet}$ was observed at moderate potentials. From CVs at varying scan rates it was ascertained that this redox couple, as well as the BODIPY redox couple, typically shows a high degree of reversibility (Figure S8-1-S8-7) with a near unity ratio of anodic and cathodic peak currents and linear dependence of the peak currents on the square-root of the scan rate. This indicated a diffusion-controlled redox process with minimal adsorption of the receptor onto the glassy carbon working electrode.

At more anodic potentials an additional redox couple, arising from further one-electron oxidation ($P^{+\bullet}/P^{2+}$) was observed (see Table S8-1), however for all receptors this couple displayed poorer reversibility and was not studied in more detail.

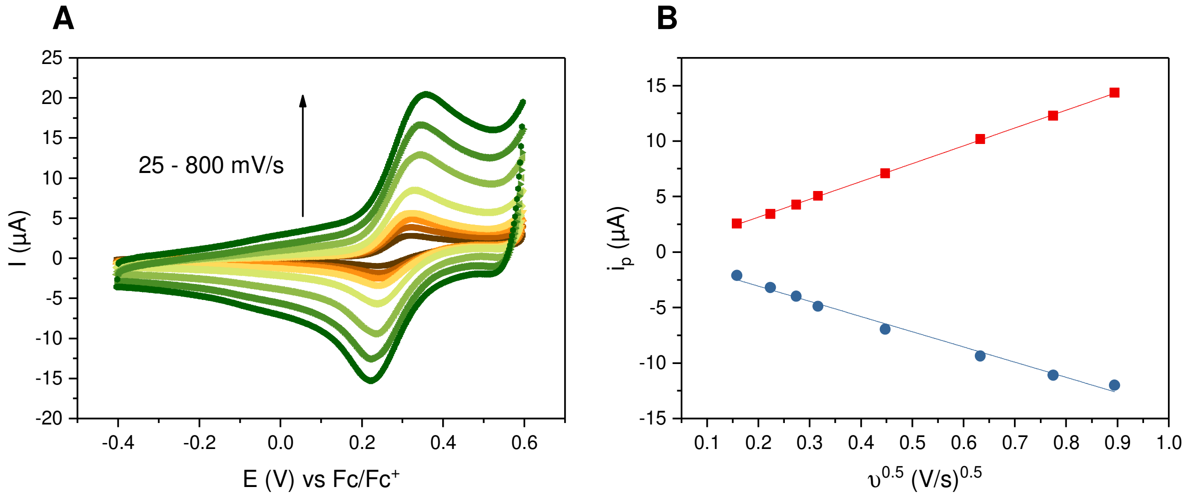

Figure S8-1. CVs (A) and anodic and cathodic peak currents (B) of the P/P$^{+\bullet}$ couple of 0.25 mM **1a.Zn** in CH_2Cl_2, 100 mM $TBAPF_6$ at varying scan rates.

Compound **1b.Zn**, with the longer strap displays generally similar voltammetry (Figure S8-2A), with a slightly compromised reversibility, as evidenced by the ratio of peak currents $i_{pa}/i_{pc} \approx 1.6$ (Figure S8-2B).

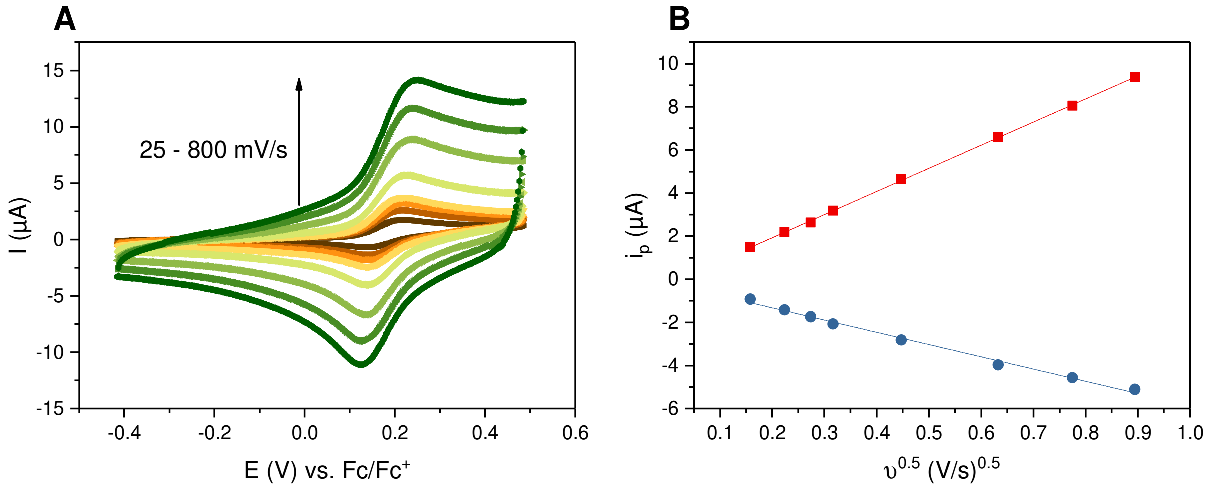

Figure S8-2. CVs (A) and anodic and cathodic peak currents (B) of the P/P$^{+\bullet}$ couple of 0.25 mM **1b.Zn** in CH_2Cl_2, 100 mM $TBAPF_6$ at varying scan rates.

Gratifyingly, even the interlocked receptor **2.Zn** displayed a well-defined, quasi-reversible P/P$^{+\bullet}$ couple as shown in Figure S8-3.

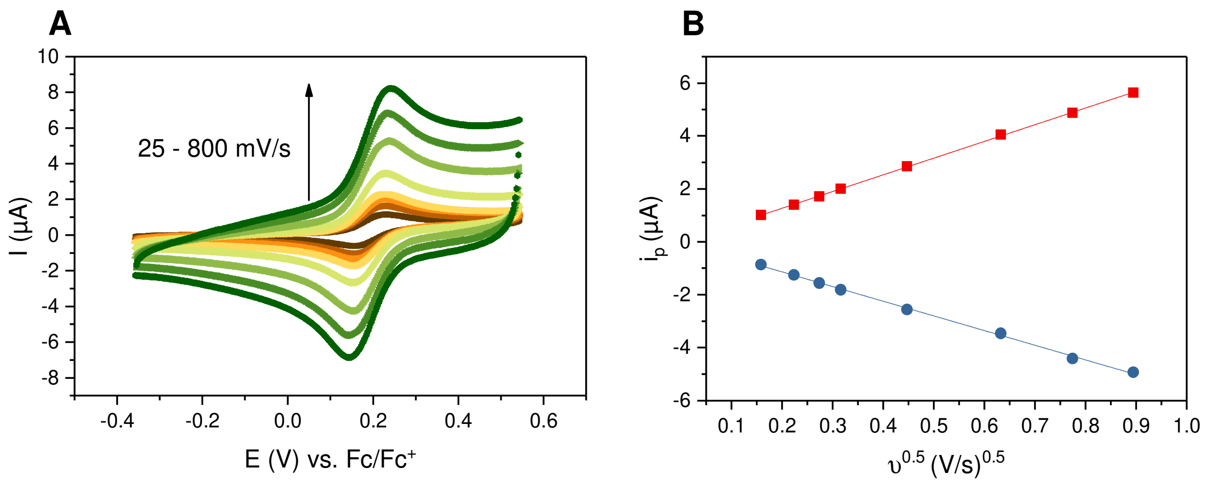

Figure S8-3. CVs (A) and anodic and cathodic peak currents (B) of the P/P$^{+•}$ couple of 0.25 mM **2.Zn** in CH_2Cl_2, 100 mM $TBAPF_6$ at varying scan rates.

The BODIPY-stoppered rotaxane **4.Zn** displayed, in addition to a quasi-reversible P/P$^{+•}$ couple, another quasi-reversible redox couple at slightly more anodic potentials, as shown in Figure S8-4. This redox process can, by comparison with the stopper BODIPY azide **11**, be unambiguously assigned to a BODIPY centred oxidation yielding the monocationic radical (BDP/BDP$^{+•}$; see Table S8-1, Figure S8-5 and Figure S8-8A). Interestingly, this oxidative couple displayed a very high degree of reversibility in both **11** as well as rotaxane **4.Zn** (Figures S8-4 and S8-5).

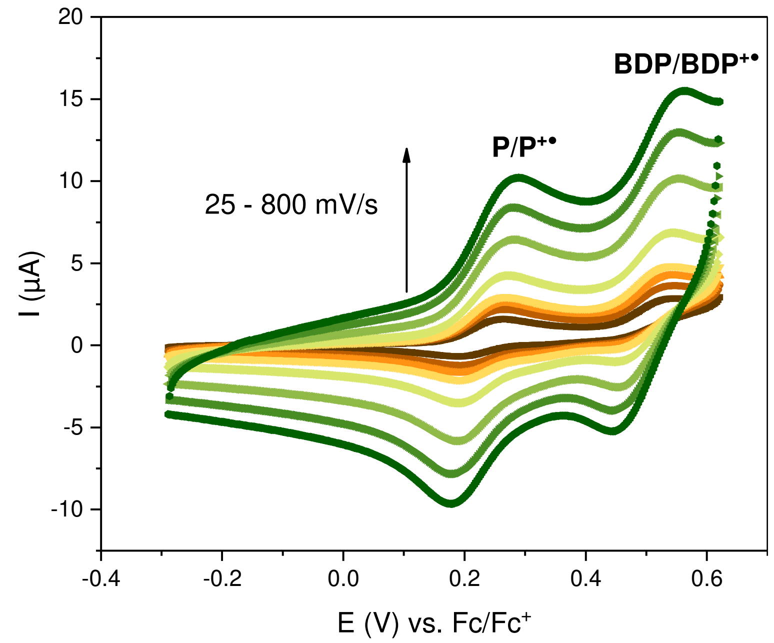

Figure S8-4. CVs of 0.25 mM **4.Zn** in CH_2Cl_2, 100 mM $TBAPF_6$ at varying scan rates.

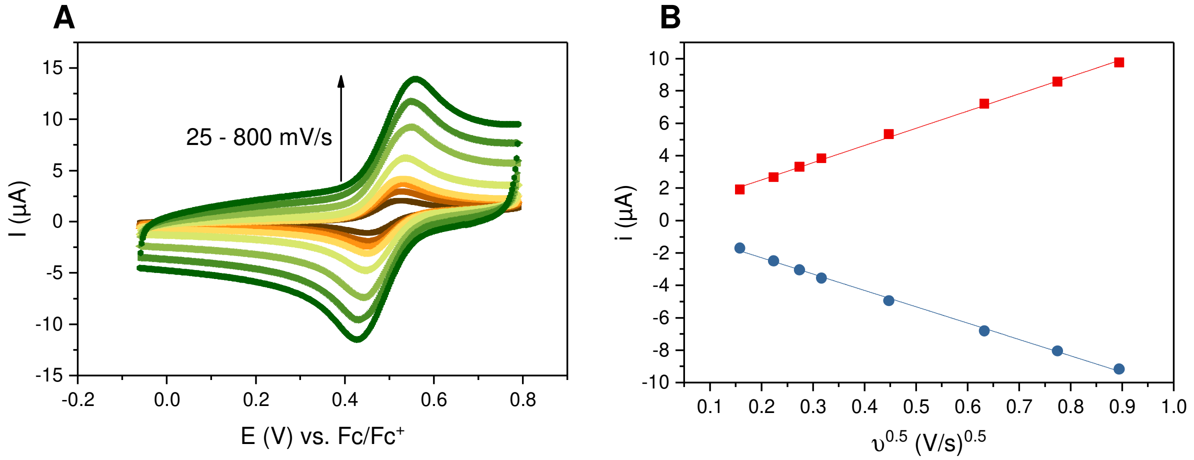

Figure S8-5. CVs (A) and anodic and cathodic peak currents (B) of 0.25 mM **11** in CH_2Cl_2, 100 mM $TBAPF_6$ at varying scan rates.

Rotaxane **3.Zn**, containing the opposite axle-triazole connectivity and no perfluoro-benzene linker, also displayed two oxidative redox couples (Figure S8-6A), which can again, by comparison with the BODIPY-alkyne precursors **9**, be assigned to subsequent one-electron porphyrin and BODIPY oxidation (Figures S8-7 and S8-8B). Interestingly, the former couple

displays relatively poor reversibility (Figure S8-6B), while the BDP/BDP$^{+\bullet}$ couple remains quasi-reversible, in both **3** and **9** (Figures S8-6B and S8-7B). The origins of this comparably poor reversibility of the P/P$^{+\bullet}$ couple in only **3.Zn** are unclear.

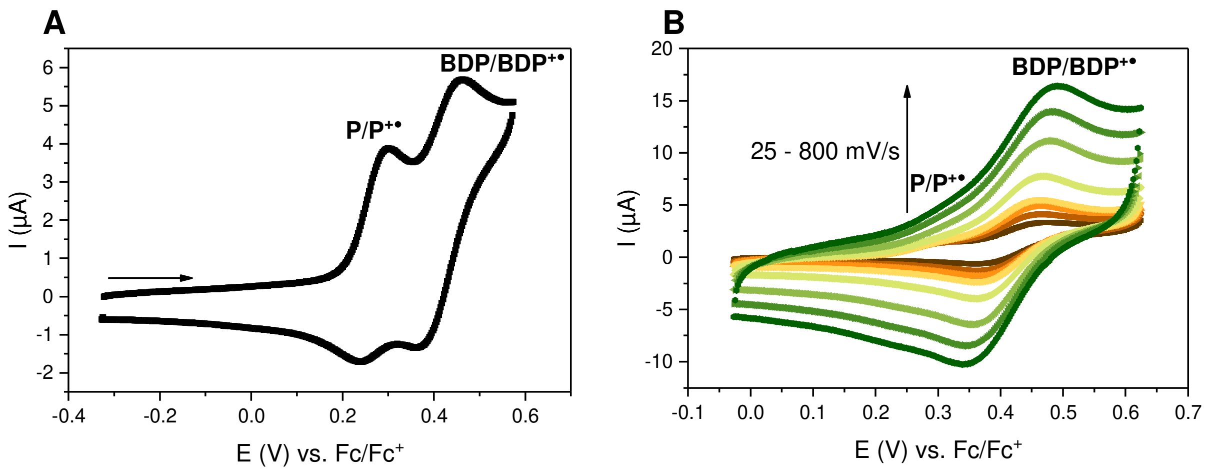

Figure S8-6. CV at 100 mV/s (first scan) (A) and anodic and cathodic peak currents (B) of 0.25 mM **3.Zn** in CH_2Cl_2, 100 mM $TBAPF_6$ at varying scan rates.

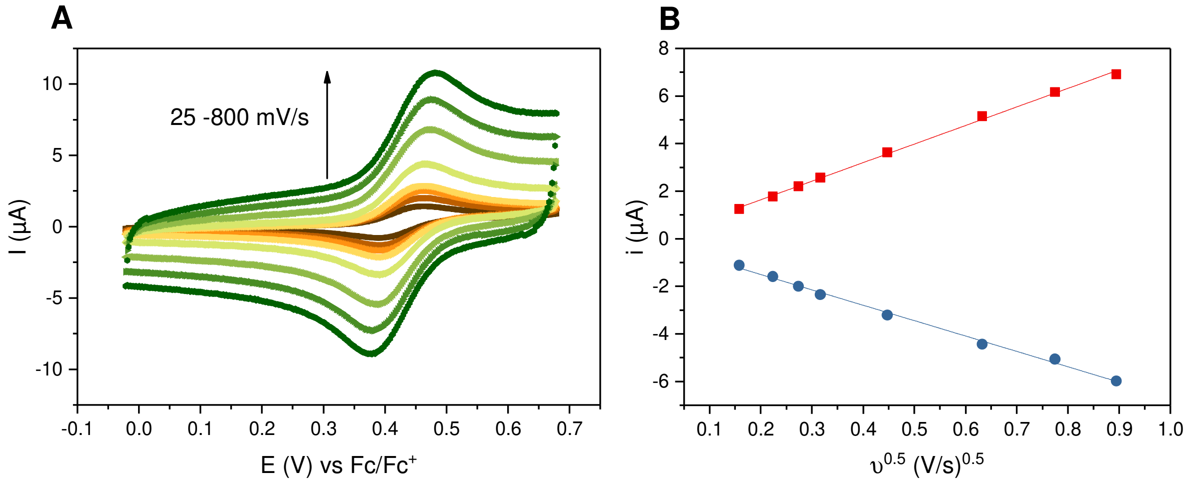

Figure S8-7. CVs (A) and anodic and cathodic peak currents (B) of 0.25 mM **9** in CH_2Cl_2, 100 mM $TBAPF_6$ at varying scan rates.

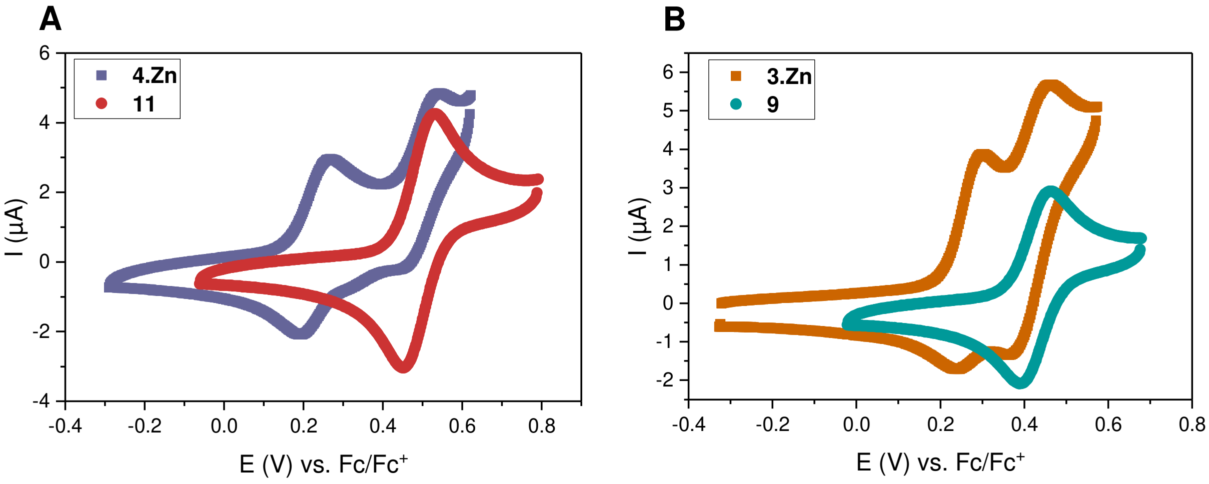

Figure S8-8. CVs of A) **4.Zn** (purple) and **11** (wine red) and B) **3.Zn** (purple) and **9** (cyan) in CH_2Cl_2/$TBAPF_6$ at a scan rate of 100 mV/s.

S8.2 Comparison of Half-wave Potentials

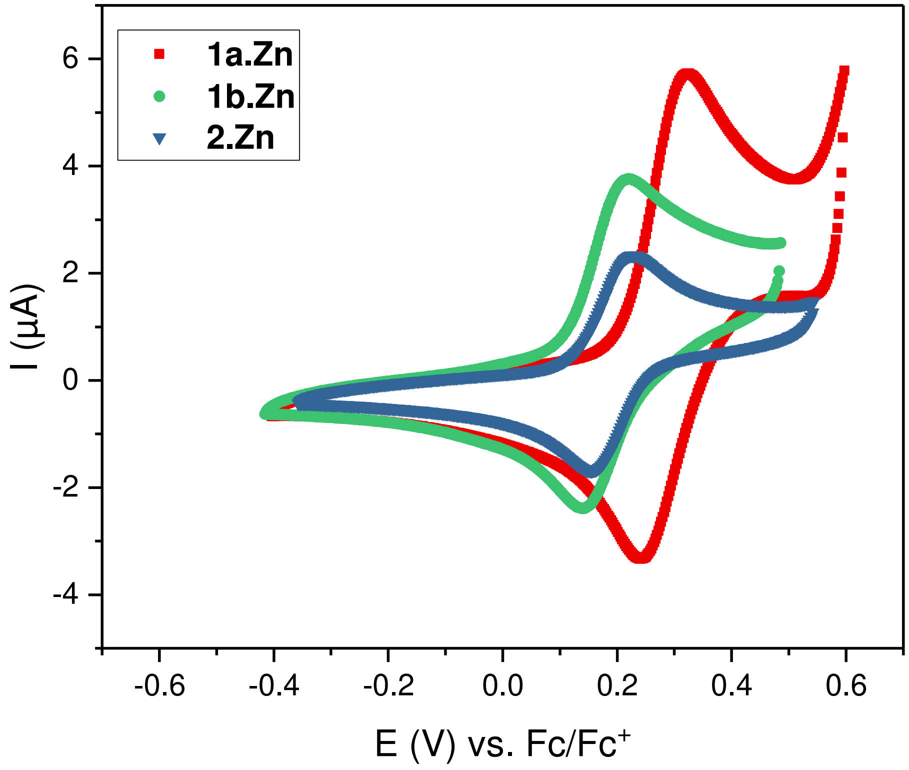

Figure S8-9. CVs of **1a.Zn** (red), **1b.Zn** (green) and **2.Zn** (blue) in CH_2Cl_2/$TBAPF_6$ at a scan rate of 100 mV/s.

Table S8-1. Half-wave potentials $E_{1/2}$ (V vs. Fc/Fc^{+}) for all tested compounds in CH_2Cl_2, 100 mM $TBAPF_6$ in the absence and presence of pyridine (1% (v/v)). / - not applicable. N.o. – not observed.

$E_{1/2}$ in $CH_2Cl_2/TBAPF_6$	$P/P^{+\cdot}$	$P^{+\cdot}/P^{2+}$	$BDP/BDP^{+\cdot}$
**1a.Zn**	0.282 V	0.624 V	/
**1b.Zn**	0.179 V	0.617 V	/
**2.Zn**	0.193 V	0.652 V	/
**4.Zn**	0.230 V	0.675 V	0.501 V
**11**	/	/	0.491 V
**3.Zn**	0.270 V	0.688 V	0.425 V
**9**	/	/	0.426 V
**1a.Zn + 1% (v/v) Pyridine**	0.267 V	0.567 V	/
**1b.Zn + 1% (v/v) Pyridine**	0.270 V	0.552 V	/
**2.Zn + 1% (v/v) Pyridine**	0.204 V	0.550 V	/
**4.Zn + 1% (v/v) Pyridine**	0.248 V	0.579 V	≈0.47[b]
**3.Zn + 1% (v/v) Pyridine**	0.292 V	0.609 V	n.o.

a) Poor reversibility. b) weak and broad.

.

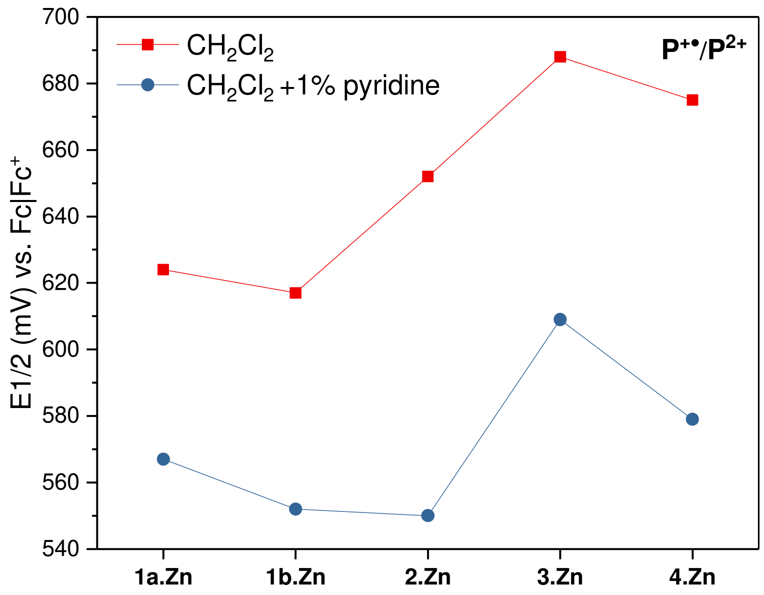

Figure S8-10. Half-wave potentials of the $P^{+\bullet}/P^{2+}$ couple of all receptors in $CH_2Cl_2/TBAPF_6$ in the absence (red square) and presence (blue circles) of 1% (v/v) pyridine. Connecting lines are to guide the eye only.

S8.3 Electrochemical Anion Sensing Studies

All voltammetric binding isotherms were fitted according to eqn. 1, from which absolute binding constants to both the oxidized (K_{Ox}) and reduced (native) receptor state were obtained (K_{Red}), see Table 5:[11]

$$\Delta E = -\frac{RT}{nF}\ln\left(\frac{1+K_{Ox}[A^-]}{1+K_{Red}[A^-]}\right) \qquad \text{eqn. 1}$$

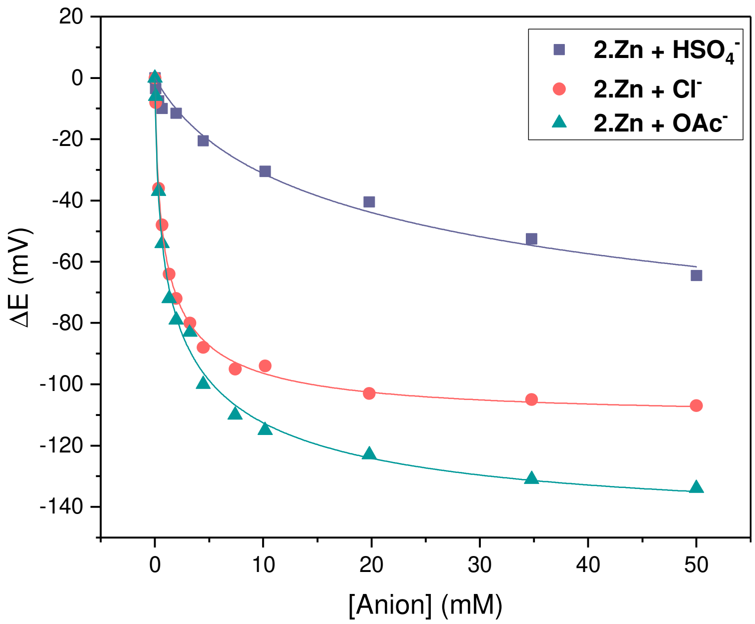

Figure S8-11. Cathodic voltammetric shifts of **2.Zn** upon titration with HSO_4^- (purple squares), Cl^- (pink circles) and OAc^- (cyan triangles) in CH_2Cl_2, 100 mM $TBAPF_6$. Solid lines represent fits to the 1:1 Nernst model (eqn. 1).

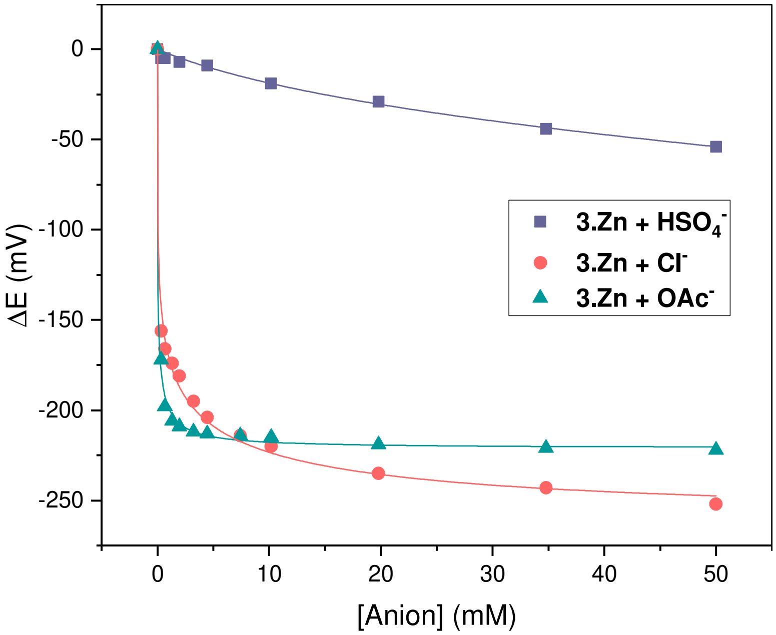

Figure S8-12. Cathodic voltammetric shifts of **3.Zn** upon titration with HSO_4^- (purple squares), Cl^- (pink circles) and OAc^- (cyan triangles) in CH_2Cl_2, 100 mM $TBAPF_6$. Solid lines represent fits to the 1:1 Nernst model (eqn. 1).

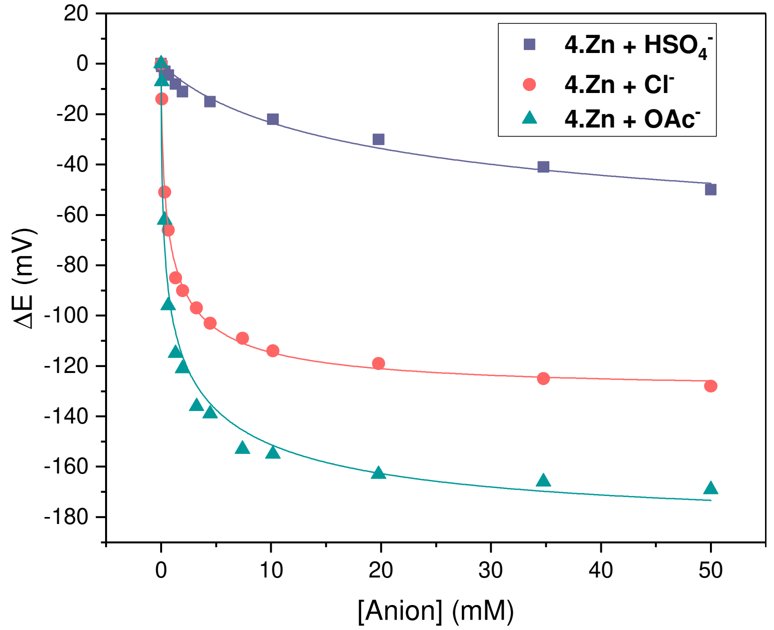

Figure S8-13. Cathodic voltammetric shifts of **4.Zn** upon titration with HSO_4^- (purple squares), Cl^- (pink circles) and OAc^- (cyan triangles) in CH_2Cl_2, 100 mM $TBAPF_6$. Solid lines represent fits to the 1:1 Nernst model (eqn. 1).

S9 References

[1] J. E. Hein, L. B. Krasnova, M. Iwasaki, V. V. Fokin, in *Organic Syntheses* (Ed.: John Wiley & Sons, Inc.), John Wiley & Sons, Inc., Hoboken, NJ, USA, **2011**, pp. 238–246.

[2] A. Borissov, J. Y. C. Lim, A. Brown, K. E. Christensen, A. L. Thompson, M. D. Smith, P. D. Beer, *Chem. Commun.* **2017**, *53*, 2483–2486.

[3] S. Purushothaman, R. Prasanna, R. Raghunathan, *Tetrahedron* **2013**, *69*, 9742–9750.

[4] H. Nate, K. Matsuki, A. Tsunashima, H. Ohtsuka, Y. Sekine, K. Oda, Y. Honma, A. Ishida, H. Nakai, H. Wada, M. Takeda, H. Yabana, Y. Hino, T. Nagao, *Chemical & Pharmaceutical Bulletin* **1987**, *35*, 2394–2411.

[5] T. Zhang, X. Dong, H. B. Jalani, J. Zou, G. Li, H. Lu, *Org. Lett.* **2019**, *21*, 3706–3710.

[6] Z. Li, R. Bittman, *J. Org. Chem.* **2007**, *72*, 8376–8382.

[7] G. M. Sheldrick, *Acta Crystallogr A Found Crystallogr* **2008**, *64*, 112–122.

[8] “Supramolecular.org - Binding Constant Calculators | Supramolecular,” can be found under http://supramolecular.org/

[9] P. Thordarson, *Chem. Soc. Rev.* **2011**, *40*, 1305–1323.

[10] D. Brynn Hibbert, P. Thordarson, *Chem. Commun.* **2016**, *52*, 12792–12805.

[11] R. Hein, P. D. Beer, J. J. Davis, *Chem. Rev.* **2020**, *120*, 1888–1935.
